# Risk factors for sacrococcygeal pilonidal sinus: a systematic review and meta-analysis supplemented by genetic causal assessment

**DOI:** 10.3389/fsurg.2025.1718589

**Published:** 2026-01-07

**Authors:** Xingli Xu, Peijie You, Jialin Qin, Jiong Wu

**Affiliations:** 1 Department of Coloproctology, Yueyang Hospital of Integrated Traditional Chinese and Western Medicine, Shanghai University of Traditional Chinese Medicine, Shanghai, China; 2 Department of Orthopedics and Traumatology, Suzhou TCM Hospital Affiliated to Nanjing University of Chinese Medicine, Suzhou, China

**Keywords:** pilonidal sinus, risk factors, systematic review, meta-analysis, Mendelian randomization

## Abstract

**Objective:**

Sacrococcygeal pilonidal sinus (PS) is a common, chronic, and recurrent condition with unclear pathogenesis. Previous studies have primarily focused on local factors, with limited causal validation of systemic risk factors. This study aims to synthesize observational and genetic evidence to systematically evaluate potential risk factors and explore possible multisystem pathological mechanisms.

**Methods:**

This study primarily employed a systematic review and meta-analysis by retrieving high-quality observational studies to quantitatively evaluate the associations between various risk factors—such as behavioral habits, metabolic and immune factors, and related diseases—and PS. Heterogeneity tests, subgroup analyses, and sensitivity analyses were conducted to ensure the robustness of the results. To further validate the causal nature of these associations, a two-sample Mendelian randomization (MR) analysis based on genome-wide association study (GWAS) data was performed to explore potential genetic causal relationships.

**Results:**

A total of 19 observational studies were included, of which 12 were eligible for meta-analysis. The findings revealed that increased body mass index, sedentary behavior, dense body hair, family history, and poor hygiene were significantly associated with an elevated risk of PS. These associations were further supported by MR analyses. In addition, higher eosinophil counts and high-density lipoprotein (HDL) levels appeared to be protective factors. Conditions such as diabetes, hidradenitis suppurativa, acne, polycystic ovary syndrome, and osteoarthritis were also linked to increased PS risk. No significant associations were found between PS and smoking, alcohol consumption, sex hormones, hair color, skin color, excessive sweating, physical activity, or most lipid parameters.

**Conclusion:**

This study identified multiple risk factors for PS through meta-analysis and systematic review, and further provided genetic evidence of causality. The findings suggest that PS is not merely a localized condition but may be driven by systemic factors such as metabolic, inflammatory, and endocrine dysfunctions. These results underscore the importance of early identification of high-risk individuals and support the need for targeted lifestyle interventions.

**Systematic Review Registration:**

https://www.crd.york.ac.uk/PROSPERO/view/CRD420251057814, identifier CRD42024618778.

## Introduction

Pilonidal sinus (PS) is a chronic sinus tract or cyst that develops within the soft tissues of the sacrococcygeal natal cleft. It may also present as an acute sacrococcygeal abscess that, upon rupture, forms a chronic sinus tract or temporarily heals before subsequently recurring. The presence of entrapped hair within the lesion is a characteristic feature of the disease (1). In Western countries, the incidence of PS is approximately 26 per 100,000 individuals, predominantly affecting young adults aged 15–30 years, with males exhibiting a fourfold higher risk than females (2, 3). Despite the availability of various treatment options, PS remains prone to recurrence, and its underlying etiology is not fully understood. Previous studies have associated PS with mechanical friction, local anatomical features, hair growth patterns, and inflammatory responses (2). However, traditional research has predominantly focused on local factors, with limited causal validation of systemic risk factors. In particular, evidence regarding metabolic, immune, and endocrine influences remains insufficient. In recent years, growing research into the interplay between systemic diseases and dermatological conditions has suggested that PS may not be solely driven by local mechanical factors but also influenced by systemic disturbances involving metabolism, immunity, and endocrine regulation. Several studies have reported that PS is frequently comorbid with metabolic disorders such as obesity, diabetes, and polycystic ovary syndrome (PCOS) (4), suggesting that these conditions may contribute to PS development via shared inflammatory pathways, impaired skin barrier function, or altered local metabolism.

To uncover causal relationships among these complex disease associations, Mendelian randomization (MR) has been increasingly employed in recent years. This method uses single nucleotide polymorphisms (SNPs) associated with exposure variables as instrumental variables (IVs). By leveraging the natural random allocation of genetic variants, MR offers greater robustness against confounding and reverse causation compared to traditional observational studies (5).

This study synthesized evidence from multiple investigations through systematic review and meta-analysis to provide robust overall effect estimates, and further incorporates MR to assess the causal nature of these associations from a genetic perspective. By integrating these complementary methodologies, this study offers a more rigorous and comprehensive evaluation of potential risk factors for PS. The findings clarify the etiological determinants of PS and delineate its systemic links with metabolic traits and related comorbidities, thereby contributing to a better understanding of shared disease mechanisms and providing a theoretical foundation for interdisciplinary research and individualized prevention strategies.

## Method

This study combined systematic review, meta-analysis, and MR to investigate the potential risk factors for PS. The meta-analysis synthesized existing observational evidence, while the MR analysis explored potential causal relationships between exposures and PS from a genetic perspective, aiming to address the limitations of confounding and reverse causation inherent in conventional epidemiological studies.

### Systematic review and meta-analysis

The systematic review and meta-analysis were conducted in strict accordance with the *Cochrane Handbook for Systematic Reviews of Interventions* (version 6.2) (6). Reporting followed the PRISMA (Preferred Reporting Items for Systematic Reviews and Meta-Analyses) statement (7). The study protocol was registered in PROSPERO (Registration number: CRD42024618778; https://www.crd.york.ac.uk/PROSPERO/view/CRD420251057814).

A comprehensive literature search was conducted in PubMed, Embase, Web of Science, and the Cochrane Library from their inception to 23 June 2025, without language restrictions. The search strategy combined Medical Subject Headings (MeSH) and free-text terms, using keywords such as “Pilonidal Sinus” and “risk factors.” This study did not include a separate search of gray literature databases. The detailed search strategy is provided in Supplementary Data Sheet S1.

The inclusion criteria were defined according to the PICOS framework:

• P (Population): Patients diagnosed with PS;
• I (Intervention/Exposure): Exposure to specific risk factors (e.g., obesity, hirsutism, or sedentary behavior);
• C (Comparison): Individuals without PS serving as controls;
• O (Outcome): Association between risk factors and the occurrence of PS;
• S (Study design): Observational studies (case–control and cohort studies), with full-text publications.

The exclusion criteria were as follows: (1) duplicate publications; (2) reviews, animal studies, or non-clinical research; (3) studies with incomplete data or serious methodological flaws; and (4) articles without accessible full text.

Two reviewers (XX and PY) independently conducted literature screening and data extraction. After an initial review of titles and abstracts, full-text articles were assessed for eligibility. Discrepancies were resolved through discussion with a third reviewer (JW). Extracted data included study characteristics, sample information, exposure variables, number of events, effect estimates, and corresponding confidence intervals (CIs).

Meta-analyses were performed using RevMan 5.3 and Stata 19.0. For dichotomous variables, odds ratios (ORs) with 95% CIs were calculated. For continuous variables, mean differences (MDs) with 95% CIs were reported. Heterogeneity was assessed using the *I*^2^ statistic and chi-square test. A random-effects model was applied when *I*^2^ > 50%. Sensitivity analyses were conducted using the leave-one-out (LOO) method to evaluate the robustness of the findings (6). Publication bias was assessed using funnel plots and Egger's test in Stata (8). The quality of evidence was evaluated using the GRADE approach with GRADEpro software (9). The risk of bias in case–control studies was assessed using the Newcastle–Ottawa Scale (NOS), which evaluates selection, comparability, and exposure assessment. A score of ≥6 was considered indicative of high-quality studies.

### Two-sample Mendelian randomization analysis

To further investigate the potential causal relationships between risk factors and PS, we conducted MR.

### GWAS data sources

Outcome data for PS were obtained from the FinnGen consortium (phenotype code: L12_PILONIDALCYST), including 474,291 individuals of European ancestry—comprising 3,784 cases and 470,507 controls. Genome-wide association study (GWAS) summary statistics for exposure traits were obtained from European-ancestry populations in the UK Biobank (UKB), IEU OpenGWAS, and other publicly available sources. This approach minimized the risk of sample overlap and population stratification.

### Selection of instrumental variables (SNPs)

The selection of SNPs as IVs followed the three core assumptions of MR: relevance, independence, and exclusion restriction (10). SNPs strongly associated with the exposure (*p* < 5 × 10⁻^8^) were initially selected. If an insufficient number of SNPs met this threshold, the significance level was relaxed to *p* < 5 × 10⁻^6^. Linkage disequilibrium (LD) pruning was performed to exclude correlated SNPs (*r*^2^ < 0.001, window size = 10,000 kb). Moreover, SNPs directly associated with the outcome (PS) at *p* < 5 × 10⁻^5^ were excluded to reduce the risk of reverse causation. F-statistics (F = *β*^2^/SE^2^) were calculated for all selected SNPs, with F > 10 considered indicative of sufficient instrument strength to minimize weak instrument bias (11). To further mitigate confounding, all SNPs were cross-referenced using LDlink (https://ldlink.nih.gov) to exclude those associated with known confounders (12). Sensitivity analyses were conducted to assess the robustness of the results.

### MR analysis

The primary method used to estimate causal effects was inverse-variance weighting (IVW), complemented by the weighted median and MR-Egger regression methods (13). Although the IVW method, which assumes that all IVs are valid, is the most commonly applied approach, it may be biased in the presence of horizontal pleiotropy (14). MR-Egger regression accounts for unbalanced horizontal pleiotropy and provides unbiased estimates under the InSIDE assumption (15). The weighted median method yields consistent estimates even when up to 50% of the total weight is derived from IVs (16). Heterogeneity among IVs was assessed using Cochran's *Q* test, with *p* < 0.05 indicating significant heterogeneity (5). The MR-Egger intercept was used to test for directional pleiotropy, with a *p*-value < 0.05 indicating its presence (15). When pleiotropy was detected, outlier SNPs were identified using the MR-PRESSO method and manually excluded before performing the MR analysis again to correct for potential bias (17). To further identify statistically significant associations, the *p*-values derived from the IVW analyses were adjusted using the Benjamini–Hochberg false discovery rate (FDR) method (18).

All MR analyses were conducted using R (version 4.3.3), primarily utilizing the *TwoSampleMR* (v0.6.0), *MendelianRandomization* (v0.8.0), and *MR-PRESSO* (v1.0) packages.

## Result

### Meta-analysis and systematic review

**Figure 1:**
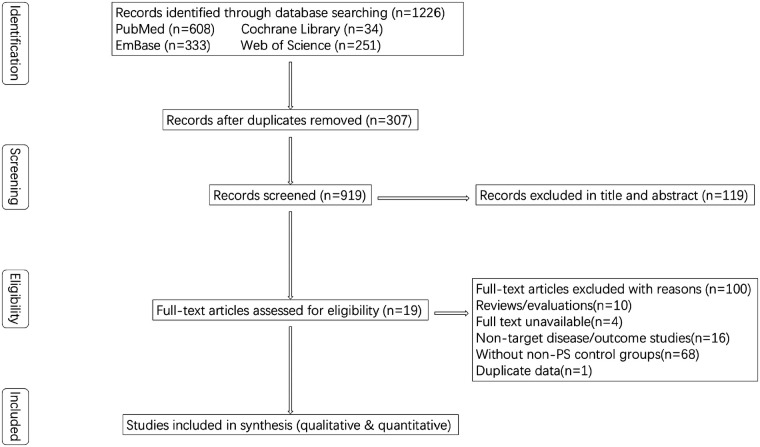
PRISMA flow diagram of study selection.

A total of 1,226 records were identified through database searches: 608 from PubMed, 34 from the Cochrane Library, 251 from Web of Science, and 333 from Embase. After removing duplicates, 919 unique studies remained. Following title and abstract screening, 119 full-text articles were assessed for eligibility. After full-text screening based on the predefined inclusion and exclusion criteria, a total of 100 articles were excluded, including reviews or evaluations (*n* = 10), studies without accessible full text (*n* = 4), studies involving non-target diseases or outcomes (*n* = 16), and studies lacking non-PS control groups (*n* = 68). The risk factor data reported by Bannura (19) were identical to those presented in another of his publications (33); therefore, this duplicate study was excluded from the meta-analysis. A full list of excluded studies and the corresponding reasons is provided in Supplementary Table S1. Ultimately, 19 studies met the inclusion and exclusion criteria (Table 1). Among these, eight studies were included in the narrative synthesis only, due to an insufficient number of comparable studies addressing the same risk factors, and were not eligible for meta-analysis. The study selection process is illustrated in Figure 1.

**Table 1:** Characteristics and quality assessment of included studies.

Reference	Year	Country	Age, mean ± SD (range)		Sample		Sex		Risk factors	NOS
			PS	Control	PS	Control	M	F		
Eryilmaz et al. (20)	2015	Turkey	24 (18–48) years	23 (18–45) years	30	30	60	0	a	8
Kanlioz et al. (21)	2021	Turkey	25.69 ± 5.19 years	25.57 ± 4.68 years	481	128	475	134	beghjkm	9
Ugurlu et al. (22)	2022	Turkey	NA	NA	11	149	0	160	s	9
Yildiz et al. (23)	2016	Turkey	187.93 ± 19.73 months	183.13 ± 16.92 months	42	40	39	43	cdefgj	8
Doll et al. (24)	2021	Germany	27.8 ± 10.5 years	38.8 ± 19.6 years	100	459	327	232	bku	9
Bolandparvaz et al. (25)	2012	Iran	25.1 (13–30) years	NA	99	101	150	50	efg	7
Harlak et al. (26)	2010	Turkey	22.49 ± 2.97 years	21.02 ± 1.73 years	587	2,780	3,367	0	bcdefgm	8
Çubukçu et al. (27)	2001	Turkey	27 (16–64) years	26 (17–61) years	419	213	520	112	bcd	8
Akinci et al. (28)	1999	Turkey	NA	NA	88	912	1,000	0	efghijq	8
Yigit et al. (29)	2023	Turkey	25.36 ± 7.07 (18–45) years	26.42 ± 5.83 (18–40) years	50	50	0	100	bdhst	9
Akinci et al. (30)	2009	Turkey	27.45 years	26.94 years	50	51	88	13	a	8
Faraj et al. (31)	2020	Iraq	NA	NA	94	95	80	109	ghkmor	9
Oetzmann von Sochaczewski et al. (32)	2024	Germany	Median 25 (IQR 14) years	Median 33 (IQR 33) years	76	340	226	190	bhp	7
Bannura et al. (33)	2007	Chile	25.3 ± 11.9 years	27.79 years	74	62	70	66	bm	7
Ekici and Moray (34)	2021	Turkey	23.9 ± 4.5 (18–43) years	27.1 ± 6.2 (18–41) years	45	100	145	0	bcdegmnq	9
Doll et al. (35)	2017	Germany	NA	NA	17	217	116	118	p	7
Özkan et al. (36)	2014	Turkey	23 (15–45) years	21 years	39	39	52	26	t	8
Atay et al. (37)	2022	Turkey	28.26 (21–39) years	28.26 (21–39) years	50	50	43	7	l	8
Maak et al. (38)	2025	Germany	37.7 ± 15.5 years	37.7 ± 15.5 years	95	105	125	75	a	6

a: natal cleft depth/sacrococcygeal angle; b: BMI; c: overweight; d: obesity; e: prolonged sitting; f: bathing frequency; g: family history; h: smoking; i: alcohol consumption; j: skin color; k: oily skin/hyperhidrosis; l: cutaneous collagen; m: hair density; n: pili multigemini, curly hair; o: hair color; p: hair strength; q: folliculitis, acne; r: diabetes mellitus; s: PCOS; t: sex hormones, serum lipid levels.

For outcomes with high heterogeneity, leave-one-out sensitivity analyses were performed, demonstrating that all pooled estimates remained robust (Supplementary Figures S2–S9). Although fewer than 10 studies were included for each risk factor (*N* ≤ 8), Egger's test and funnel plot analyses were still performed to assess potential small-study effects. However, given the limited number of included studies, the statistical power of these assessments was low, and the results should be interpreted with caution. Funnel plots, GRADE evidence profiles, and leave-one-out plots are provided in Supplementary Figures S2–S20.

### Body mass index

**Figure 2:**
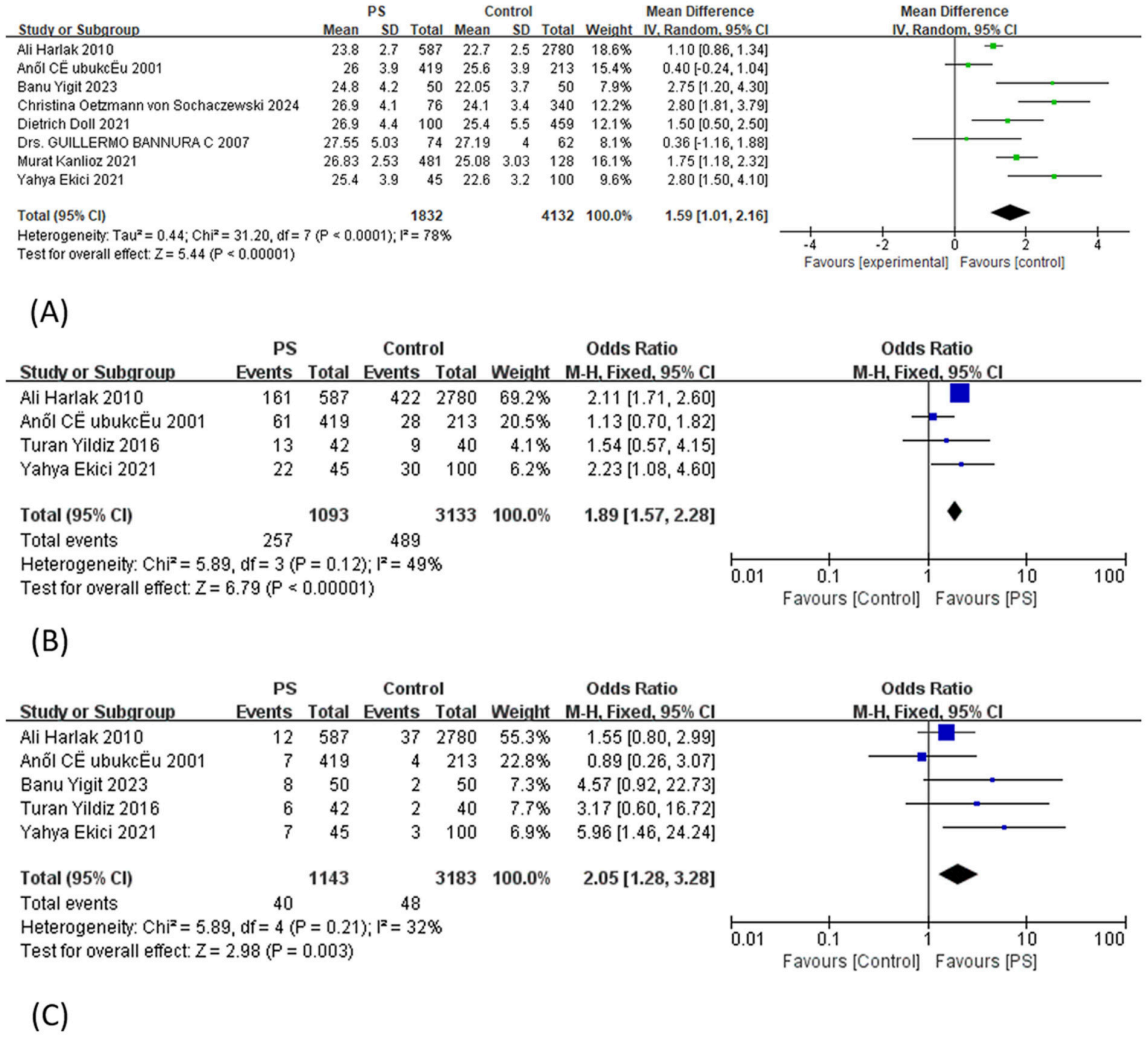
Forest plot from the meta-analysis of BMI (**A**), overweight (**B**), and obesity (**C**) in relation to pilonidal sinus disease risk.

Moderate-quality evidence indicated that across the eight included studies, body mass index (BMI) was significantly higher in the PS group compared with controls (SD = 1.59), although substantial heterogeneity was observed. Subgroup analyses were conducted based on the inclusion of minors (adults vs. included minors), mean age (<26.5 vs. >26.5 years), study population (conscript vs. general population), sex ratio (M/F > 3 vs. M/F < 3), and publication year (by 2010 vs. after 2010) (Supplementary Figure S1). In addition, meta-regression analyses were performed (Table 2). None of these individual factors explained the observed heterogeneity (all *P* > 0.05). However, when publication year and study population were jointly included in a multivariable meta-regression model, the model accounted for 100% of the between-study heterogeneity (*R*^2^ = 100%), with statistically significant contributions from both variables (*P* = 0.013 and 0.007, respectively; Table 2). Publication year reflects methodological evolution and improvements in study design, whereas study population type captures differences in physical characteristics and lifestyle patterns among sampled individuals. The combined influence of these two factors effectively explained the systematic variation across studies, leaving a residual heterogeneity of only 10.32%. Funnel plot symmetry and Egger's test showed no evidence of publication bias (*P* = 0.296). Further analysis revealed that both overweight (moderate-quality evidence) and obesity (high-quality evidence) were associated with an increased risk of PS, with ORs of 1.89 and 2.05, respectively (Figure 2). No publication bias was detected for either factor (P_Egger = 0.513 and 0.296, respectively). In the analysis of overweight, exclusion of the study by Harlak et al. (26) led to a notable reduction in heterogeneity (*I*^2^ decreased from 49% to 17%), while the pooled effect size remained largely unchanged, indicating robust results.

**Table 2:** Meta-regression analysis of potential sources of heterogeneity in BMI differences between PS and control groups.

Subgroup	R^2^ (%)	I^2^_res (%)	*P*
Included minors (yes vs. no)	21.55	79.85	0.147
Mean age (<26.5 vs. > 26.5 years)	−10.07	82.3	0.523
Study population (conscript vs. general)	45.87	71.63	0.088
Sex ratio (M/F > 3 vs. M/F < 3)	−22.16	84.94	0.920
Publication year (by 2010 vs. after 2010)	46.95	77.64	0.051
Study population + publication year	100	10.32	0.0072
Study population			0.007
Publication year			0.013

### Sedentary behavior

Multiple studies consistently indicated that sedentary behavior is significantly associated with an increased risk of PS. Kanlioz et al. (21) and Ekici and Moray (34) reported that the average daily sitting time was significantly longer in the PS group than in the control group (8.89 ± 2.14 vs. 7.41 ± 2.02 h; 6.2 ± 1.5 vs. 3.8 ± 1.7 h, respectively). Yildiz et al. (23), Harlak et al. (26), and Ekici and Moray (34) all suggested that prolonged sitting (≥6 h/day) increases the risk of PS, whereas Bolandparvaz et al. (25) identified sitting for more than 4 h/day as a potential risk factor. Akinci et al. (28) further found that drivers were significantly more common in the PS group than in controls (66% vs. 34%, *p* < 0.0001). Collectively, these findings strongly support sedentary behavior as a major behavioral risk factor for PS. In addition, a study by Ekici and Moray (34) reported a significant association between a reclining sitting posture and PS, while Faraj et al. (31) identified hard seating surfaces as an independent risk factor for PS among students.

### Family history

**Figure 3:**
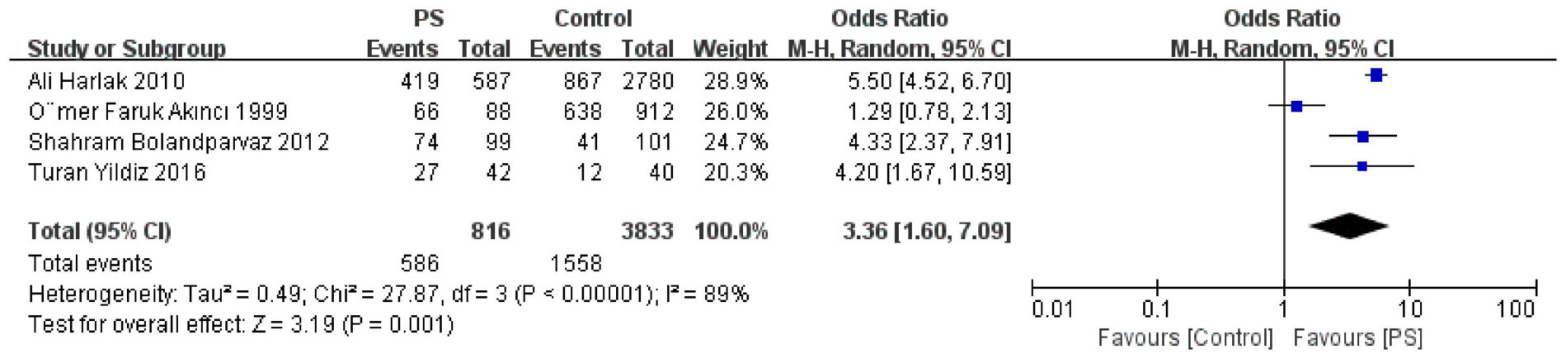
Meta-analysis forest plot of bathing frequency of ≤2 times per week and the risk of pilonidal sinus disease.

**Figure 4:**
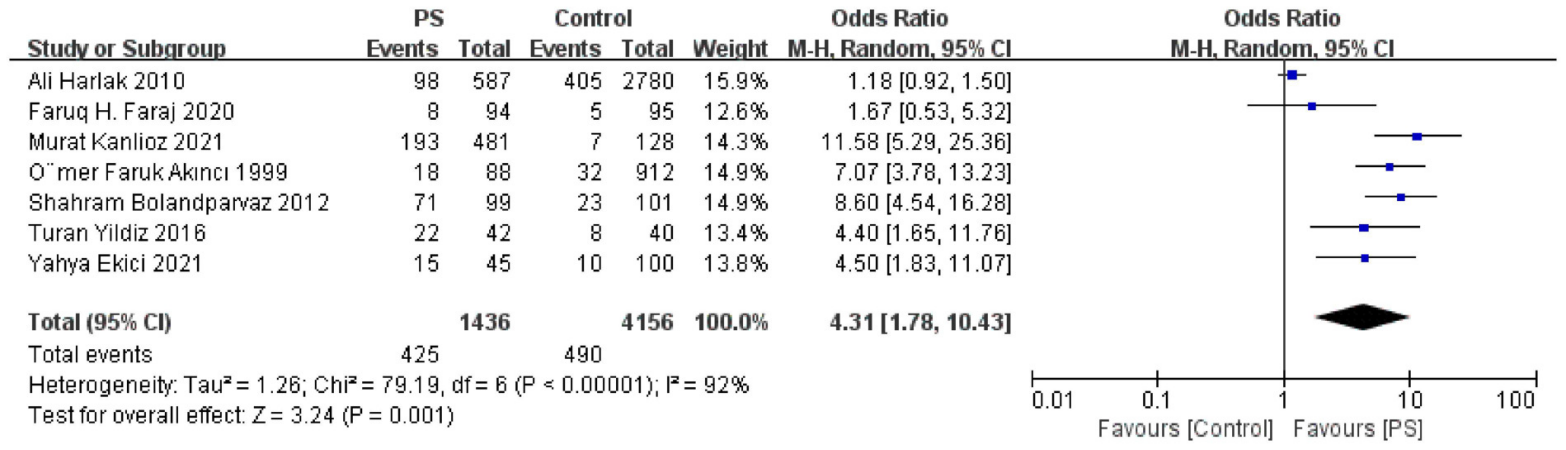
Meta-analysis forest plot of family history and the risk of pilonidal sinus disease.

A total of seven studies reported data on family history. The prevalence of positive family history was 29.6% in the PS group compared with 11.8% in the control group (Figure 3). Meta-analysis demonstrated that a positive family history was significantly associated with increased PS risk (OR = 4.31, Figure 4). Egger's test indicated potential publication bias (*P* = 0.043). After applying the trim-and-fill method and imputing seven hypothetical studies, the pooled effect estimate remained significant (OR = 4.31, *P* < 0.001; Supplementary Figure S12B), further supporting the robustness of the association.

### Hygiene

Four studies reported that bathing ≤2 times per week was significantly associated with a higher risk of PS (OR = 3.36; Figure 3). The quality of evidence was rated as high. These findings suggest that poor hygiene is a risk factor for PS. Sensitivity analysis indicated the results were robust, and no publication bias was detected (Egger's test *P* = 0.436).

### Hair

**Figure 5:**
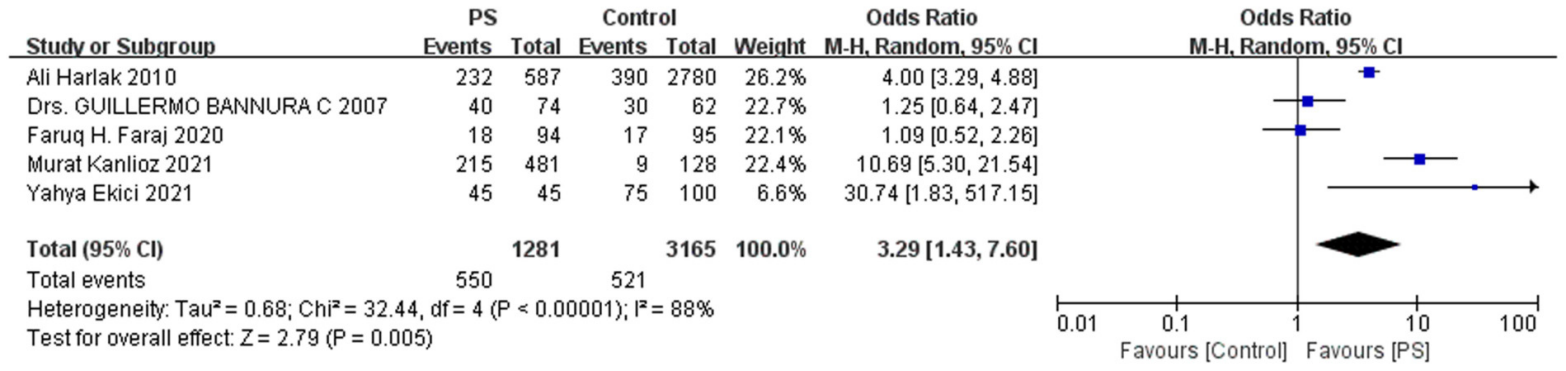
Meta-analysis forest plot of dense body hair and the risk of pilonidal sinus disease.

High-quality evidence supports that dense body hair is a significant risk factor for PS (OR = 3.29; Figure 5), with no indication of publication bias. In addition, a study by Ekici and Moray (34) reported that all PS cases exhibited pili multigemini (100% vs. 47% in controls), and 28% had curly hair, suggesting that both pili multigemini and hair curvature may be additional risk factors for PS. Faraj et al. (29) compared hair color between cases and controls, and found no statistically significant difference. Oetzmann von Sochaczewski et al. (32) reported that axial hair strength was significantly higher in the PS group (1.44 vs. 1.15) and identified it as an independent risk factor (OR = 1.68, *P* = 0.002). Doll et al. (35) proposed that the hair contained within PS sinus tracts is most likely derived from occipital scalp hair.

### Smoking and drinking

**Figure 6:**
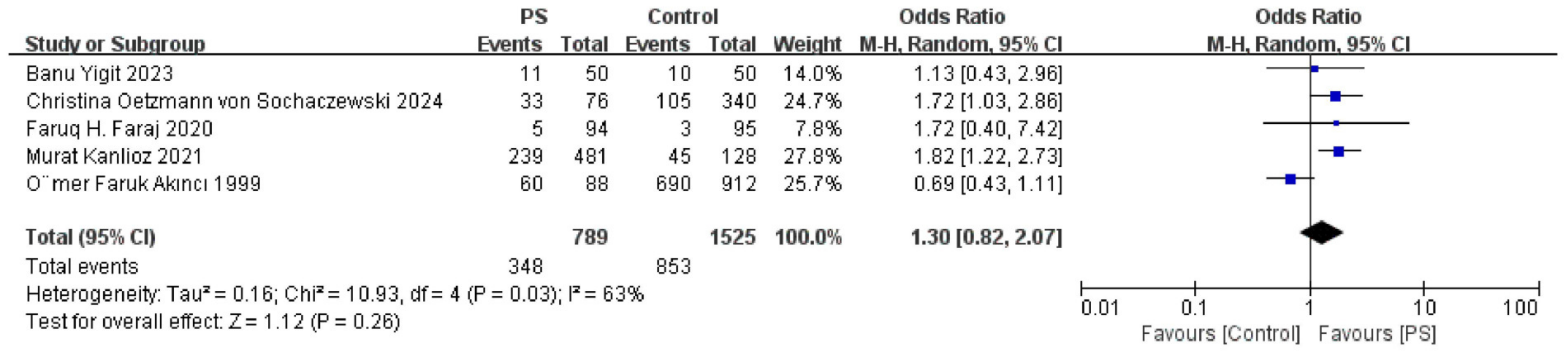
Meta-analysis forest plot of smoking and the risk of pilonidal sinus disease.

Five included studies reported data on smoking history, involving 789 patients with PS, of whom 44.1% had a history of smoking. Among 1,525 controls, 55.9% were smokers. The difference was not statistically significant (Figure 6), and no publication bias was detected (*P* = 0.945). Akinci et al. (28) reported alcohol consumption rates of 30% in the PS group and 24% in the control group, with no significant difference. Therefore, smoking and alcohol consumption are unlikely to be independent risk factors for PS.

### Skin

Two studies (23, 28) compared skin color between PS patients and controls and found no statistically significant differences. One study (21) suggested a potential association (Brown: OR = 1.65, *P* = 0.029); however, the classification of skin color may reflect different ethnic backgrounds, and given the variability in body hair density across ethnicities, skin color may represent an indirect rather than causal factor.

Regarding skin properties, Faraj (31) found no significant association between sweating and PS, while Doll et al. (24) reported that sweating might have a protective effect (11.9 vs. 18.6 μL, *P* = 0.005). In contrast, Kanlioz et al. (21) suggested that oily skin may be a risk factor for PS (OR = 3.27). Moreover, a study by Atay et al. (37) found that the total collagen content (2.92 ± 0.82 vs. 3.54 ± 0.78, *P* < 0.001) and the type I/III collagen ratio (1.08 ± 0.37 vs. 1.61 ± 0.44, *P* < 0.001) in midline skin of PS patients were significantly lower than in adjacent healthy skin. Moreover, obese patients had lower midline collagen levels compared to non-obese individuals, suggesting that reduced collagen content may compromise skin integrity and facilitate hair penetration.

### Gluteal fissure depth

Some studies (30) reported that the depth of gluteal fissure was a risk factor (27.06 vs. 21.07 mm). However, other studies failed to demonstrate a significant association (*P* = 0.833) (38). Eryilmaz et al. (20) also found no significant association between PS and sacrococcygeal angle—a surrogate measure of natal cleft depth (smaller angles indicate deeper clefts)—reporting similar values in cases and controls (37.30 ± 14.50 vs. 36.86 ± 10.23).

### Sex hormones, blood lipids

One study (29) measured serum levels of sex hormones—including estrogen, progesterone, testosterone, prolactin, luteinizing hormone (LH), and follicle-stimulating hormone (FSH)—in female PS patients and controls, and found no significant associations. Another study (36) reported that prolactin levels were significantly higher in female PS patients compared to female controls. Yigit et al. (29) observed that the mean high-density lipoprotein (HDL) level was lower in the PS group (48.7 ± 10.33) than in controls (55.1 ± 10.88), whereas no statistically significant differences were found in triglycerides, low-density lipoprotein (LDL), or total cholesterol levels.

### Motion

Doll et al. (24) found no significant association between physical activity frequency and the risk of PS.

### Related diseases

Studies (28, 34) reported that 25% and 80% of patients with PS, respectively, had coexisting skin conditions such as folliculitis and acne, both higher than in control groups. Yigit et al. (29) found increased right ovarian volume in female PS patients, suggesting features of PCOS. Ugurlu et al. (22) further confirmed PCOS as an independent risk factor for PS (OR = 17.29, *P* = 0.004). In addition, one study (31) reported diabetes prevalence rates of 2.1% (2/95) in the PS group and 1.1% (1/95) in the control group, with no statistically significant difference (*P* = 0.554). The lack of significance may reflect limited sample size, potentially masking a true association.

### Two-sample MR analysis

**Figure 7:**
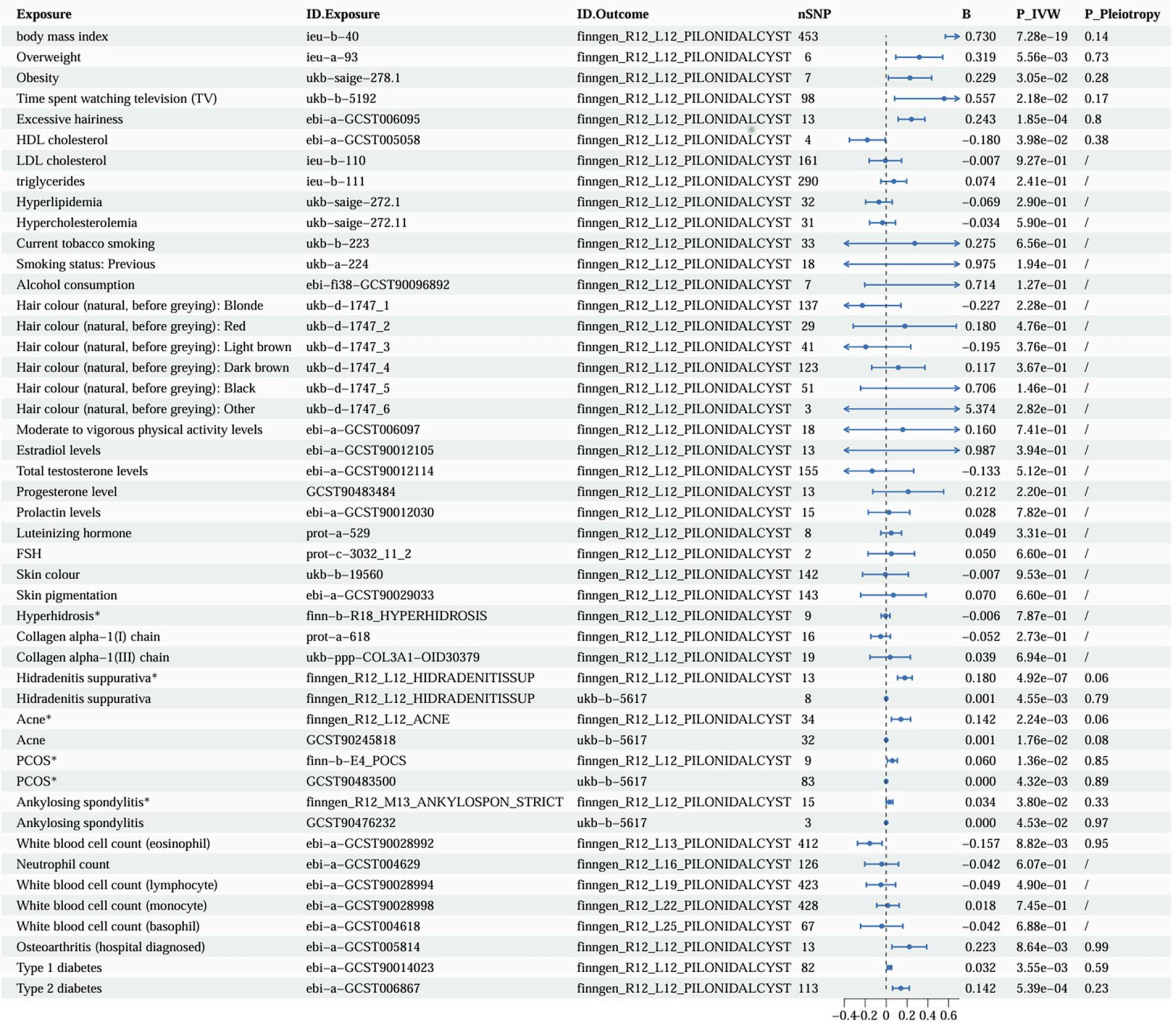
Forest plot of main two-sample Mendelian randomization (IVW) results and pleiotropy tests for significant associations.

The GWAS identifiers for all exposure variables and the main MR results are presented in Table 3 and Figure 7, while details of excluded confounding SNPs and outlier SNPs are provided in Supplementary Table S2. Additional MR-related data are included in Supplementary Data Sheet S2. None of the results required exclusion based on sensitivity analyses. After FDR correction, hidradenitis suppurativa (HS), osteoarthritis, BMI, overweight, excessive hairiness, type 1 diabetes (T1D), type 2 diabetes (T2D), and white blood cell count (eosinophils) remained statistically significant. In contrast, the associations of acne, PCOS, obesity, time spent watching television (TV), HDL cholesterol, and ankylosing spondylitis (AS) with PS were no longer significant after correction. Nevertheless, these latter factors exhibited nominal significance prior to adjustment, suggesting the possibility of underlying causal relationships that warrant further investigation.

**Table 3:** Main results of two-sample Mendelian randomization analysis (IVW) for all exposures and heterogeneity/pleiotropy tests for significant associations.

id.exposure	exposure	id.outcome	nsnp	b	p_ivw	p_fdr	p_pleiotropy
finngen_R12_L12_HIDRADENITISSUP^a^	Hidradenitis suppurativa	finngen_R12_L12_PILONIDALCYST	13	0.18	4.92 × 10^−7^	1.16 × 10^−5^	0.06
finngen_R12_L12_HIDRADENITISSUP	Hidradenitis suppurativa	ukb-b-5617	8	0.001	4.55 × 10^−3^	2.67 × 10^−2^	0.79
finngen_R12_L12_ACNE^a^	Acne	finngen_R12_L12_PILONIDALCYST	34	0.14	2.24 × 10^−3^	2.11 × 10^−2^	0.06
GCST90245818	Acne	ukb-b-5617	32	0	1.76 × 10^−2^	6.36 × 10^−2^	0.08
finn-b-E4_POCS^a^	PCOS	finngen_R12_L12_PILONIDALCYST	9	0.06	1.36 × 10^−2^	5.33 × 10^−2^	0.85
GCST90483500^a^	PCOS	ukb-b-5617	83	0	4.32 × 10^−3^	2.67 × 10^−2^	0.89
ebi-a-GCST005814	Osteoarthritis (hospital diagnosed)	finngen_R12_L12_PILONIDALCYST	13	0.22	8.64 × 10^−3^	3.77 × 10^−2^	0.99
ieu-b-40	Body mass index	finngen_R12_L12_PILONIDALCYST	453	0.73	7.28 × 10^−19^	3.42 × 10^−17^	0.14
ieu-a-93	Overweight	finngen_R12_L12_PILONIDALCYST	6	0.32	5.56 × 10^−3^	2.90 × 10^−2^	0.73
ukb-saige-278.1	Obesity	finngen_R12_L12_PILONIDALCYST	7	0.23	3.05 × 10^−2^	9.56 × 10^−2^	0.28
ebi-a-GCST006095	Excessive hairiness	finngen_R12_L12_PILONIDALCYST	13	0.24	1.85 × 10^−4^	2.90 × 10^−3^	0.80
ukb-b-5192	Time spent watching television (TV)	finngen_R12_L12_PILONIDALCYST	98	0.56	2.18 × 10^−2^	7.32 × 10^−2^	0.17
ebi-a-GCST005058	HDL cholesterol	finngen_R12_L12_PILONIDALCYST	4	−0.18	3.98 × 10^−2^	1.10 × 10^−1^	0.38
ebi-a-GCST90014023	Type 1 diabetes	finngen_R12_L12_PILONIDALCYST	82	0.03	3.55 × 10^−3^	2.67 × 10^−^2	0.59
finngen_R12_M13_ANKYLOSPON_STRICT^a^	Ankylosing spondylitis, strict definition	finngen_R12_L12_PILONIDALCYST	15	0.03	3.80 × 10^−2^	1.10 × 10^−1^	0.33
GCST90476232	Ankylosing spondylitis	ukb-b-5617	3	0	4.53 × 10^−2^	1.18 × 10^−1^	0.97
ebi-a-GCST006867	Type 2 diabetes	finngen_R12_L12_PILONIDALCYST	113	0.14	5.39 × 10^−4^	6.33 × 10^−3^	0.23
ebi-a-GCST90028992	White blood cell count (eosinophil)	finngen_R12_L13_PILONIDALCYST	412	−0.16	8.82 × 10^−3^	3.77 × 10^−2^	0.95
ebi-a-GCST004629	Neutrophil count	finngen_R12_L16_PILONIDALCYST	126	−0.04	6.07 × 10^−1^	7.92 × 10^−1^	
ebi-a-GCST90028994	White blood cell count (lymphocyte)	finngen_R12_L19_PILONIDALCYST	423	−0.05	4.90 × 10^−1^	6.98 × 10^−1^	
ebi-a-GCST90028998	White blood cell count (monocyte)	finngen_R12_L22_PILONIDALCYST	428	0.02	7.45 × 10^−1^	8.14 × 10^−1^	
ebi-a-GCST004618	White blood cell count (basophil)	finngen_R12_L25_PILONIDALCYST	67	−0.04	6.88 × 10^−1^	7.96 × 10^−1^	
ebi-a-GCST90029033	Skin pigmentation	finngen_R12_L12_PILONIDALCYST	143	0.07	6.60 × 10^−1^	7.95 × 10^−1^	
ebi-a-GCST006097	Moderate to vigorous physical activity levels	finngen_R12_L12_PILONIDALCYST	18	0.16	7.41 × 10^−1^	8.14 × 10^−1^	
ebi-a-GCST90012105	Estradiol levels	finngen_R12_L12_PILONIDALCYST	13	0.99	3.94 × 10^−1^	5.97 × 10^−1^	
ebi-a-GCST90012114	Total testosterone levels	finngen_R12_L12_PILONIDALCYST	155	−0.13	5.12 × 10^−1^	7.08 × 10^−1^	
ebi-fi38-GCST90096892	Alcohol consumption	finngen_R12_L12_PILONIDALCYST	7	0.71	1.27 × 10^−1^	3.14 × 10^−1^	
ieu-b-110	LDL cholesterol	finngen_R12_L12_PILONIDALCYST	161	−0.01	9.27 × 10^−1^	9.47 × 10^−1^	
ieu-b-111	triglycerides	finngen_R12_L12_PILONIDALCYST	290	0.07	2.41 × 10^−1^	4.72 × 10^−1^	
ukb-a-224	Smoking status: Previous	finngen_R12_L12_PILONIDALCYST	18	0.97	1.94 × 10^−1^	4.34 × 10^−1^	
ukb-b-19560	Skin color	finngen_R12_L12_PILONIDALCYST	142	−0.01	9.53 × 10^−1^	9.53 × 10^−1^	
ukb-b-223	Current tobacco smoking	finngen_R12_L12_PILONIDALCYST	33	0.28	6.56 × 10^−1^	7.95 × 10^−1^	
ukb-d-1747_1	Hair color (natural, before graying): blonde	finngen_R12_L12_PILONIDALCYST	137	−0.23	2.28 × 10^−1^	4.66 × 10^−1^	
ukb-d-1747_2	Hair color (natural, before graying): Red	finngen_R12_L12_PILONIDALCYST	29	0.18	4.76 × 10^−1^	6.98 × 10^−1^	
ukb-d-1747_3	Hair color (natural, before graying): light brown	finngen_R12_L12_PILONIDALCYST	41	−0.2	3.76 × 10^−1^	5.89 × 10^−1^	
ukb-d-1747_4	Hair color (natural, before graying): dark brown	finngen_R12_L12_PILONIDALCYST	123	0.12	3.67 × 10^−1^	5.89 × 10^−1^	
ukb-d-1747_5	Hair color (natural, before graying): black	finngen_R12_L12_PILONIDALCYST	51	0.71	1.46 × 10^−1^	3.43 × 10^−1^	
ukb-d-1747_6	Hair color (natural, before graying): other	finngen_R12_L12_PILONIDALCYST	3	5.37	2.82 × 10^−1^	5.05 × 10^−1^	
ukb-saige-272.1	Hyperlipidemia	finngen_R12_L12_PILONIDALCYST	32	−0.07	2.90 × 10^−1^	5.05 × 10^−1^	
ukb-saige-272.11	Hypercholesterolemia	finngen_R12_L12_PILONIDALCYST	31	−0.03	5.90 × 10^−1^	7.92 × 10^−1^	
GCST90483484	Progesterone level	finngen_R12_L12_PILONIDALCYST	13	0.21	2.20 × 10^−1^	4.66 × 10^−1^	
finn-b-R18_HYPERHIDROSIS^a^	Hyperhidrosis	finngen_R12_L12_PILONIDALCYST	9	−0.01	7.87 × 10^−1^	8.22 × 10^−1^	
ebi-a-GCST90012030	Prolactin levels	finngen_R12_L12_PILONIDALCYST	15	0.03	7.82 × 10^−1^	8.22 × 10^−1^	
prot-a-529	Luteinizing hormone	finngen_R12_L12_PILONIDALCYST	8	0.05	3.31 × 10^−1^	5.56 × 10^−1^	
prot-a-618	Collagen alpha-1(I) chain	finngen_R12_L12_PILONIDALCYST	16	−0.05	2.73 × 10^−1^	5.05 × 10^−1^	
prot-c-3032_11_2	FSH	finngen_R12_L12_PILONIDALCYST	2	0.05	6.60 × 10^−1^	7.95 × 10^−1^	
ukb-ppp-COL3A1-OID30379	Collagen alpha-1(III) chain	finngen_R12_L12_PILONIDALCYST	19	0.04	6.94 × 10^−1^	7.96 × 10^−1^	

a Partial sample overlap.

Consistent with the meta-analysis findings, the MR analysis demonstrated a positive association between BMI and PS risk (*β* = 0.24), indicating that both overweight and obesity may act as contributing factors. Due to the unavailability of GWAS data for daily sedentary time, television watching time was used as a proxy indicator. The results indirectly suggest that sedentary behavior may promote the development of PS. Hair density was also confirmed as a risk factor. In contrast, higher HDL levels appeared to have a protective effect (*β* = –0.18), consistent with previous observational research (29). Furthermore, the MR analysis did not support a causal relationship between PS and the following factors: smoking, alcohol consumption, hair color, physical activity, six sex hormone levels, total cholesterol, triglycerides, LDL, and hyperlipidemia.

Associations of skin color and oily skin/hyperhidrosis with PS have been inconsistent across observational studies, whereas the MR analysis did not support any direct genetic causal relationship between these phenotypes and PS. Notably, the GWAS for hyperhidrosis was also derived from FinnGen, with an estimated sample overlap of approximately 37% with the PS dataset; however, since the MR results were not statistically significant, further replication was not pursued. In addition, Atay et al. (37) suggested that reduced collagen content in the natal cleft may facilitate hair penetration into the skin. The MR analysis did not identify significant genetic associations between PS risk and key structural skin collagens—Collagen_alpha-1(I)_chain and Collagen_alpha-1(III)_chain. Nevertheless, the IVs used for these exposures were based on plasma protein protein quantitative trait loci (pQTLs), which may not adequately capture the local dermal collagen architecture or biomechanical properties of the sacrococcygeal region.

Based on MR analyses using FinnGen data, individuals with HS, acne, PCOS, and AS exhibited an increased risk of PS (*β* = 0.18, 0.14, 0.06, and 0.03, respectively). Because both exposure and outcome data were derived from the same database, substantial sample overlap was present, with estimated overlap proportions of 44.6%, 45%, 56.9%, and 95%, respectively. To minimize potential bias introduced by sample overlap, we further performed two-sample MR validation using independent datasets—Acne (GCST90245818), HS (L12_HIDRADENITISSUP), PCOS (GCST90483500), and AS (GCST90476232)—with PS outcome data obtained from the UK Biobank (ukb-b-5617). In these validation analyses, the sample overlap between Acne, HS, and AS with the PS dataset was 0%. The results demonstrated consistent effect directions with statistically significant *p*-values, supporting the robustness of the original findings (Figure 7). However, the PCOS dataset (GCST90483500) included approximately 229,794 UKB participants, resulting in a potential overlap of up to 49.6% with the PS outcome dataset (ukb-b-5617). Despite this overlap, when considered alongside evidence from the systematic review, a potential causal relationship between PCOS and PS cannot be excluded.

In addition, MR analyses indicated that elevated peripheral eosinophil counts may exert a protective effect against PS (*β* = −0.16). In contrast, no significant causal associations were observed for neutrophils, basophils, monocytes, or lymphocytes. Conversely, individuals with osteoarthritis (*β* = 0.22) or diabetes (type 1: *β* = 0.03; type 2: *β* = 0.14) appeared to have an increased risk of developing PS, suggesting that chronic inflammatory states or metabolic dysregulation may contribute to its pathogenesis.

## Discussion

This study systematically examined the risk factors for PS using both meta-analysis and MR approaches. The findings suggest that multiple factors contribute to the pathogenesis of PS. To avoid ambiguity arising from nominally significant associations, we emphasize that only HS, osteoarthritis, BMI, overweight, excessive hairiness, T1D, T2D, and eosinophil count remained statistically significant after FDR correction. Importantly, MR studies identify potential causal relationships driven by genetic predisposition, rather than short-term modifiable risks. Therefore, the interpretation of causality should be confined to the genetic level, and further validation through prospective studies and experimental research remains necessary.

The results indicated that increased BMI, sedentary behavior, dense body hair, poor hygiene, and a positive family history are established pathogenic factors for PS. In obese individuals, a deeper and narrower natal cleft may lead to sweat accumulation and inadequate ventilation (32), while prolonged sitting may cause local pressure and friction, resulting in follicular damage and subsequent inflammation (39, 40). Reclining postures and hard seating surfaces further exacerbate localized mechanical stress and friction, thereby promoting PS formation (31, 33). Evidence also suggests that different hair characteristics contribute to PS through distinct mechanisms. First, individuals with dense body hair have a higher likelihood of hair penetrating the skin and triggering an inflammatory response (21). Second, curly hair, which tangles and sheds less readily, is more prone to penetrate the skin under pressure and friction in the sacrococcygeal area, thereby facilitating sinus tract formation (33). Third, small-scale mechanistic studies have demonstrated that hair from PS patients exhibits greater axial strength, suggesting that increased hair shaft rigidity may enhance its ability to penetrate the skin, elevating disease risk. This observation aligns with the disease's onset patterns related to sex and adolescence (32, 35), and provides theoretical support for the hypothesis proposed by Doll et al. (41) that scalp hair is the primary source of hair found in PS. It should be emphasized that evidence regarding hair curliness and axial hair strength derives mainly from mechanistic studies with limited sample sizes. These findings may be more appropriate for explaining underlying biological mechanisms rather than serving as definitive epidemiological evidence. Poor hygiene practices may compromise the skin barrier and increase susceptibility to infection (42). However, the bathing frequency data (≤2 times per week) reported in the four included studies were all based on patient self-report, which is inherently subjective. Moreover, these studies did not control for potential confounders such as occupation, environmental climate, or living conditions; therefore, the association between bathing frequency and PS should be interpreted with caution. Moreover, individuals with a positive family history exhibited a markedly higher risk of PS, suggesting a possible hereditary predisposition.

In contrast, smoking and alcohol consumption were not identified as significant risk factors in this study. A meta-analysis of five studies found no significant association between smoking and PS, a finding further supported by the MR analysis, which excluded a genetic causal relationship. Evidence supporting alcohol consumption as a risk factor was similarly limited. Individual studies reported non-significant results, and the MR analysis did not detect any causal signal, suggesting a weak association between alcohol use and PS. We speculate that smoking and alcohol primarily affect systemic metabolism and have minimal influence on local skin–hair dynamics or pressure in the natal cleft area. Similarly, hair color and physical activity were not found to be causally related to PS. Although clinical studies have reported inconsistent findings regarding skin color and hyperhidrosis, the MR analysis did not support a genetic causal relationship, possibly due to confounding in observational studies. For example, the skin color categories used by Kanlioz et al. (21) may reflect different ethnic groups, among which body hair density varies, suggesting that skin color may act as an indirect proxy rather than a direct risk factor. Moreover, the MR analysis did not reveal a causal association between skin collagen and PS. This may be due to the use of plasma protein pQTLs as IVs, which likely fail to accurately represent the local dermal collagen composition and biomechanical properties of the sacrococcygeal region.

At the metabolic level, the MR analysis revealed that higher HDL levels were inversely associated with PS risk. Although this association did not remain statistically significant after FDR correction, it still carries potential biological relevance and is consistent with findings from the systematic review. Elevated HDL levels may reduce the production of inflammatory markers, thereby exerting anti-inflammatory and protective effects that lower the likelihood of PS development (43). In contrast, no significant associations were observed between PS and six major sex hormone levels or other lipid parameters apart from HDL. In addition, studies using skin infection models have demonstrated that eosinophil-derived IL-4 and IL-13 can sustain M2-like dermal resident macrophages and maintain skin barrier homeostasis, thereby limiting inflammation and promoting tissue repair (44). This mechanism provides a plausible biological explanation for the observation that elevated eosinophil counts may reduce the risk of chronic sinus tract formation following hair penetration. Nonetheless, this hypothesis requires further experimental validation in future research.

Notably, this study expands the spectrum of diseases associated with PS and highlights its overlapping pathological features with multiple systemic conditions (45). The MR analysis revealed a genetic causal relationship between PS and follicular occlusion-related skin diseases such as HS and acne, suggesting that they may share common pathogenic pathways, including follicular hyperkeratosis, chronic inflammation, and microbiome dysbiosis (46). These findings not only enhance our understanding of the skin-originated mechanisms underlying PS but also provide a conceptual framework for its potential inclusion within the broader category of “follicular occlusion syndromes.” Importantly, diabetes was identified as a potential genetic risk factor for PS. Dysregulated glucose metabolism may contribute to PS development by impairing endothelial cell function in local tissues, leading to angiogenesis dysfunction and inadequate blood perfusion (47), or by increasing oxidative stress and systemic inflammation (48), thereby exacerbating tissue damage. In addition, patients with PCOS exhibited increased susceptibility to PS, indicating that endocrine dysregulation and insulin resistance may play critical roles in the pathogenesis of PS in women. The association between PS and osteoarticular conditions was also evident in our study. AS, a chronic autoimmune disorder primarily affecting the sacroiliac joints (49), showed a positive association with PS in the MR analysis. This finding is consistent with the study by Soy et al. (50), who observed a significantly higher prevalence of sacrococcygeal PS among AS patients compared to controls, suggesting a potential link between the two conditions. We speculate that inflammatory arthropathies may contribute to PS by altering the soft tissue structure or immune microenvironment in the sacrococcygeal region (51). Collectively, these findings suggest that PS may not be a purely localized skin disorder, but rather a multifactorial condition influenced by the interplay of dermatological, metabolic, endocrine, and immunological systems.

It should be noted that this study has several limitations. Some MR traits had insufficient statistical power; for example, using “Time spent watching television” as a proxy for sedentary behavior may not adequately capture other relevant contexts such as office work or driving. This variable is also influenced by lifestyle and socioeconomic factors, which may introduce residual confounding and potentially underestimate the overall effect of sedentary behavior on PS. The MR results for PCOS were limited by sample overlap; although the F-statistics for the IVs exceeded 10, bias may still have been introduced. In addition, several exposures lost statistical significance after FDR correction, suggesting that these associations may be constrained by limited statistical power. Moreover, the MR data were derived exclusively from European populations, and therefore the findings may not be generalizable to other regions or ethnic groups. For the systematic review and meta-analysis, the included studies varied in diagnostic criteria and in the methods used to assess risk factors. In addition, more than half of the studies were conducted in Turkey, which may introduce regional bias and limit the generalizability of the findings. Accordingly, larger, multicenter, and prospective studies are needed to further validate these conclusions.

## Conclusion

This study highlights that PS, as a chronic inflammatory disease of the sacrococcygeal region, may involve the interplay of multiple systemic conditions, revealing potential links between PS and various comorbidities. These findings provide a new perspective for future interdisciplinary research spanning dermatology, endocrinology, immunology, and related fields. The results also aid clinicians in identifying high-risk populations for PS and suggest that lifestyle interventions—such as weight management, reducing prolonged sitting, and improving local hygiene—may help lower disease risk. In addition, individuals with a family history or characteristic hair traits may benefit from targeted preventive measures, such as hair removal. For patients with diabetes, HS, acne, PCOS, or osteoarthritis, heightened vigilance and early screening are warranted to facilitate timely detection and early intervention.

## Data Availability

The original contributions presented in the study are included in the article/Supplementary Material, further inquiries can be directed to the corresponding author.
