# Supplementary material for: Risk factors for sacrococcygeal pilonidal sinus: a systematic review and meta-analysis supplemented by genetic causal assessment
Source: Front Surg. 2026 Jan 7;12:1718589. doi: 10.3389/fsurg.2025.1718589 (PMC12819706; doi:10.3389/fsurg.2025.1718589)

**Search Strategy**

**Databases searched:** PubMed, Embase, Web of Science, and the Cochrane Library. No separate grey-literature databases were searched.

**Final search date:** From database inception to June 23, 2025.

**Language restrictions:** None applied.

**Search process:** Searches were independently conducted and cross-checked by two reviewers (LX and PY).

**Search strings:** The search combined Medical Subject Headings (MeSH) terms and free-text keywords, including *“Pilonidal Sinus”* and *“risk factors.”* The detailed search strings for each database are provided below.

**PUBMED**

("Risk Factors"[Mesh]) OR (((((((((((((((((Risk Determinants) OR (Predisposing Factors)) OR (Contributing Factors)) OR (Causative Factors)) OR (Risk Contributors)) OR (Vulnerability Factors)) OR (dangerous factor)) OR (influencing factor)) OR (Determinants)) OR (predispose)) OR (Population at Risk)) OR (Risk Score)) OR (Risk Factor Score)) OR (Health Correlates)) OR (Score, Risk)) OR (Factor, Risk)) OR (Precipitating Factors)) 15,910,161

("Pilonidal Sinus"[Mesh]) OR ((((((((Sacrococcygeal Pilonidal Sinus) OR (Pilonidal Cyst)) OR (Pilonidal Cysts)) OR (Pilonidal Disease)) OR (Pilonidal Abscess)) OR (Sinus, Pilonidal)) OR (Cyst, Pilonidal)) OR (Cysts, Pilonidal)) 2834

#1AND#2 608

**EMBASE**

'pilonidal sinus'/exp OR (sacrococcygeal AND pilonidal AND sinus) OR (pilonidal AND cyst) OR (pilonidal AND cysts) OR (pilonidal AND disease) OR (pilonidal AND abscess) OR (sinus, AND pilonidal) OR (cyst, AND pilonidal) OR (cysts, AND pilonidal) 4199

'risk factor'/exp OR (risk AND determinants) OR (predisposing AND factors) OR (contributing AND factors) OR (causative AND factors) OR (risk AND contributors) OR (vulnerability AND factors) OR (dangerous AND factor) OR (influencing AND factor) OR determinants OR predispose OR (population AND at AND risk) OR (risk AND score) OR (risk AND factor AND score) OR (health AND correlates) OR (score, AND risk) OR (factor, AND risk) OR (precipitating AND factors) 3,349,474
#1AND#2 333

**Web of science**

#1 Pilonidal Sinus (Topic) or Sacrococcygeal Pilonidal Sinus (Topic) or Pilonidal Cyst (Topic) or Pilonidal Disease (Topic) or Pilonidal Abscess (Topic) or Sinus, Pilonidal (Topic) or Cyst, Pilonidal (Topic) and Preprint Citation Index (Exclude – Database) 3294

#2 Risk Factors (Topic) or Risk Determinants (Topic) or Predisposing Factors (Topic) or Contributing Factors (Topic) or Causative Factors (Topic) or Risk Contributors (Topic) or Vulnerability Factors (Topic) or dangerous factor (Topic) or influencing factor (Topic) or Determinants (Topic) or predispose (Topic) or Population at Risk (Topic) or Risk Score (Topic) or Risk Factor Score (Topic) or Health Correlates (Topic) or Score, Risk (Topic) or Factor, Risk (Topic) or Precipitating Factors (Topic) and Preprint Citation Index (Exclude–Database) 6,140,694

#3 #1 AND #2 251

**Cocharane library**


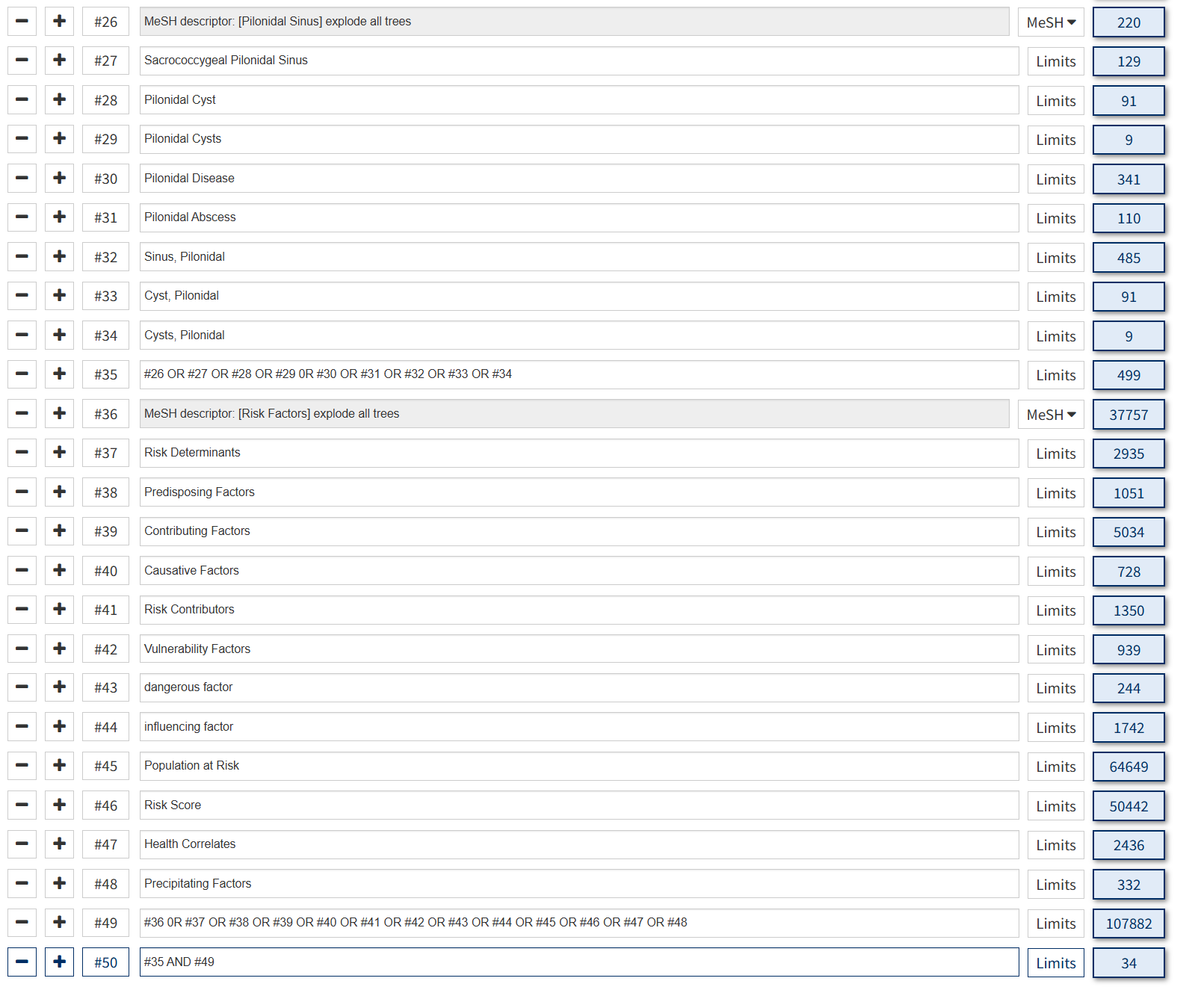

Supplement: Supplementary file 1 [file Datasheet1.docx]
