# Supplementary material for: Risk factors for sacrococcygeal pilonidal sinus: a systematic review and meta-analysis supplemented by genetic causal assessment
Source: Front Surg. 2026 Jan 7;12:1718589. doi: 10.3389/fsurg.2025.1718589 (PMC12819706; doi:10.3389/fsurg.2025.1718589)
Supplement: Supplementary file 2 [file Datasheet2.zip › Supplementary Data 2/MR_pipeline_after_confounding_SNPs_removal/ebi-a-GCST006095_finngen_R12_L12_PILONIDALCYST_20250626220939/01. finngen_R12_L12_PILONIDALCYST_scatter_plot.pptx]

## Slide 1
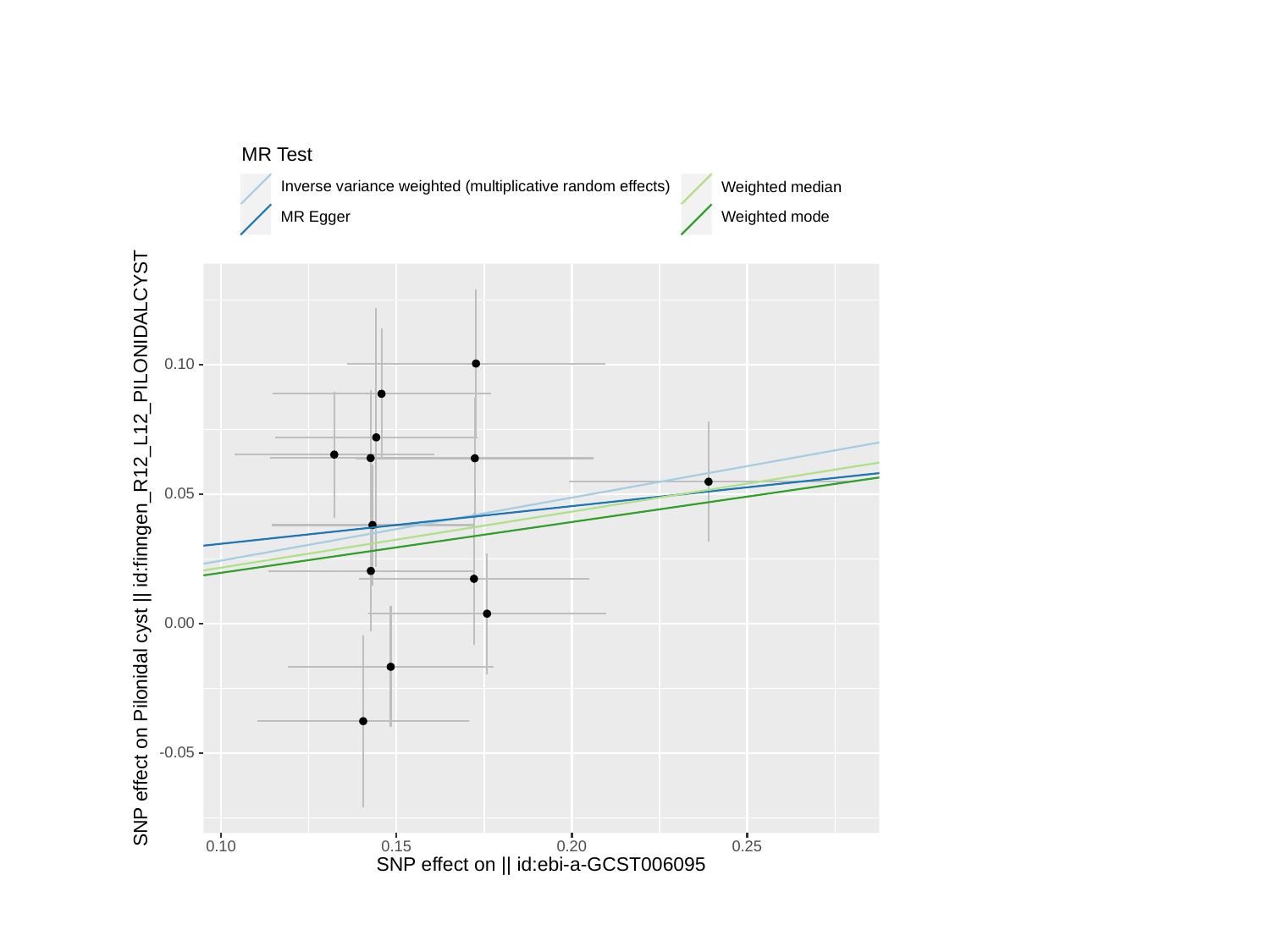

#
MR Test
Inverse variance weighted (multiplicative random effects)
Weighted median
MR Egger
Weighted mode
0.10
0.05
SNP effect on Pilonidal cyst || id:finngen_R12_L12_PILONIDALCYST
0.00
-0.05
0.10
0.15
0.20
0.25
SNP effect on || id:ebi-a-GCST006095
