# Supplementary material for: Risk factors for sacrococcygeal pilonidal sinus: a systematic review and meta-analysis supplemented by genetic causal assessment
Source: Front Surg. 2026 Jan 7;12:1718589. doi: 10.3389/fsurg.2025.1718589 (PMC12819706; doi:10.3389/fsurg.2025.1718589)
Supplement: Supplementary file 2 [file Datasheet2.zip › Supplementary Data 2/MR_pipeline_after_confounding_SNPs_removal/ebi-a-GCST006095_finngen_R12_L12_PILONIDALCYST_20250626220939/03. finngen_R12_L12_PILONIDALCYST_leaveone_plot.pptx]

## Slide 1
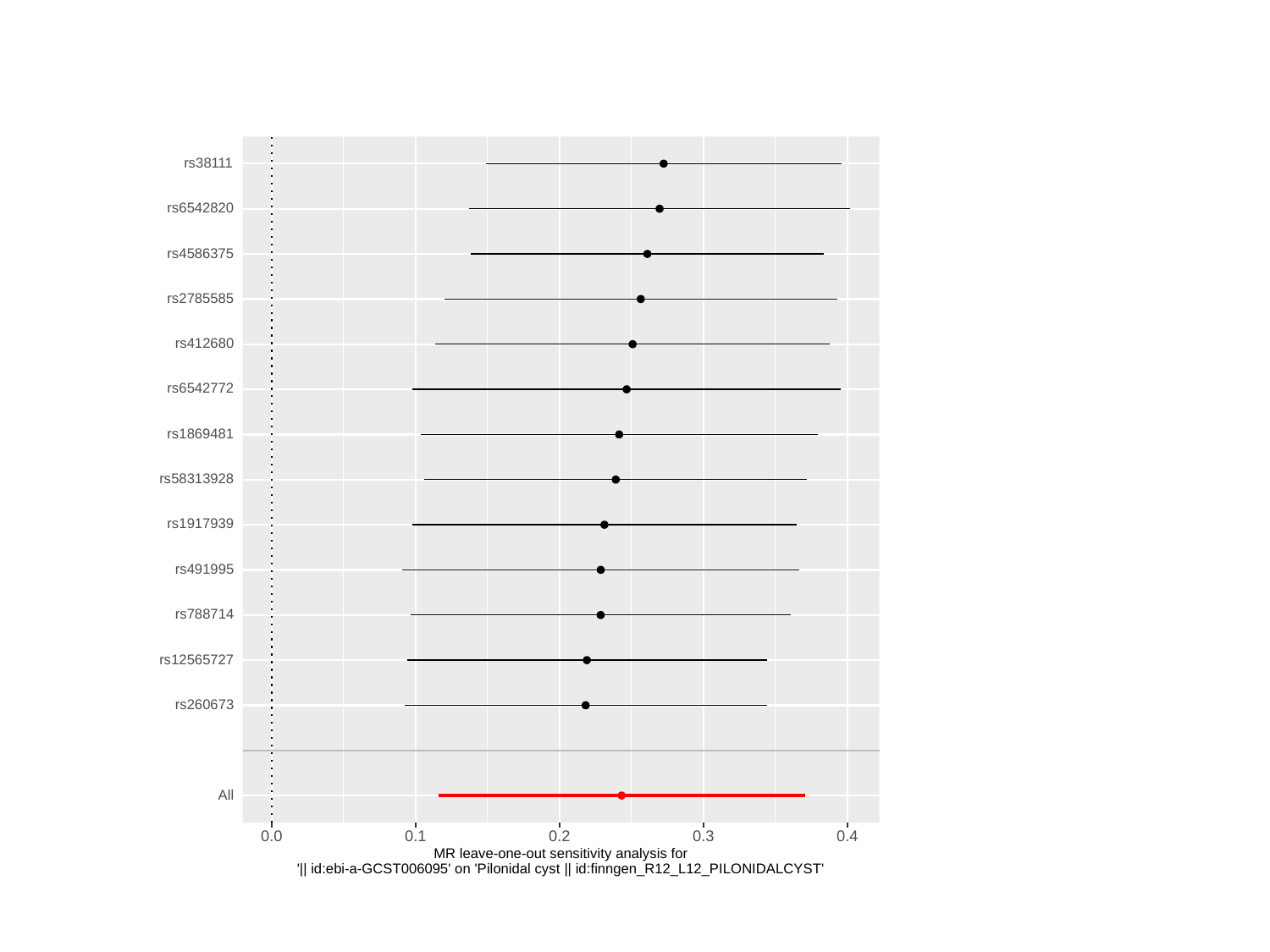

#
rs38111
rs6542820
rs4586375
rs2785585
rs412680
rs6542772
rs1869481
rs58313928
rs1917939
rs491995
rs788714
rs12565727
rs260673
All
0.3
0.0
0.1
0.2
0.4
MR leave-one-out sensitivity analysis for
'|| id:ebi-a-GCST006095' on 'Pilonidal cyst || id:finngen_R12_L12_PILONIDALCYST'
