# Supplementary material for: Risk factors for sacrococcygeal pilonidal sinus: a systematic review and meta-analysis supplemented by genetic causal assessment
Source: Front Surg. 2026 Jan 7;12:1718589. doi: 10.3389/fsurg.2025.1718589 (PMC12819706; doi:10.3389/fsurg.2025.1718589)
Supplement: Supplementary file 2 [file Datasheet2.zip › Supplementary Data 2/MR_pipeline_after_confounding_SNPs_removal/ebi-a-GCST90014023_finngen_R12_L12_PILONIDALCYST_20250627002938/03. finngen_R12_L12_PILONIDALCYST_leaveone_plot.pptx]

## Slide 1
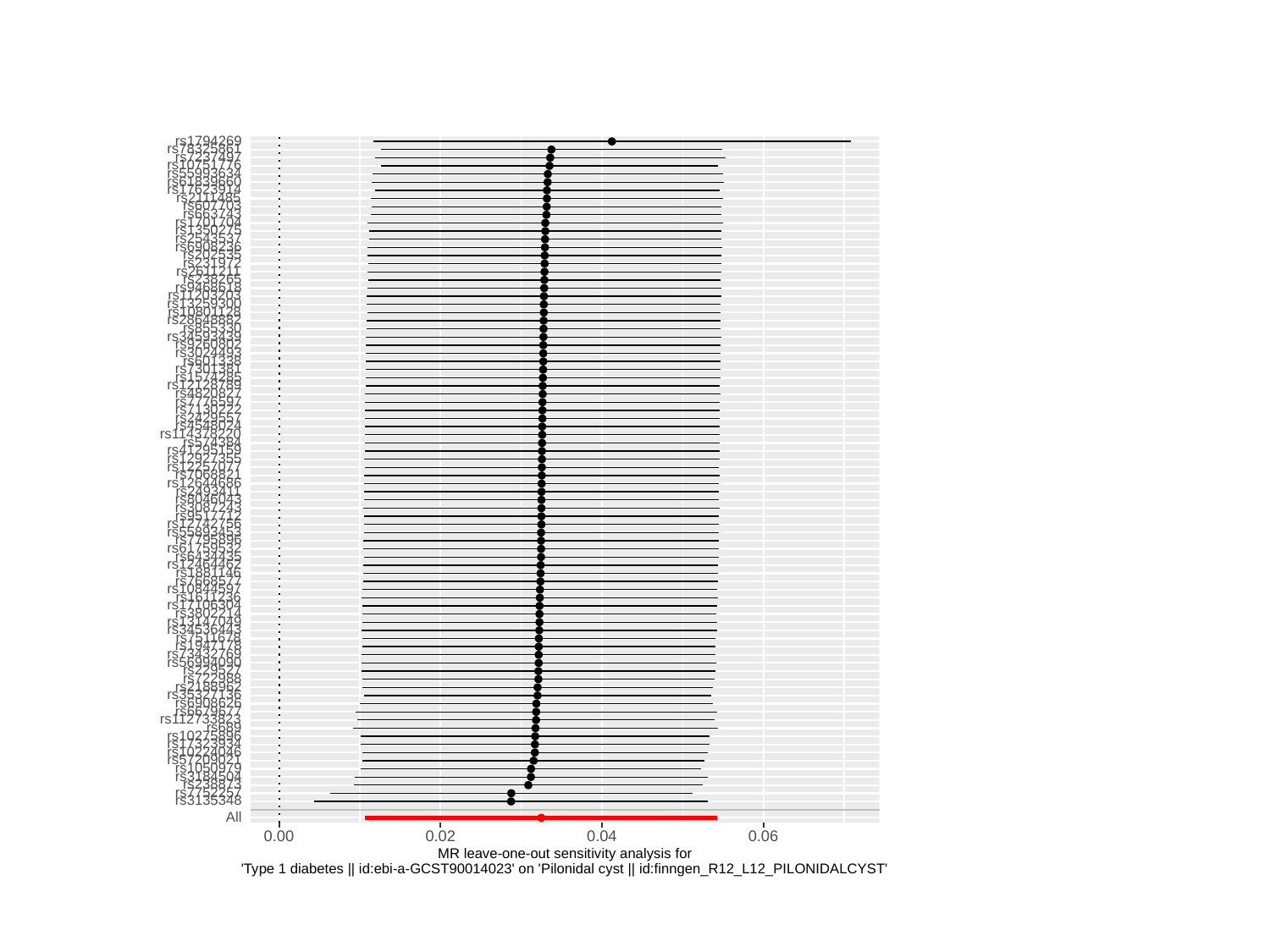

#
rs1794269
rs78325861
rs7237497
rs10751776
rs55993634
rs61839660
rs17623914
rs2111485
rs607703
rs663743
rs1701704
rs1350275
rs2543537
rs6908236
rs202535
rs231972
rs2611211
rs238265
rs9468618
rs11203203
rs13259300
rs10801128
rs28648882
rs855330
rs34593439
rs9260802
rs3024493
rs601338
rs7301381
rs1574285
rs12128789
rs4820827
rs7776597
rs7130222
rs2429557
rs4548024
rs114378220
rs574384
rs41295159
rs12927355
rs12257077
rs7068821
rs12644686
rs2493411
rs8046043
rs3087243
rs9517712
rs12742756
rs55893453
rs7795896
rs61759532
rs6434435
rs12464462
rs1881146
rs7668577
rs10844597
rs1611236
rs17106304
rs3802214
rs13147049
rs34536443
rs7511678
rs1947178
rs73432769
rs56994090
rs229527
rs722988
rs2188962
rs35327136
rs6908626
rs6679677
rs112733823
rs689
rs10275896
rs17323934
rs10224046
rs57209021
rs1050979
rs3184504
rs238873
rs7752257
rs3135348
All
0.00
0.02
0.04
0.06
MR leave-one-out sensitivity analysis for
'Type 1 diabetes || id:ebi-a-GCST90014023' on 'Pilonidal cyst || id:finngen_R12_L12_PILONIDALCYST'
