# Supplementary material for: Risk factors for sacrococcygeal pilonidal sinus: a systematic review and meta-analysis supplemented by genetic causal assessment
Source: Front Surg. 2026 Jan 7;12:1718589. doi: 10.3389/fsurg.2025.1718589 (PMC12819706; doi:10.3389/fsurg.2025.1718589)
Supplement: Supplementary file 2 [file Datasheet2.zip › Supplementary Data 2/MR_pipeline_after_confounding_SNPs_removal/finngen_R12_L12_ACNE_finngen_R12_L12_PILONIDALCYST_20251109185740/02. finngen_R12_L12_PILONIDALCYST_forest_plot.pptx]

## Slide 1
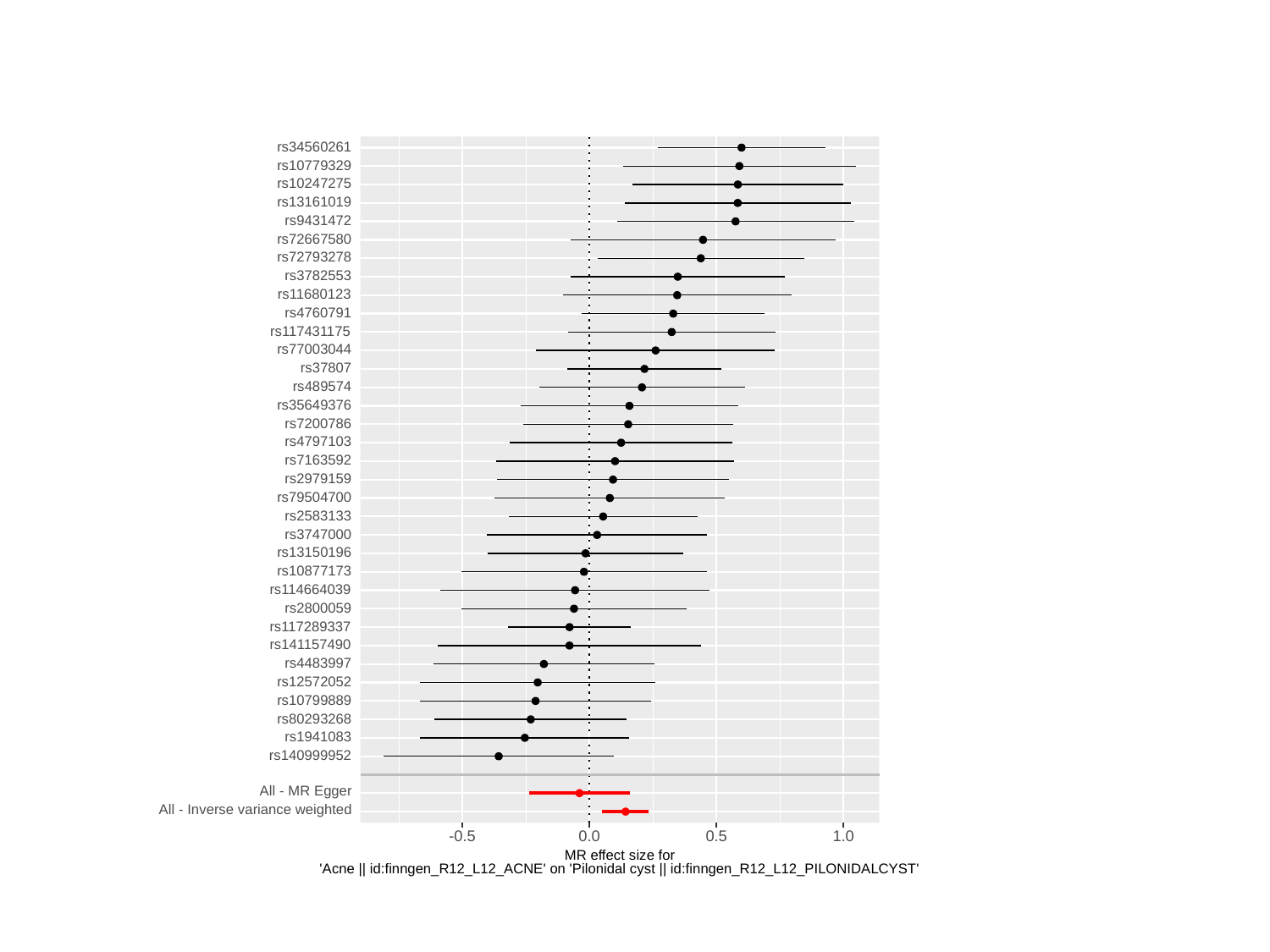

#
rs34560261
rs10779329
rs10247275
rs13161019
rs9431472
rs72667580
rs72793278
rs3782553
rs11680123
rs4760791
rs117431175
rs77003044
rs37807
rs489574
rs35649376
rs7200786
rs4797103
rs7163592
rs2979159
rs79504700
rs2583133
rs3747000
rs13150196
rs10877173
rs114664039
rs2800059
rs117289337
rs141157490
rs4483997
rs12572052
rs10799889
rs80293268
rs1941083
rs140999952
All - MR Egger
All - Inverse variance weighted
-0.5
0.0
0.5
1.0
MR effect size for
'Acne || id:finngen_R12_L12_ACNE' on 'Pilonidal cyst || id:finngen_R12_L12_PILONIDALCYST'
