# Supplementary material for: Risk factors for sacrococcygeal pilonidal sinus: a systematic review and meta-analysis supplemented by genetic causal assessment
Source: Front Surg. 2026 Jan 7;12:1718589. doi: 10.3389/fsurg.2025.1718589 (PMC12819706; doi:10.3389/fsurg.2025.1718589)
Supplement: Supplementary file 2 [file Datasheet2.zip › Supplementary Data 2/MR_pipeline_after_confounding_SNPs_removal/finngen_R12_L12_ACNE_finngen_R12_L12_PILONIDALCYST_20251109185740/03. finngen_R12_L12_PILONIDALCYST_leaveone_plot.pptx]

## Slide 1
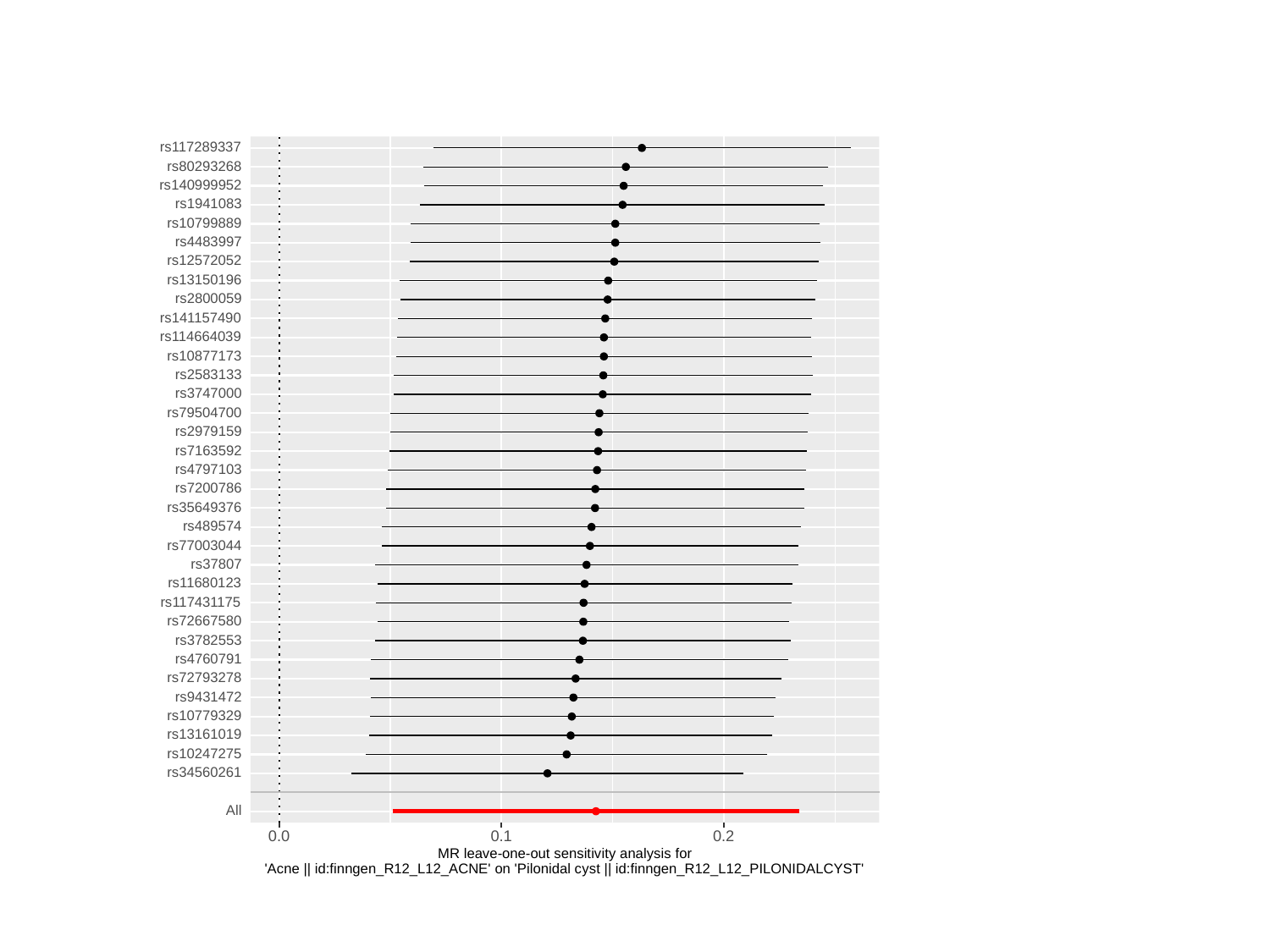

#
rs117289337
rs80293268
rs140999952
rs1941083
rs10799889
rs4483997
rs12572052
rs13150196
rs2800059
rs141157490
rs114664039
rs10877173
rs2583133
rs3747000
rs79504700
rs2979159
rs7163592
rs4797103
rs7200786
rs35649376
rs489574
rs77003044
rs37807
rs11680123
rs117431175
rs72667580
rs3782553
rs4760791
rs72793278
rs9431472
rs10779329
rs13161019
rs10247275
rs34560261
All
0.0
0.1
0.2
MR leave-one-out sensitivity analysis for
'Acne || id:finngen_R12_L12_ACNE' on 'Pilonidal cyst || id:finngen_R12_L12_PILONIDALCYST'
