# Supplementary material for: Risk factors for sacrococcygeal pilonidal sinus: a systematic review and meta-analysis supplemented by genetic causal assessment
Source: Front Surg. 2026 Jan 7;12:1718589. doi: 10.3389/fsurg.2025.1718589 (PMC12819706; doi:10.3389/fsurg.2025.1718589)
Supplement: Supplementary file 2 [file Datasheet2.zip › Supplementary Data 2/MR_pipeline_after_confounding_SNPs_removal/finngen_R12_L12_HIDRADENITISSUP_finngen_R12_L12_PILONIDALCYST_20250626221908/02. finngen_R12_L12_PILONIDALCYST_forest_plot.pptx]

## Slide 1
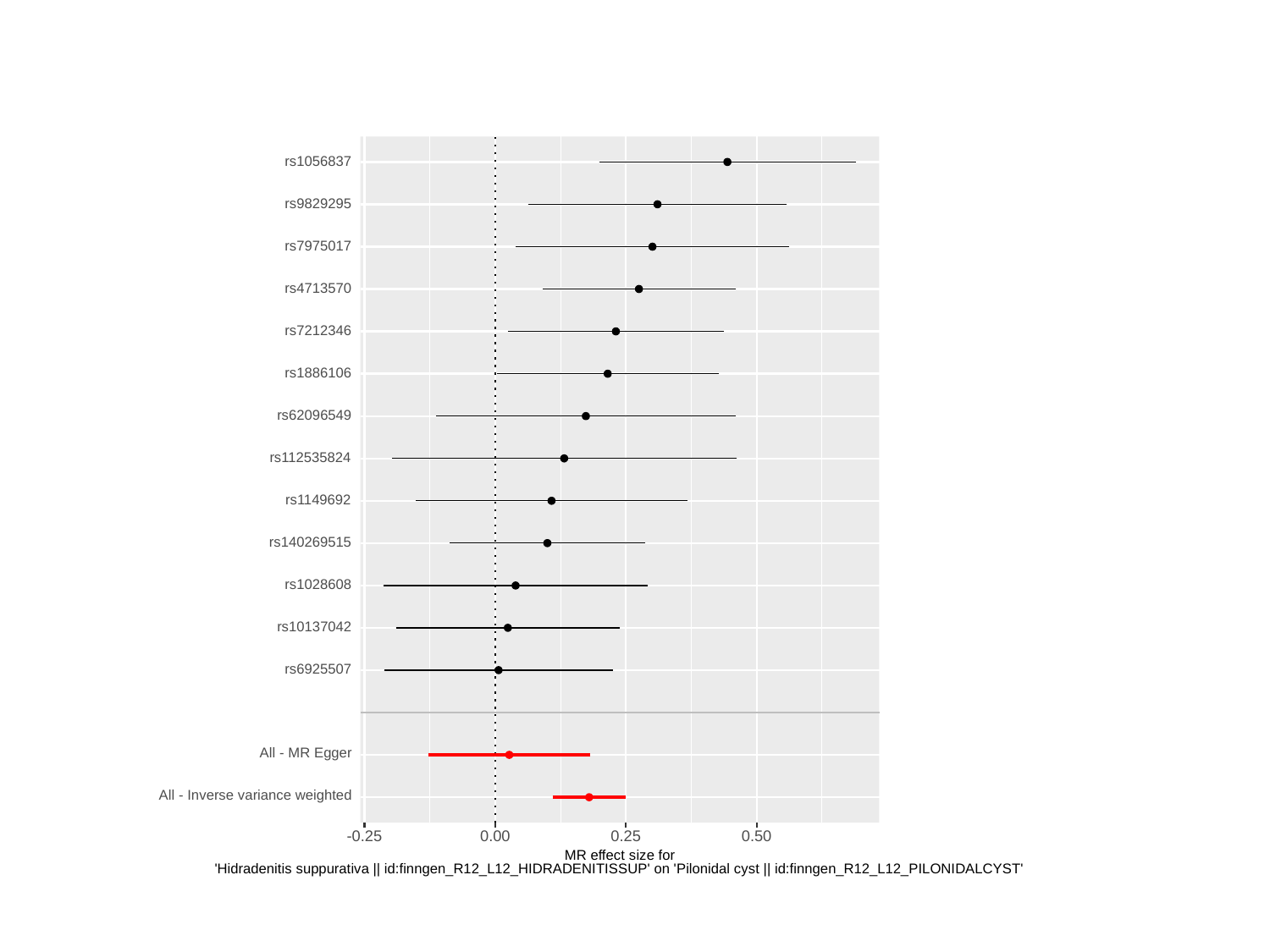

#
rs1056837
rs9829295
rs7975017
rs4713570
rs7212346
rs1886106
rs62096549
rs112535824
rs1149692
rs140269515
rs1028608
rs10137042
rs6925507
All - MR Egger
All - Inverse variance weighted
-0.25
0.00
0.25
0.50
MR effect size for
'Hidradenitis suppurativa || id:finngen_R12_L12_HIDRADENITISSUP' on 'Pilonidal cyst || id:finngen_R12_L12_PILONIDALCYST'
