# Supplementary material for: Risk factors for sacrococcygeal pilonidal sinus: a systematic review and meta-analysis supplemented by genetic causal assessment
Source: Front Surg. 2026 Jan 7;12:1718589. doi: 10.3389/fsurg.2025.1718589 (PMC12819706; doi:10.3389/fsurg.2025.1718589)
Supplement: Supplementary file 2 [file Datasheet2.zip › Supplementary Data 2/MR_pipeline_after_confounding_SNPs_removal/finngen_R12_L12_HIDRADENITISSUP_ukb-b-5617/02. ukb-b-5617_forest_plot.pptx]

## Slide 1
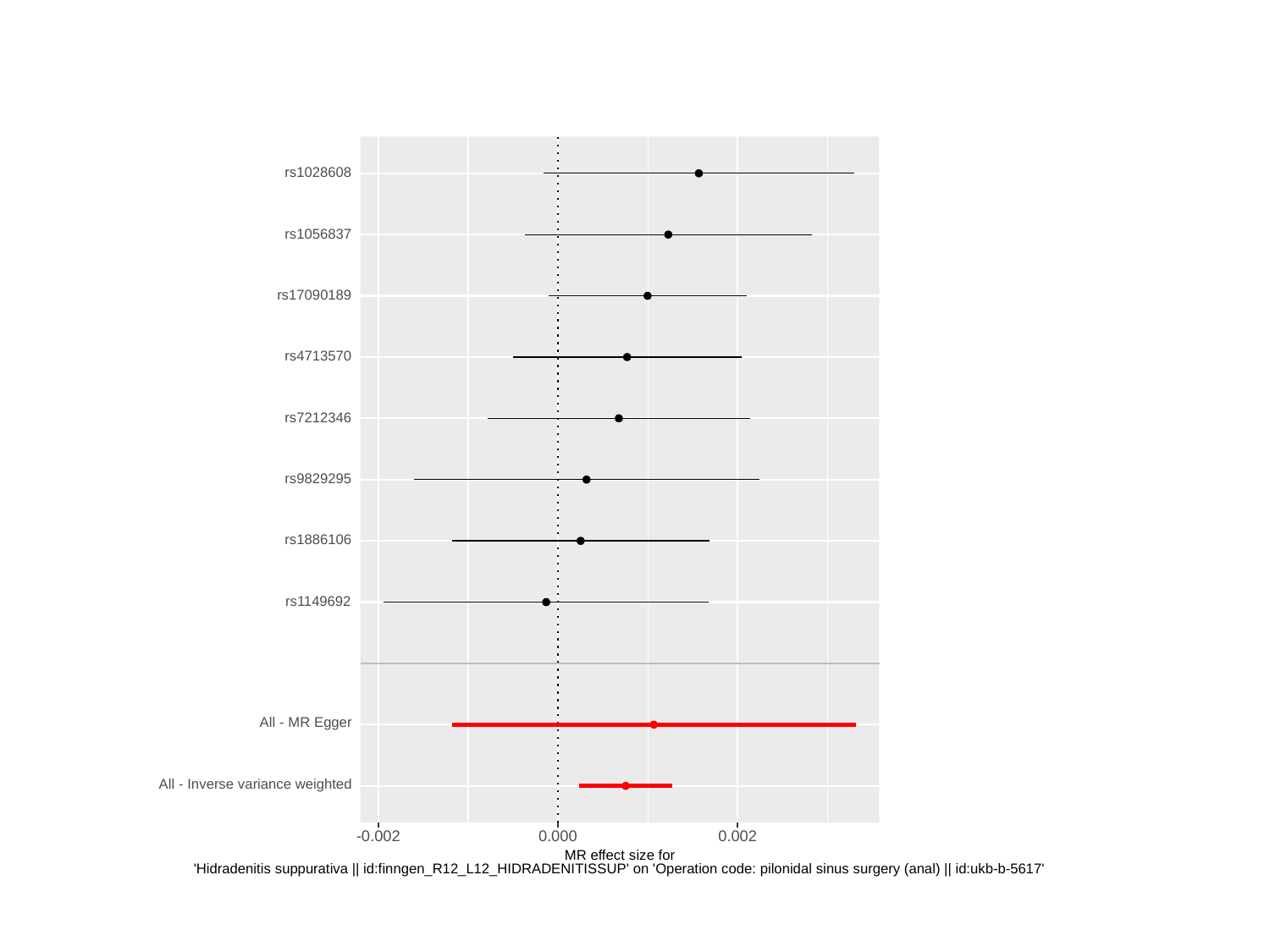

#
rs1028608
rs1056837
rs17090189
rs4713570
rs7212346
rs9829295
rs1886106
rs1149692
All - MR Egger
All - Inverse variance weighted
-0.002
0.000
0.002
MR effect size for
'Hidradenitis suppurativa || id:finngen_R12_L12_HIDRADENITISSUP' on 'Operation code: pilonidal sinus surgery (anal) || id:ukb-b-5617'
