# Supplementary material for: Risk factors for sacrococcygeal pilonidal sinus: a systematic review and meta-analysis supplemented by genetic causal assessment
Source: Front Surg. 2026 Jan 7;12:1718589. doi: 10.3389/fsurg.2025.1718589 (PMC12819706; doi:10.3389/fsurg.2025.1718589)
Supplement: Supplementary file 2 [file Datasheet2.zip › Supplementary Data 2/MR_pipeline_after_confounding_SNPs_removal/finngen_R12_L12_HIDRADENITISSUP_ukb-b-5617/03. ukb-b-5617 _leaveone_plot.pptx]

## Slide 1
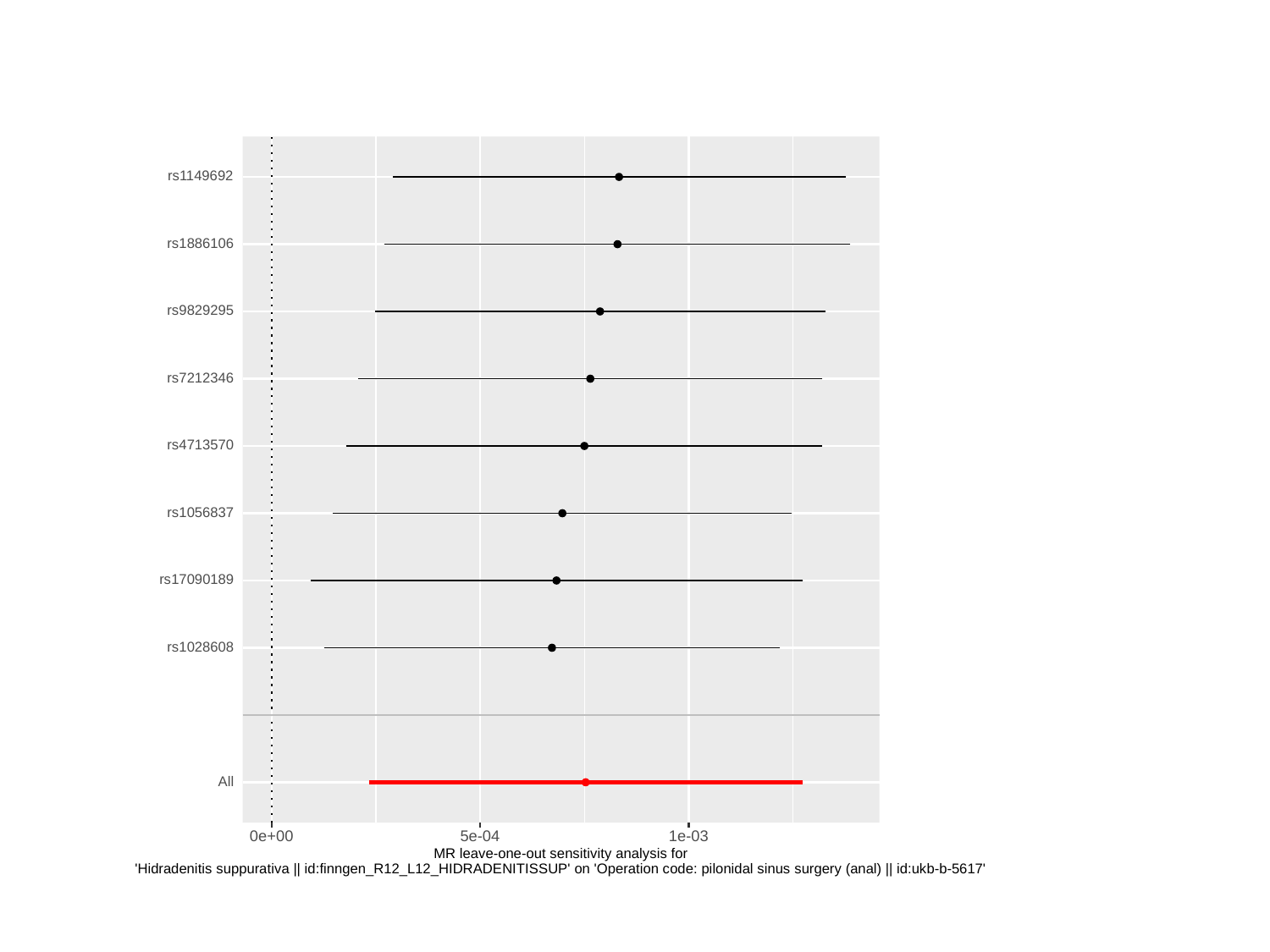

#
rs1149692
rs1886106
rs9829295
rs7212346
rs4713570
rs1056837
rs17090189
rs1028608
All
1e-03
0e+00
5e-04
MR leave-one-out sensitivity analysis for
'Hidradenitis suppurativa || id:finngen_R12_L12_HIDRADENITISSUP' on 'Operation code: pilonidal sinus surgery (anal) || id:ukb-b-5617'
