# Supplementary material for: Risk factors for sacrococcygeal pilonidal sinus: a systematic review and meta-analysis supplemented by genetic causal assessment
Source: Front Surg. 2026 Jan 7;12:1718589. doi: 10.3389/fsurg.2025.1718589 (PMC12819706; doi:10.3389/fsurg.2025.1718589)
Supplement: Supplementary file 2 [file Datasheet2.zip › Supplementary Data 2/MR_pipeline_after_confounding_SNPs_removal/GCST90245818_ukb-b-5617_20251109205253/01. ukb-b-5617_scatter_plot.pptx]

## Slide 1
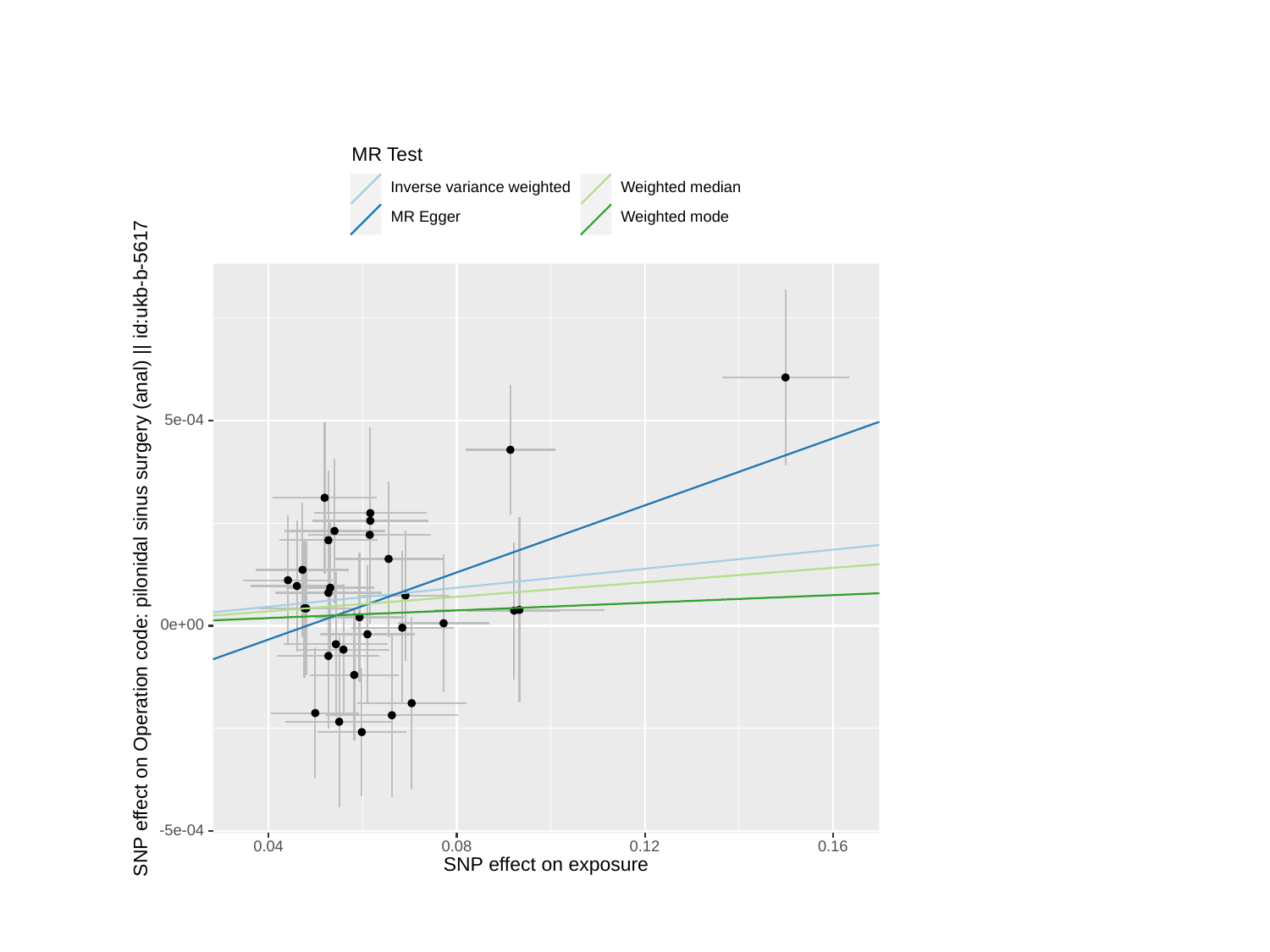

#
MR Test
Inverse variance weighted
Weighted median
MR Egger
Weighted mode
5e-04
SNP effect on Operation code: pilonidal sinus surgery (anal) || id:ukb-b-5617
0e+00
-5e-04
0.04
0.08
0.12
0.16
SNP effect on exposure
