# Supplementary material for: Risk factors for sacrococcygeal pilonidal sinus: a systematic review and meta-analysis supplemented by genetic causal assessment
Source: Front Surg. 2026 Jan 7;12:1718589. doi: 10.3389/fsurg.2025.1718589 (PMC12819706; doi:10.3389/fsurg.2025.1718589)
Supplement: Supplementary file 2 [file Datasheet2.zip › Supplementary Data 2/MR_pipeline_after_confounding_SNPs_removal/GCST90245818_ukb-b-5617_20251109205253/02. ukb-b-5617_forest_plot.pptx]

## Slide 1
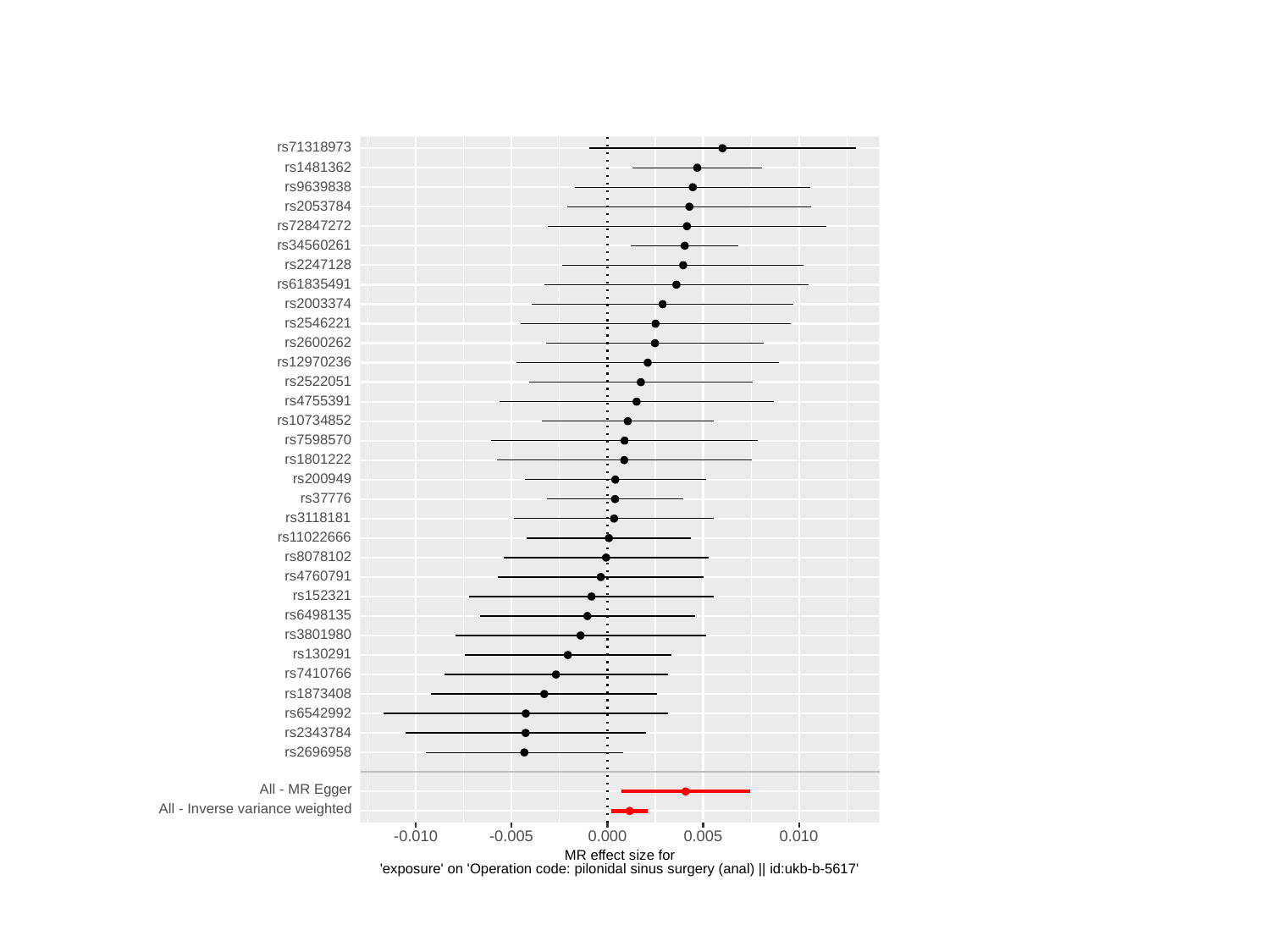

#
rs71318973
rs1481362
rs9639838
rs2053784
rs72847272
rs34560261
rs2247128
rs61835491
rs2003374
rs2546221
rs2600262
rs12970236
rs2522051
rs4755391
rs10734852
rs7598570
rs1801222
rs200949
rs37776
rs3118181
rs11022666
rs8078102
rs4760791
rs152321
rs6498135
rs3801980
rs130291
rs7410766
rs1873408
rs6542992
rs2343784
rs2696958
All - MR Egger
All - Inverse variance weighted
-0.010
-0.005
0.000
0.005
0.010
MR effect size for
'exposure' on 'Operation code: pilonidal sinus surgery (anal) || id:ukb-b-5617'
