# Supplementary material for: Risk factors for sacrococcygeal pilonidal sinus: a systematic review and meta-analysis supplemented by genetic causal assessment
Source: Front Surg. 2026 Jan 7;12:1718589. doi: 10.3389/fsurg.2025.1718589 (PMC12819706; doi:10.3389/fsurg.2025.1718589)
Supplement: Supplementary file 2 [file Datasheet2.zip › Supplementary Data 2/MR_pipeline_after_confounding_SNPs_removal/GCST90245818_ukb-b-5617_20251109205253/03. ukb-b-5617_leaveone_plot.pptx]

## Slide 1
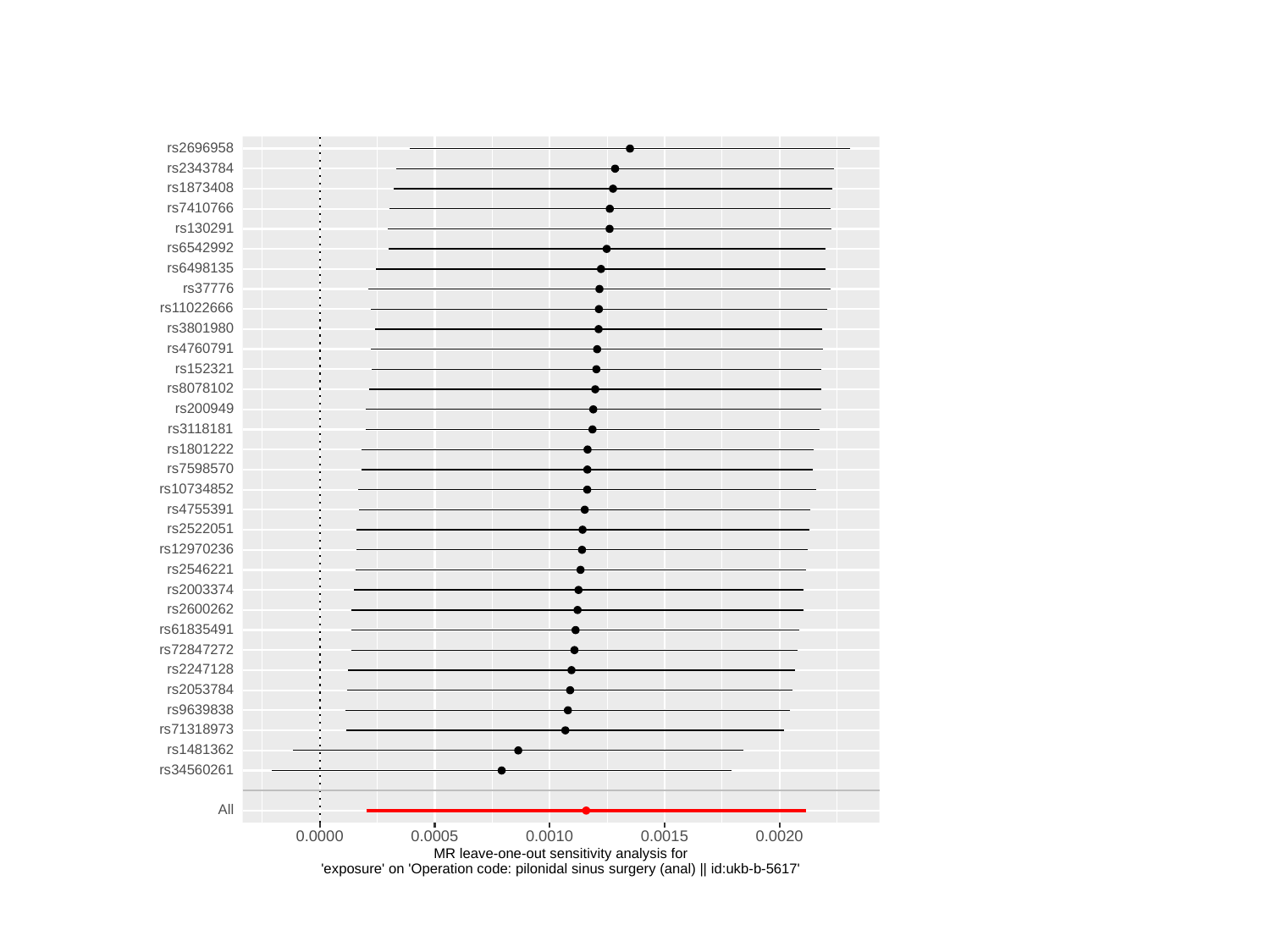

#
rs2696958
rs2343784
rs1873408
rs7410766
rs130291
rs6542992
rs6498135
rs37776
rs11022666
rs3801980
rs4760791
rs152321
rs8078102
rs200949
rs3118181
rs1801222
rs7598570
rs10734852
rs4755391
rs2522051
rs12970236
rs2546221
rs2003374
rs2600262
rs61835491
rs72847272
rs2247128
rs2053784
rs9639838
rs71318973
rs1481362
rs34560261
All
0.0000
0.0005
0.0010
0.0015
0.0020
MR leave-one-out sensitivity analysis for
'exposure' on 'Operation code: pilonidal sinus surgery (anal) || id:ukb-b-5617'
