# Supplementary material for: Risk factors for sacrococcygeal pilonidal sinus: a systematic review and meta-analysis supplemented by genetic causal assessment
Source: Front Surg. 2026 Jan 7;12:1718589. doi: 10.3389/fsurg.2025.1718589 (PMC12819706; doi:10.3389/fsurg.2025.1718589)
Supplement: Supplementary file 2 [file Datasheet2.zip › Supplementary Data 2/MR_pipeline_after_confounding_SNPs_removal/ieu-a-93_finngen_R12_L12_PILONIDALCYST_20250626233850/03. finngen_R12_L12_PILONIDALCYST_leaveone_plot.pptx]

## Slide 1
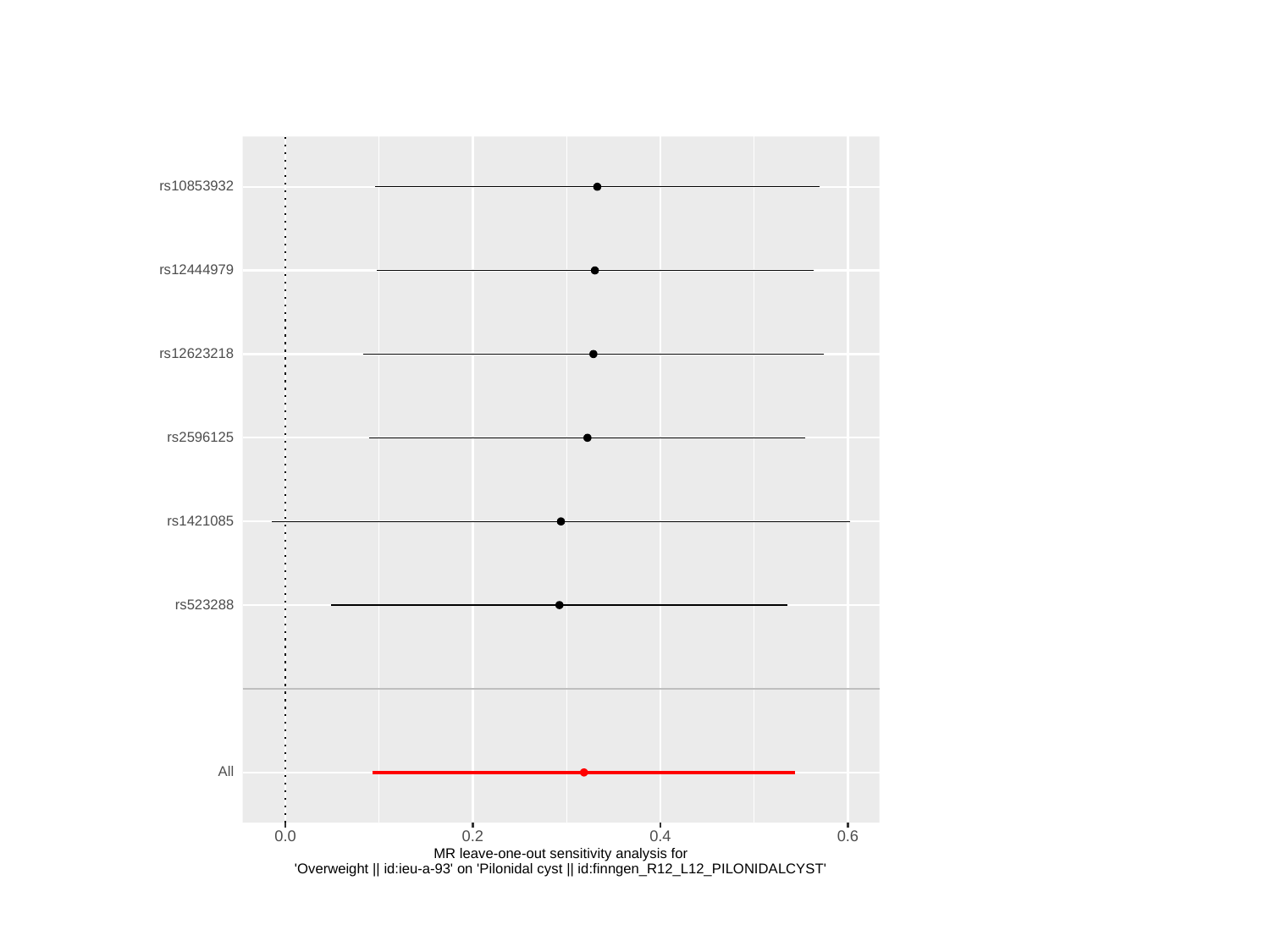

#
rs10853932
rs12444979
rs12623218
rs2596125
rs1421085
rs523288
All
0.0
0.2
0.4
0.6
MR leave-one-out sensitivity analysis for
'Overweight || id:ieu-a-93' on 'Pilonidal cyst || id:finngen_R12_L12_PILONIDALCYST'
