# Supplementary material for: Risk factors for sacrococcygeal pilonidal sinus: a systematic review and meta-analysis supplemented by genetic causal assessment
Source: Front Surg. 2026 Jan 7;12:1718589. doi: 10.3389/fsurg.2025.1718589 (PMC12819706; doi:10.3389/fsurg.2025.1718589)
Supplement: Supplementary file 2 [file Datasheet2.zip › Supplementary Data 2/MR_pipeline_after_confounding_SNPs_removal/ieu-b-40_finngen_R12_L12_PILONIDALCYST_20250626231420/02. finngen_R12_L12_PILONIDALCYST_forest_plot.pptx]

## Slide 1
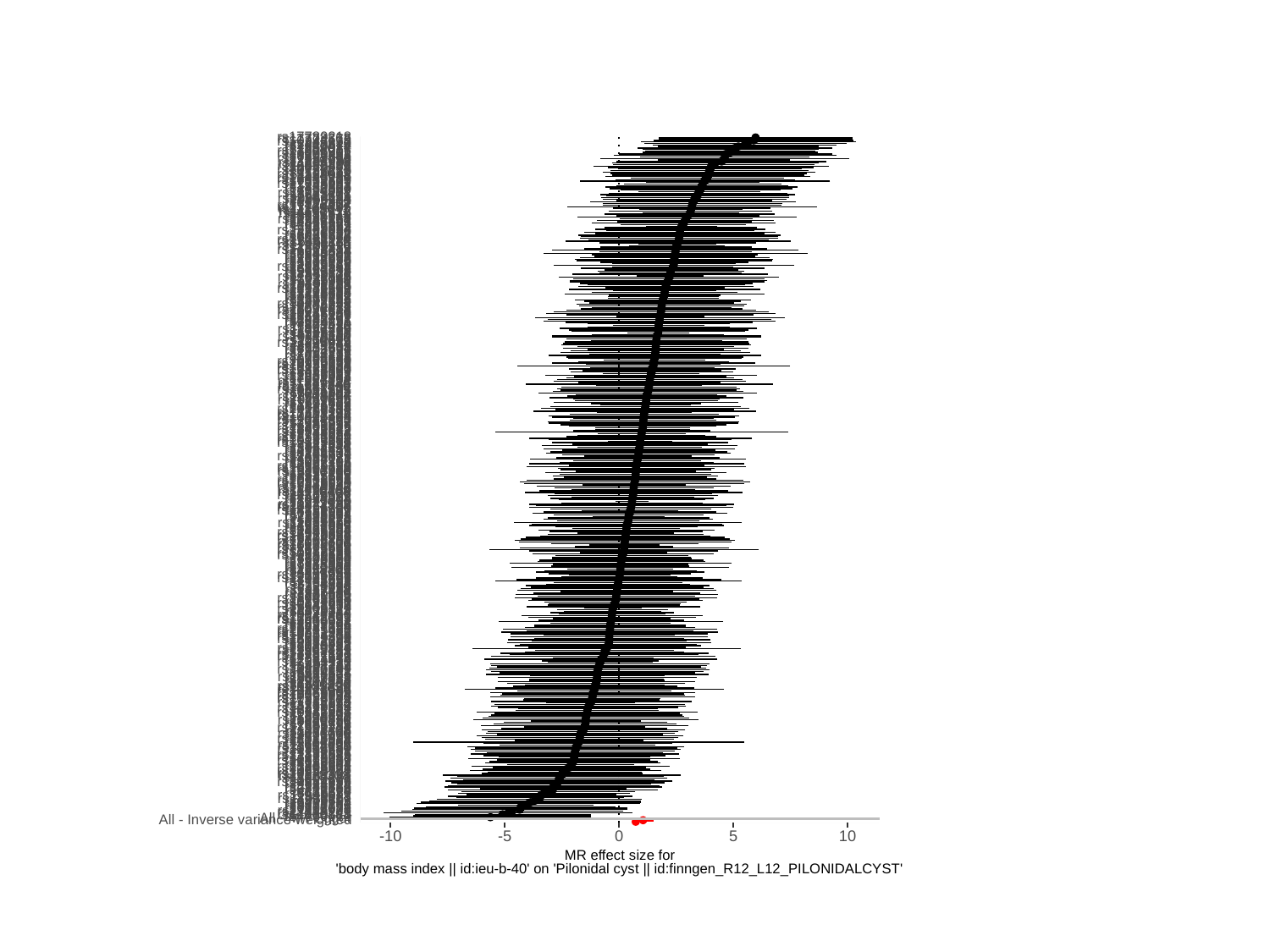

#
rs17789218
rs11773362
rs7724675
rs10248136
rs947612
rs2425840
rs7640424
rs1830074
rs1188017
rs17203016
rs1075901
rs17001561
rs9300422
rs6692586
rs17499593
rs7557796
rs2283093
rs11115176
rs4906908
rs12933482
rs6050446
rs4072917
rs10750215
rs4783830
rs1112613
rs12564992
rs1268065
rs12448257
rs16849710
rs7933205
rs7551507
rs17636031
rs3749897
rs1836303
rs1430387
rs33500
rs2694047
rs11739877
rs9375702
rs7683836
rs1187352
rs10968114
rs559231
rs1465900
rs6512302
rs7172627
rs17425707
rs13184896
rs11165643
rs11185111
rs4671328
rs11150911
rs9951619
rs3935648
rs9989141
rs11736228
rs6587552
rs4858193
rs4639527
rs6265
rs7637852
rs845084
rs12762034
rs852056
rs7780752
rs7826312
rs8065336
rs9845966
rs17551974
rs12098284
rs6461115
rs7498665
rs17238110
rs1503526
rs4307239
rs17663412
rs215634
rs9688431
rs1948080
rs6500208
rs7615297
rs7334078
rs4937870
rs1928295
rs3800229
rs2875762
rs12939549
rs2143253
rs6804842
rs9650755
rs2281819
rs2543132
rs7550711
rs17311369
rs8036040
rs7222349
rs1241986
rs339991
rs10192119
rs2791653
rs6985109
rs12932660
rs1579557
rs7102454
rs2423668
rs4740619
rs7488867
rs4516268
rs2367112
rs3807645
rs1477199
rs10742752
rs6785245
rs12602912
rs872281
rs11030618
rs9927848
rs9783858
rs17446257
rs9816226
rs6591407
rs1492767
rs4842491
rs2600226
rs9284814
rs879620
rs592483
rs1891216
rs11856579
rs1784460
rs987237
rs1528435
rs4148155
rs10478110
rs380857
rs1218822
rs1150659
rs12905439
rs784944
rs2943465
rs4954638
rs273504
rs2903971
rs7594289
rs6235
rs7694732
rs1064213
rs754635
rs9304665
rs10962550
rs175165
rs10920678
rs10858334
rs8123881
rs16871902
rs12364470
rs1472169
rs40067
rs1656377
rs12936083
rs2361988
rs7024334
rs11251352
rs7925214
rs11889536
rs11611246
rs11781699
rs287104
rs12652212
rs9615905
rs8071182
rs7730004
rs3904244
rs11908637
rs930295
rs10211055
rs1707322
rs1321432
rs1993709
rs1896767
rs4518345
rs10169594
rs6712
rs17207196
rs17113297
rs12922346
rs10408324
rs4820408
rs11084553
rs12718572
rs2051559
rs1285997
rs12981256
rs208015
rs12429545
rs8097672
rs17535082
rs2744974
rs7715256
rs12593036
rs2836964
rs13191362
rs10182181
rs12629015
rs1327259
rs11538
rs427943
rs3764835
rs1982441
rs4936175
rs8181823
rs4864201
rs17405819
rs1452075
rs4358081
rs977747
rs3007105
rs1982725
rs2065418
rs13147390
rs10269783
rs901630
rs12888955
rs13110266
rs2357760
rs3772882
rs7535528
rs6841761
rs10733051
rs10518694
rs12328930
rs7239575
rs9571687
rs12680842
rs11496125
rs2429150
rs11170468
rs3736485
rs12334877
rs7599312
rs4414033
rs7598508
rs8047395
rs1927790
rs11173522
rs12422552
rs10971709
rs7196720
rs13329567
rs4851029
rs6556301
rs1266874
rs7138803
rs2196618
rs962273
rs946824
rs1535660
rs11615578
rs4968656
rs7983065
rs4981693
rs9367368
rs1436344
rs12049202
rs6593688
rs17710386
rs13250058
rs774246
rs9806742
rs17056301
rs2608703
rs11672660
rs10132280
rs1409818
rs12675063
rs7037266
rs1412235
rs10867256
rs12150665
rs429343
rs895330
rs7206608
rs7970953
rs3844598
rs7788008
rs7318817
rs9547153
rs1320903
rs1158805
rs4952843
rs7871866
rs16903285
rs2325036
rs12022461
rs902695
rs1445652
rs7181498
rs756717
rs3754963
rs326896
rs331966
rs7117238
rs657452
rs2235564
rs1937683
rs200810
rs2605603
rs12041258
rs765875
rs9294260
rs10197031
rs1048932
rs6443750
rs543874
rs17806379
rs2820311
rs4757144
rs9926784
rs10747488
rs11945861
rs17513613
rs12044597
rs9408882
rs4929923
rs7899106
rs2693826
rs1421334
rs10741329
rs10795422
rs16822990
rs6561943
rs16851483
rs17014375
rs709400
rs16889835
rs4012234
rs9382285
rs2124499
rs8090983
rs2228213
rs13069244
rs11505821
rs2242189
rs2832283
rs13287131
rs17767510
rs4237643
rs7761673
rs7084454
rs12369179
rs6734537
rs491711
rs1884389
rs294704
rs10827649
rs4800191
rs6764533
rs818524
rs2481665
rs10811871
rs8097783
rs2479958
rs2907948
rs2306537
rs7811342
rs10492229
rs11105839
rs4722398
rs10878946
rs17424296
rs865809
rs10768994
rs7998796
rs10953740
rs17724992
rs7819514
rs11713193
rs768840
rs3732084
rs4818225
rs12299814
rs6471941
rs825688
rs10247983
rs1365466
rs1804528
rs1538247
rs999889
rs11880870
rs9522285
rs7869771
rs1885728
rs2365389
rs17399237
rs998732
rs1371108
rs1681740
rs11656076
rs9426003
rs4713436
rs12416812
rs7025938
rs4151664
rs9362662
rs11609659
rs11738695
rs262130
rs10915840
rs7704281
rs217671
rs1399896
rs12888545
rs13174863
rs7685048
rs1477887
rs10510419
rs156201
rs4986044
rs1863652
rs13263601
rs3806572
rs10259786
rs4556997
rs10942267
rs12779328
rs11118308
rs2007231
rs3800637
rs1522569
rs10984756
rs4813619
rs934224
rs4148866
rs2861683
rs6772756
rs38314
rs538579
rs6448587
rs11951673
rs349088
rs17033117
rs4589691
rs876605
rs9288754
rs9538162
rs3977755
rs6673081
rs7703576
rs17119937
rs17535749
rs4880341
rs11066188
rs4430672
All - MR Egger
All - Inverse variance weighted
-10
0
10
-5
5
MR effect size for
'body mass index || id:ieu-b-40' on 'Pilonidal cyst || id:finngen_R12_L12_PILONIDALCYST'
