# Supplementary material for: Risk factors for sacrococcygeal pilonidal sinus: a systematic review and meta-analysis supplemented by genetic causal assessment
Source: Front Surg. 2026 Jan 7;12:1718589. doi: 10.3389/fsurg.2025.1718589 (PMC12819706; doi:10.3389/fsurg.2025.1718589)
Supplement: Supplementary file 2 [file Datasheet2.zip › Supplementary Data 2/MR_pipeline_after_confounding_SNPs_removal/ieu-b-40_finngen_R12_L12_PILONIDALCYST_20250626231420/03. finngen_R12_L12_PILONIDALCYST_leaveone_plot.pptx]

## Slide 1
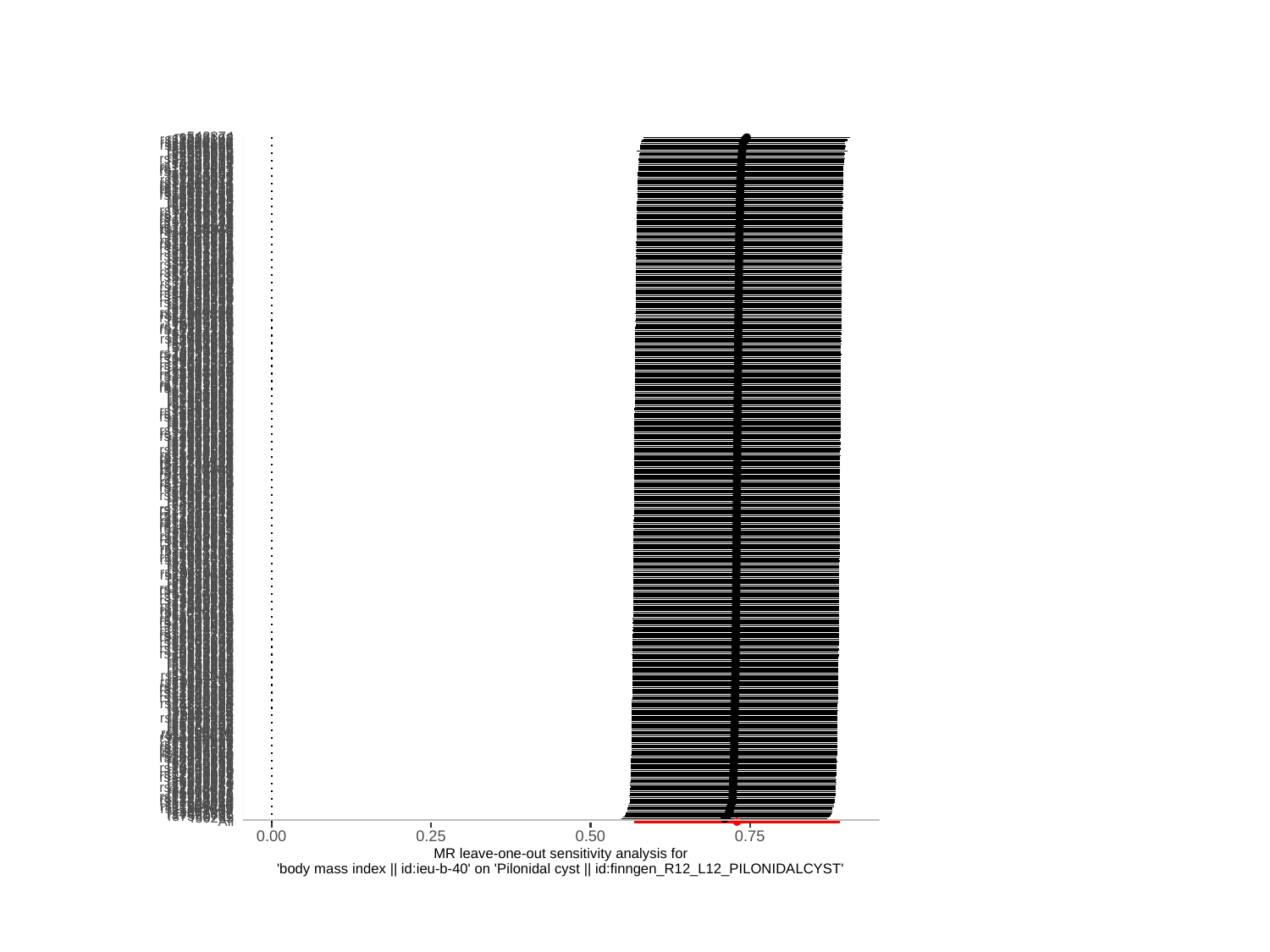

#
rs543874
rs9538162
rs11713193
rs4880341
rs11066188
rs6448587
rs11880870
rs4986044
rs2861683
rs8047395
rs4430672
rs2365389
rs349088
rs7025938
rs4237643
rs12369179
rs3977755
rs538579
rs4556997
rs38314
rs13263601
rs10942267
rs11951673
rs13174863
rs1477887
rs7084454
rs6673081
rs7703576
rs2820311
rs17119937
rs3806572
rs12299814
rs11609659
rs6471941
rs17724992
rs10953740
rs17033117
rs11672660
rs4589691
rs10510419
rs4813619
rs9288754
rs876605
rs8097783
rs2481665
rs2479958
rs3800637
rs9926784
rs7811342
rs17535749
rs156201
rs11656076
rs217671
rs10259786
rs17806379
rs6772756
rs17399237
rs9522285
rs12888545
rs934224
rs17513613
rs11118308
rs10878946
rs4148866
rs4929923
rs10984756
rs1863652
rs2007231
rs9362662
rs11505821
rs1412235
rs12416812
rs16903285
rs4757144
rs1681740
rs16851483
rs7704281
rs657452
rs7869771
rs10492229
rs1522569
rs4818225
rs262130
rs1371108
rs4713436
rs10247983
rs7138803
rs1399896
rs1320903
rs7685048
rs10915840
rs1365466
rs9426003
rs12779328
rs1804528
rs865809
rs768840
rs1048932
rs11738695
rs998732
rs10768994
rs2306537
rs3732084
rs2242189
rs10197031
rs2325036
rs10132280
rs1885728
rs709400
rs1538247
rs12044597
rs2228213
rs2693826
rs4012234
rs7899106
rs7819514
rs7181498
rs12049202
rs11105839
rs7998796
rs17424296
rs2907948
rs9294260
rs6764533
rs825688
rs999889
rs10811871
rs10795422
rs13287131
rs1884389
rs1421334
rs2124499
rs4800191
rs756717
rs11945861
rs7788008
rs8090983
rs4981693
rs7318817
rs294704
rs7871866
rs491711
rs200810
rs818524
rs12150665
rs10827649
rs6734537
rs6443750
rs10747488
rs429343
rs895330
rs2832283
rs12041258
rs2235564
rs17014375
rs6561943
rs765875
rs1158805
rs17767510
rs16889835
rs9382285
rs7761673
rs326896
rs4722398
rs13329567
rs12022461
rs16822990
rs10741329
rs4952843
rs3754963
rs7206608
rs4151664
rs7970953
rs2608703
rs331966
rs9408882
rs2605603
rs1937683
rs7117238
rs7983065
rs6593688
rs902695
rs13069244
rs1436344
rs10867256
rs946824
rs17710386
rs7037266
rs9547153
rs1266874
rs3844598
rs9367368
rs1445652
rs2196618
rs1409818
rs13250058
rs962273
rs11615578
rs774246
rs17056301
rs4968656
rs9806742
rs7599312
rs1927790
rs4851029
rs7196720
rs7598508
rs7239575
rs12675063
rs6556301
rs1535660
rs11496125
rs4414033
rs12422552
rs10971709
rs3736485
rs11173522
rs12334877
rs12680842
rs2429150
rs9571687
rs11170468
rs12328930
rs6841761
rs10518694
rs10733051
rs7535528
rs2357760
rs3772882
rs13110266
rs12888955
rs901630
rs13147390
rs10269783
rs1982725
rs4358081
rs3007105
rs2065418
rs17535082
rs8181823
rs1452075
rs977747
rs3764835
rs2836964
rs4936175
rs11538
rs4864201
rs12629015
rs13191362
rs1982441
rs6712
rs17405819
rs12718572
rs2051559
rs4518345
rs11889536
rs12922346
rs1327259
rs10408324
rs12593036
rs10858334
rs8071182
rs427943
rs1896767
rs1285997
rs10169594
rs11908637
rs1707322
rs8097672
rs12981256
rs7715256
rs1656377
rs9615905
rs11084553
rs11251352
rs17113297
rs287104
rs6591407
rs4820408
rs12652212
rs7024334
rs11781699
rs3904244
rs7694732
rs175165
rs4842491
rs1492767
rs9783858
rs4148155
rs10478110
rs930295
rs10211055
rs2361988
rs7730004
rs9927848
rs7594289
rs784944
rs9688431
rs4954638
rs1891216
rs12905439
rs16871902
rs1472169
rs1064213
rs1150659
rs12936083
rs754635
rs380857
rs17663412
rs2600226
rs2875762
rs7925214
rs17311369
rs2903971
rs17446257
rs11856579
rs11030618
rs2423668
rs1784460
rs2744974
rs12429545
rs10962550
rs208015
rs12932660
rs2943465
rs1321432
rs1241986
rs12364470
rs7222349
rs12098284
rs2367112
rs17425707
rs10742752
rs2543132
rs8036040
rs6785245
rs17207196
rs2791653
rs7334078
rs872281
rs10920678
rs4937870
rs339991
rs10182181
rs8123881
rs7826312
rs3935648
rs273504
rs7615297
rs2143253
rs9845966
rs7933205
rs3807645
rs8065336
rs6235
rs592483
rs9284814
rs1218822
rs11611246
rs4307239
rs852056
rs1993709
rs10192119
rs1477199
rs1528435
rs12602912
rs17551974
rs1465900
rs7102454
rs12933482
rs4858193
rs1948080
rs17499593
rs6500208
rs7780752
rs845084
rs11736228
rs4783830
rs9375702
rs9650755
rs40067
rs1187352
rs6461115
rs215634
rs2281819
rs1268065
rs10968114
rs1503526
rs1928295
rs6512302
rs7172627
rs1836303
rs6050446
rs9300422
rs9304665
rs7683836
rs11115176
rs11150911
rs1112613
rs10750215
rs4906908
rs2283093
rs7637852
rs11739877
rs1430387
rs17001561
rs12564992
rs6804842
rs12762034
rs11185111
rs6985109
rs16849710
rs13184896
rs4072917
rs4516268
rs7488867
rs3749897
rs4740619
rs559231
rs10248136
rs3800229
rs33500
rs9951619
rs12939549
rs1830074
rs17636031
rs6587552
rs947612
rs1579557
rs879620
rs9989141
rs7724675
rs12448257
rs1188017
rs4639527
rs6692586
rs1075901
rs9816226
rs17789218
rs11773362
rs2425840
rs17203016
rs7640424
rs2694047
rs17238110
rs7557796
rs11165643
rs7551507
rs987237
rs4671328
rs7498665
rs7550711
rs6265
All
0.00
0.25
0.50
0.75
MR leave-one-out sensitivity analysis for
'body mass index || id:ieu-b-40' on 'Pilonidal cyst || id:finngen_R12_L12_PILONIDALCYST'
