# Supplementary material for: Risk factors for sacrococcygeal pilonidal sinus: a systematic review and meta-analysis supplemented by genetic causal assessment
Source: Front Surg. 2026 Jan 7;12:1718589. doi: 10.3389/fsurg.2025.1718589 (PMC12819706; doi:10.3389/fsurg.2025.1718589)
Supplement: Supplementary file 2 [file Datasheet2.zip › Supplementary Data 2/MR_pipeline_after_confounding_SNPs_removal/ukb-b-5192_finngen_R12_L12_PILONIDALCYST_20250627000040/02. finngen_R12_L12_PILONIDALCYST_forest_plot.pptx]

## Slide 1
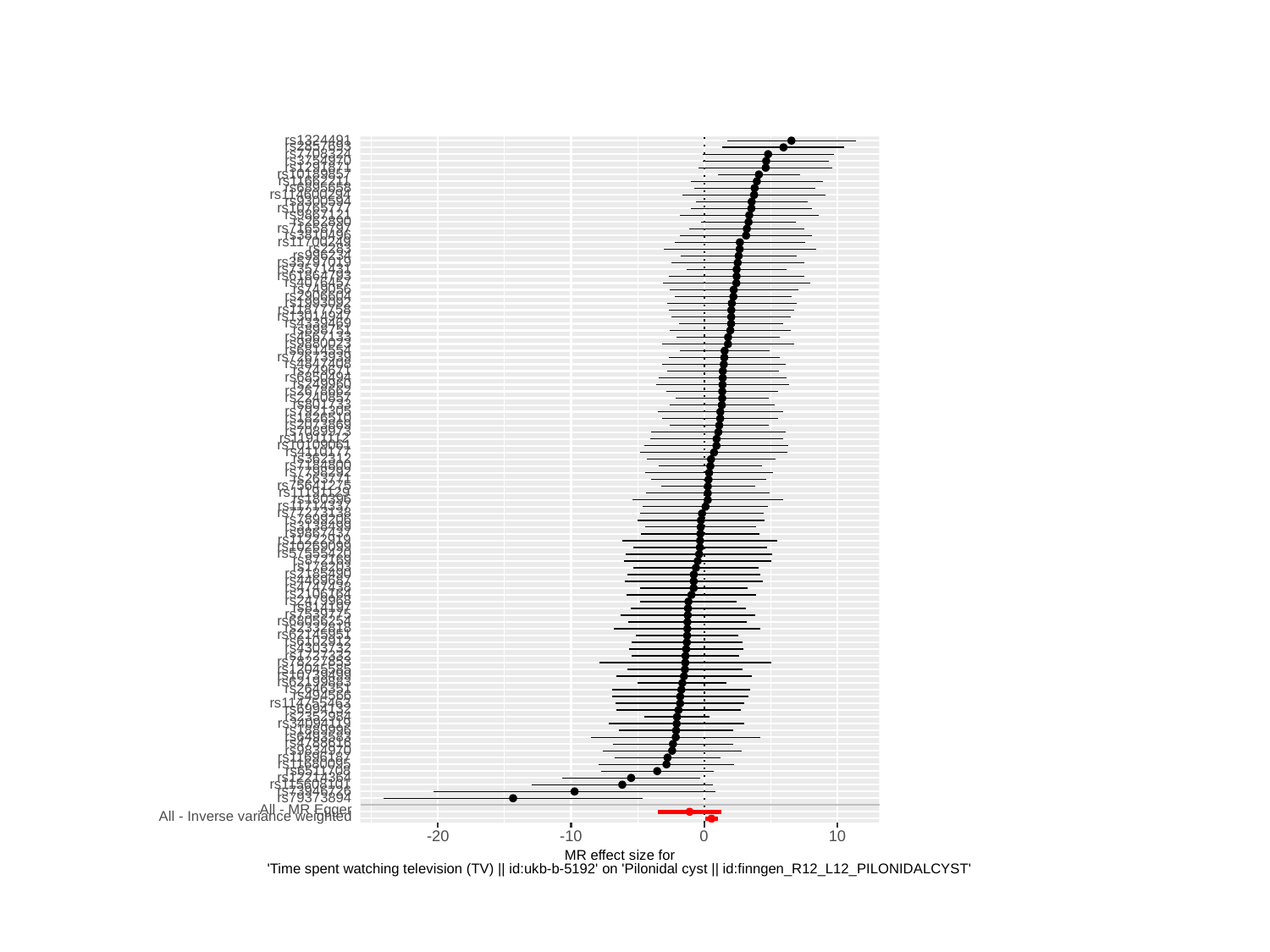

#
rs1324491
rs2857693
rs7708324
rs3754970
rs1291871
rs10189857
rs11662211
rs6895658
rs114600294
rs9300594
rs10765777
rs9867121
rs262890
rs71658797
rs3810496
rs11700249
rs2283
rs996234
rs35797019
rs73571431
rs61864793
rs4076457
rs749056
rs2906604
rs1993092
rs11877758
rs13014947
rs4339469
rs898751
rs4567133
rs9880023
rs6814554
rs72673939
rs4847408
rs749671
rs6850494
rs249960
rs2678662
rs2240857
rs801733
rs7921305
rs1826510
rs2073869
rs7089973
rs11911112
rs10109061
rs4110177
rs362312
rs7184800
rs7798292
rs263771
rs75641275
rs11191129
rs180396
rs11714337
rs77273138
rs7899206
rs3138499
rs9867437
rs11222919
rs10269099
rs57555420
rs872169
rs178203
rs2185490
rs4469687
rs4747438
rs2106164
rs2479968
rs814197
rs7539775
rs68056254
rs2332818
rs62145951
rs6102912
rs4303732
rs1727332
rs78227853
rs12045585
rs10739499
rs62199883
rs2646351
rs494566
rs114755463
rs6994132
rs2352984
rs34094119
rs1889996
rs6493583
rs4788616
rs9834970
rs11696187
rs11680095
rs6511708
rs12214364
rs115608101
rs73946726
rs79373894
All - MR Egger
All - Inverse variance weighted
-20
-10
0
10
MR effect size for
'Time spent watching television (TV) || id:ukb-b-5192' on 'Pilonidal cyst || id:finngen_R12_L12_PILONIDALCYST'
