# Supplementary material for: Risk factors for sacrococcygeal pilonidal sinus: a systematic review and meta-analysis supplemented by genetic causal assessment
Source: Front Surg. 2026 Jan 7;12:1718589. doi: 10.3389/fsurg.2025.1718589 (PMC12819706; doi:10.3389/fsurg.2025.1718589)
Supplement: Supplementary file 2 [file Datasheet2.zip › Supplementary Data 2/MR_pipeline_after_confounding_SNPs_removal/ukb-b-5192_finngen_R12_L12_PILONIDALCYST_20250627000040/03. finngen_R12_L12_PILONIDALCYST_leaveone_plot.pptx]

## Slide 1
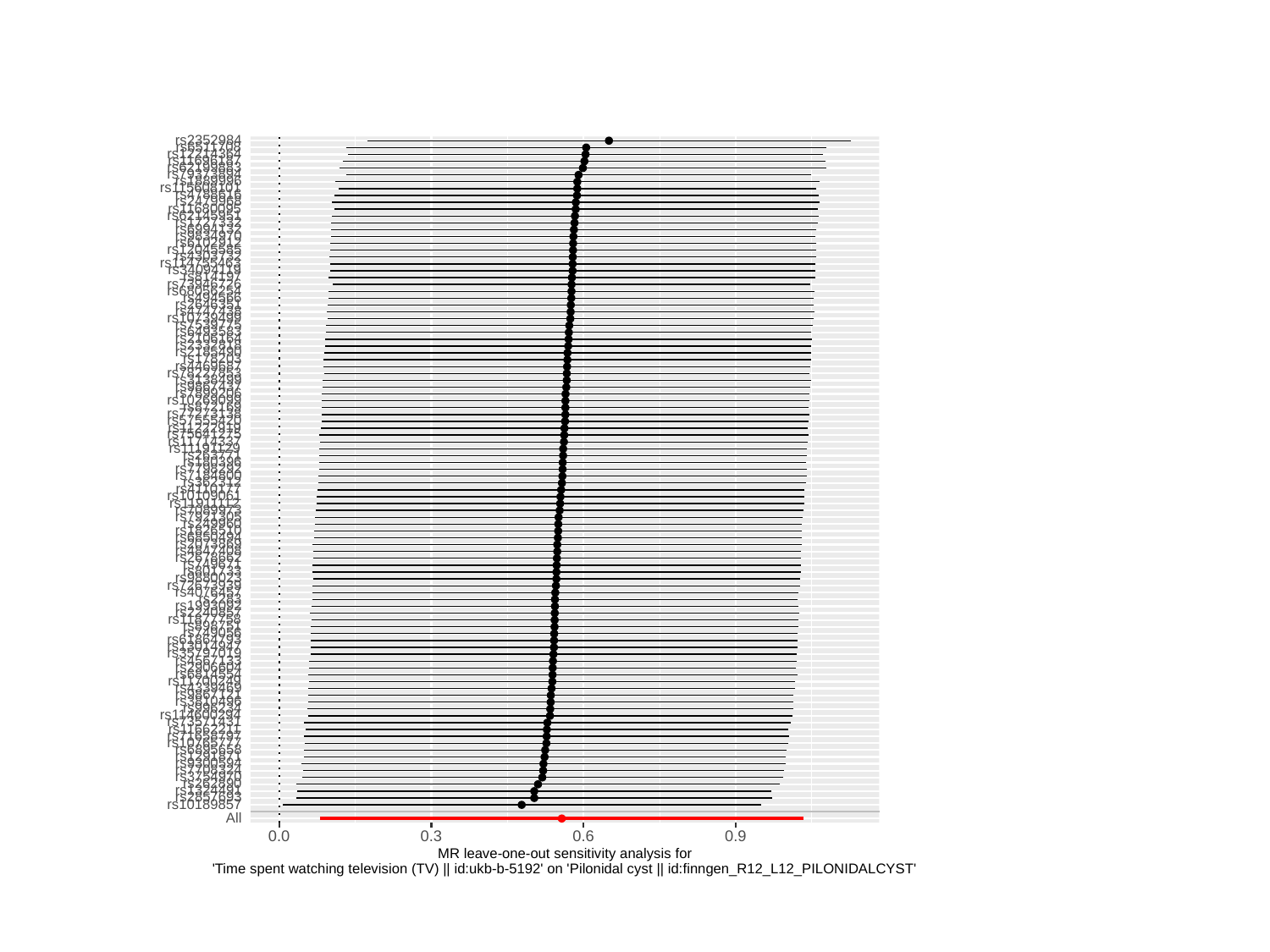

#
rs2352984
rs6511708
rs12214364
rs11696187
rs62199883
rs79373894
rs1889996
rs115608101
rs4788616
rs2479968
rs11680095
rs62145951
rs1727332
rs6994132
rs9834970
rs6102912
rs12045585
rs4303732
rs114755463
rs34094119
rs814197
rs73946726
rs68056254
rs494566
rs2646351
rs4747438
rs10739499
rs7539775
rs6493583
rs2106164
rs2332818
rs2185490
rs178203
rs4469687
rs78227853
rs3138499
rs9867437
rs7899206
rs10269099
rs872169
rs77273138
rs57555420
rs11222919
rs75641275
rs11714337
rs11191129
rs263771
rs180396
rs7798292
rs7184800
rs362312
rs4110177
rs10109061
rs11911112
rs7089973
rs7921305
rs249960
rs1826510
rs6850494
rs2073869
rs4847408
rs2678662
rs749671
rs801733
rs9880023
rs72673939
rs4076457
rs2283
rs1993092
rs2240857
rs11877758
rs898751
rs749056
rs61864793
rs13014947
rs35797019
rs4567133
rs2906604
rs6814554
rs11700249
rs4339469
rs9867121
rs3810496
rs996234
rs114600294
rs73571431
rs11662211
rs71658797
rs10765777
rs6895658
rs1291871
rs9300594
rs7708324
rs3754970
rs262890
rs1324491
rs2857693
rs10189857
All
0.3
0.0
0.6
0.9
MR leave-one-out sensitivity analysis for
'Time spent watching television (TV) || id:ukb-b-5192' on 'Pilonidal cyst || id:finngen_R12_L12_PILONIDALCYST'
