# Supplementary material for: Risk factors for sacrococcygeal pilonidal sinus: a systematic review and meta-analysis supplemented by genetic causal assessment
Source: Front Surg. 2026 Jan 7;12:1718589. doi: 10.3389/fsurg.2025.1718589 (PMC12819706; doi:10.3389/fsurg.2025.1718589)
Supplement: Supplementary file 2 [file Datasheet2.zip › Supplementary Data 2/MR_pipeline_p5e-6/finn-b-R18_HYPERHIDROSIS_finngen_R12_L12_PILONIDALCYST_20251109223616/02. finngen_R12_L12_PILONIDALCYST_forest_plot.pptx]

## Slide 1
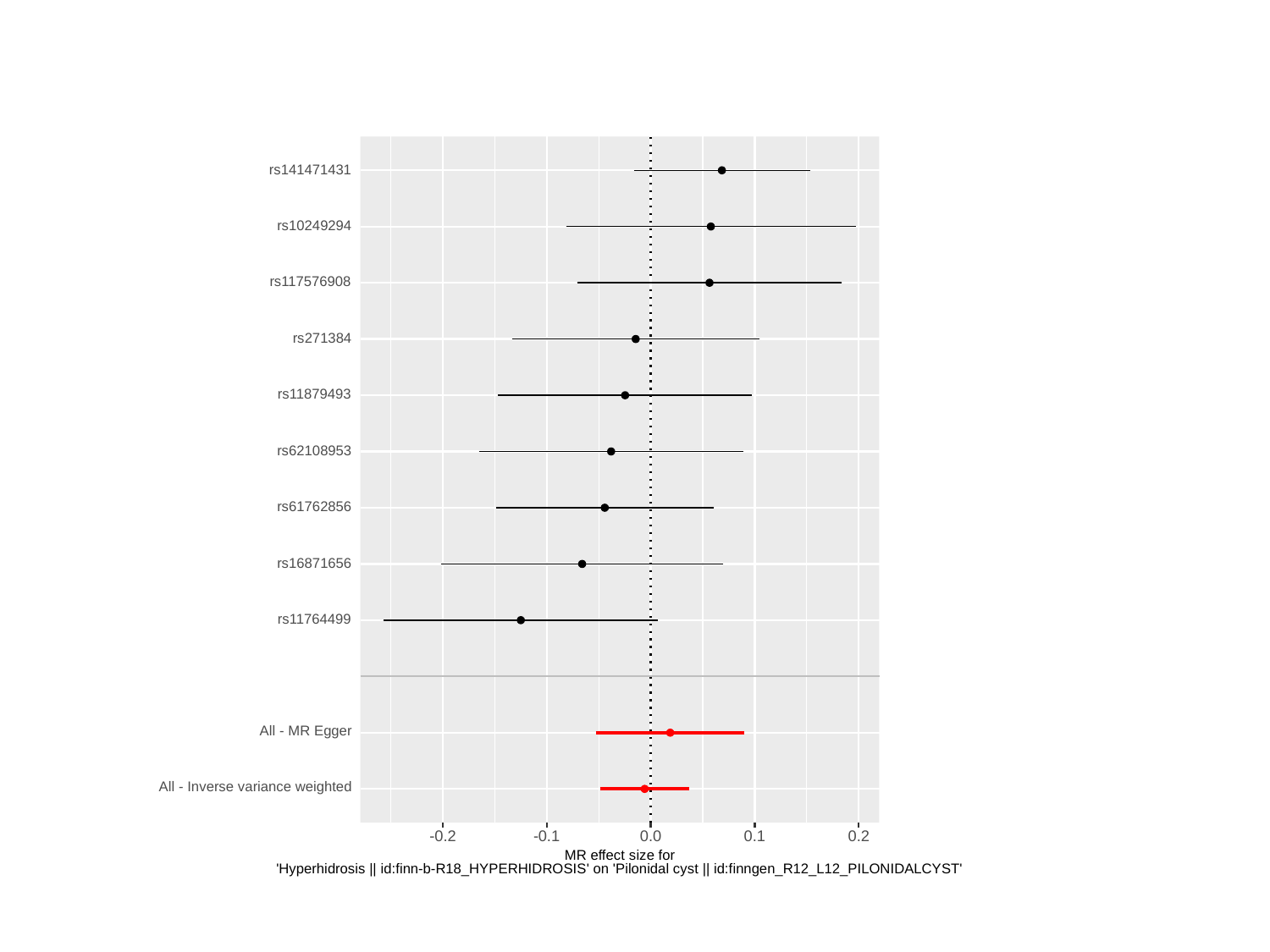

#
rs141471431
rs10249294
rs117576908
rs271384
rs11879493
rs62108953
rs61762856
rs16871656
rs11764499
All - MR Egger
All - Inverse variance weighted
-0.2
-0.1
0.0
0.1
0.2
MR effect size for
'Hyperhidrosis || id:finn-b-R18_HYPERHIDROSIS' on 'Pilonidal cyst || id:finngen_R12_L12_PILONIDALCYST'
