# Supplementary material for: Risk factors for sacrococcygeal pilonidal sinus: a systematic review and meta-analysis supplemented by genetic causal assessment
Source: Front Surg. 2026 Jan 7;12:1718589. doi: 10.3389/fsurg.2025.1718589 (PMC12819706; doi:10.3389/fsurg.2025.1718589)
Supplement: Supplementary file 2 [file Datasheet2.zip › Supplementary Data 2/MR_pipeline_p5e-6/forest_plots/finngen_R12_L12_HIDRADENITISSUP.finngen_R12_L12_PILONIDALCYST.pdf]

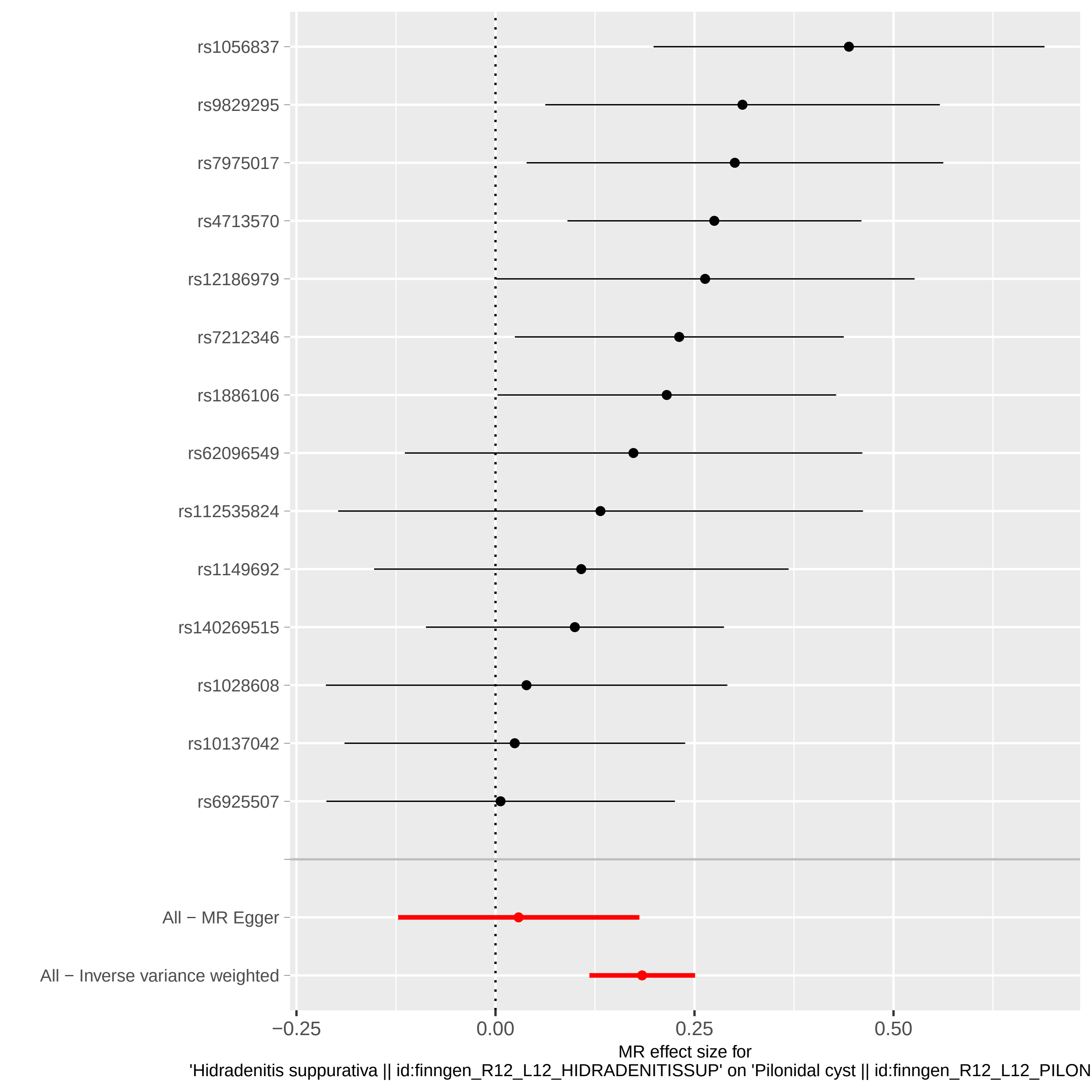
rs1056837
rs9829295
rs7975017
rs4713570
rs12186979
rs7212346
rs1886106
rs62096549
rs112535824
rs1149692
rs140269515
rs1028608
rs10137042
rs6925507
All − MR Egger
All − Inverse variance weighted
−0.25
0.00
0.25
0.50
MR effect size for
'Hidradenitis suppurativa || id:finngen_R12_L12_HIDRADENITISSUP' on 'Pilonidal cyst || id:finngen_R12_L12_PILON
