# Supplementary figures and images for: Risk factors for sacrococcygeal pilonidal sinus: a systematic review and meta-analysis supplemented by genetic causal assessment
Source: Front Surg. 2026 Jan 7;12:1718589. doi: 10.3389/fsurg.2025.1718589 (PMC12819706; doi:10.3389/fsurg.2025.1718589)

## Slide 1
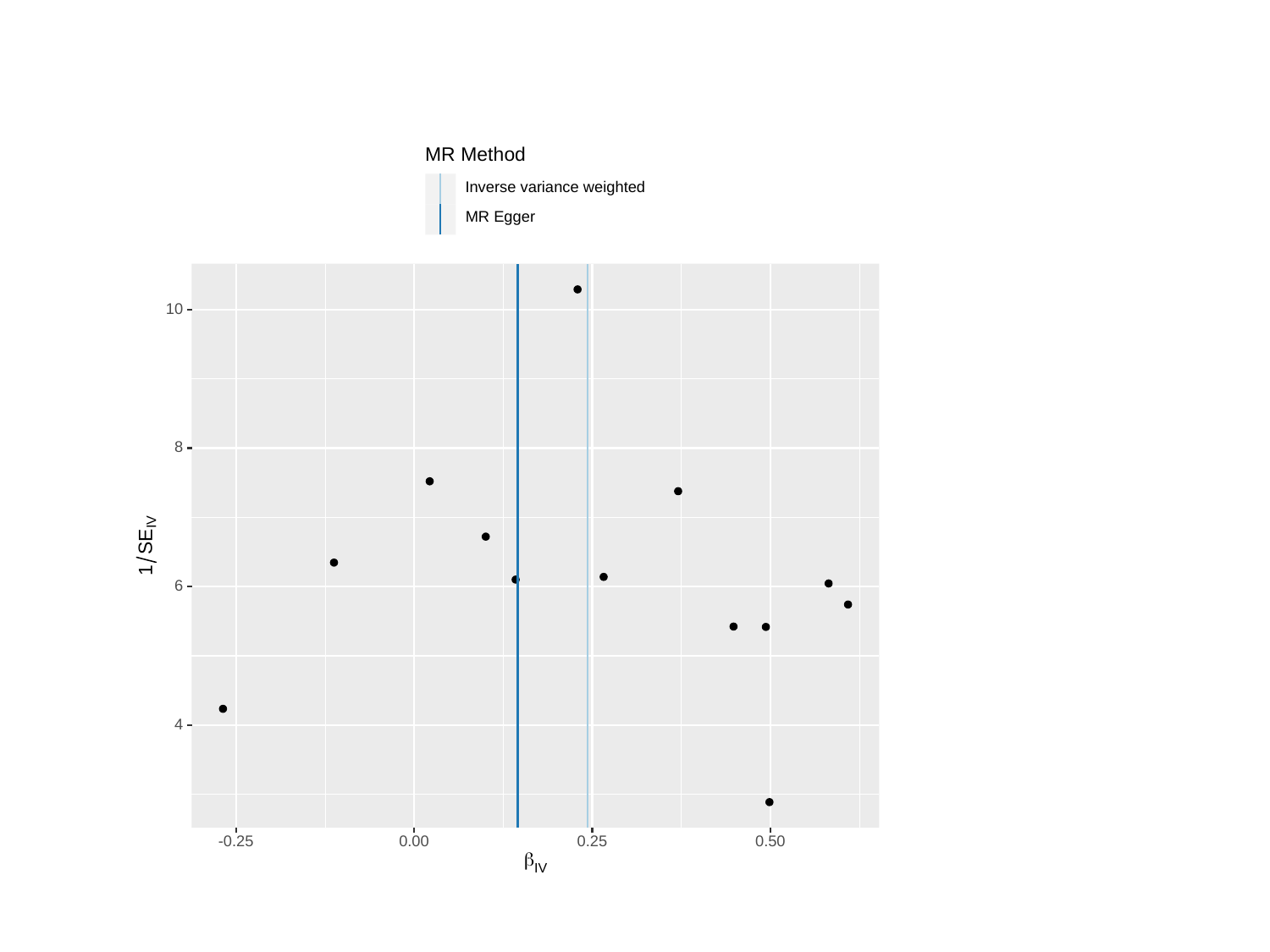

#
MR Method
Inverse variance weighted
MR Egger
10
8
V
I
E
S
1
6
4
-0.25
0.00
0.25
0.50
β
I
V

Supplement: Supplementary file 2 [file Datasheet2.zip › Supplementary Data 2/MR_pipeline_after_confounding_SNPs_removal/ebi-a-GCST006095_finngen_R12_L12_PILONIDALCYST_20250626220939/04. finngen_R12_L12_PILONIDALCYST_funnel_plot.pptx]

## Slide 1
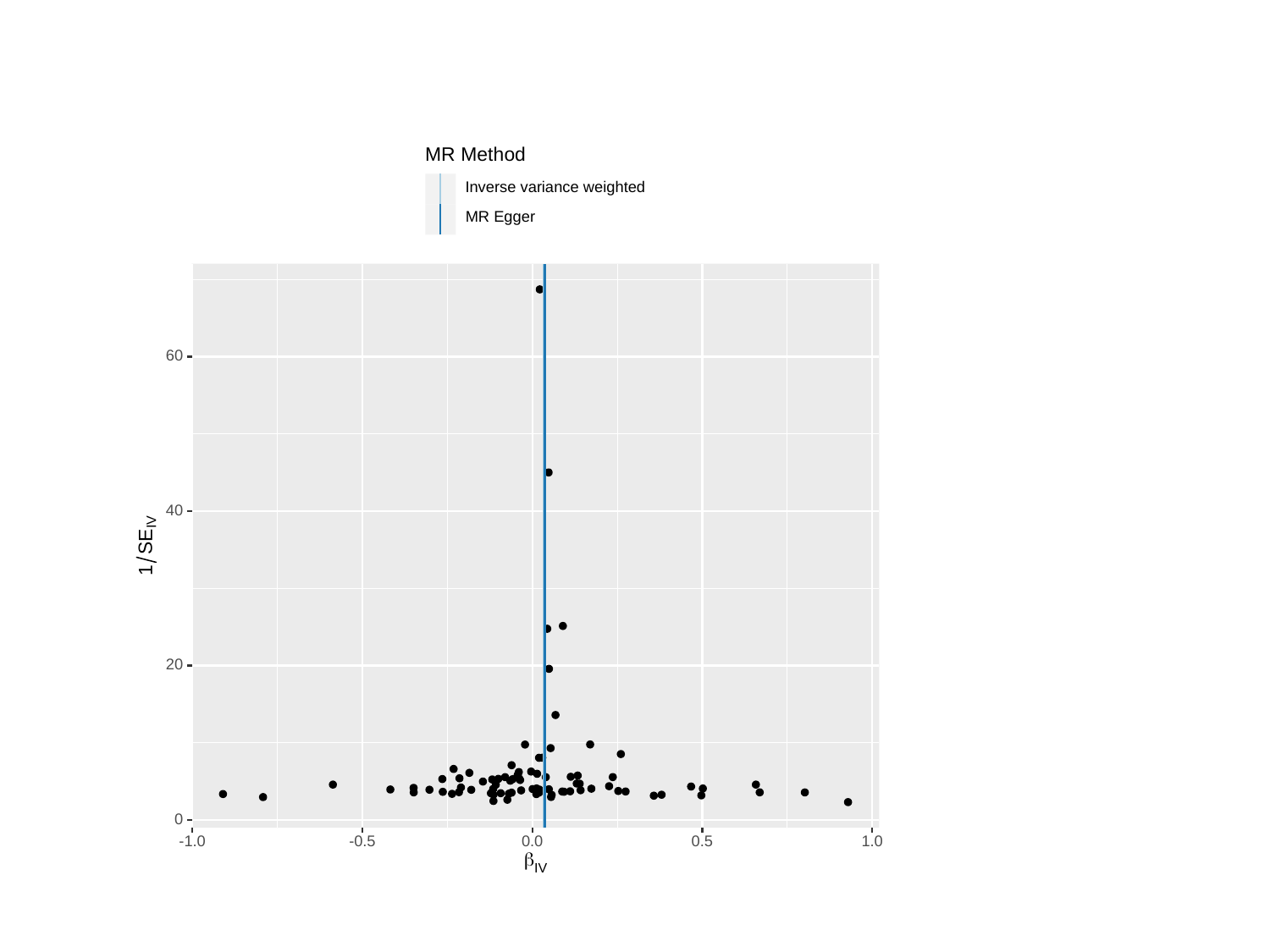

#
MR Method
Inverse variance weighted
MR Egger
60
40
V
I
E
S
1
20
0
-1.0
-0.5
0.0
0.5
1.0
β
I
V

Supplement: Supplementary file 2 [file Datasheet2.zip › Supplementary Data 2/MR_pipeline_after_confounding_SNPs_removal/ebi-a-GCST90014023_finngen_R12_L12_PILONIDALCYST_20250627002938/04. finngen_R12_L12_PILONIDALCYST_funnel_plot.pptx]

## Slide 1
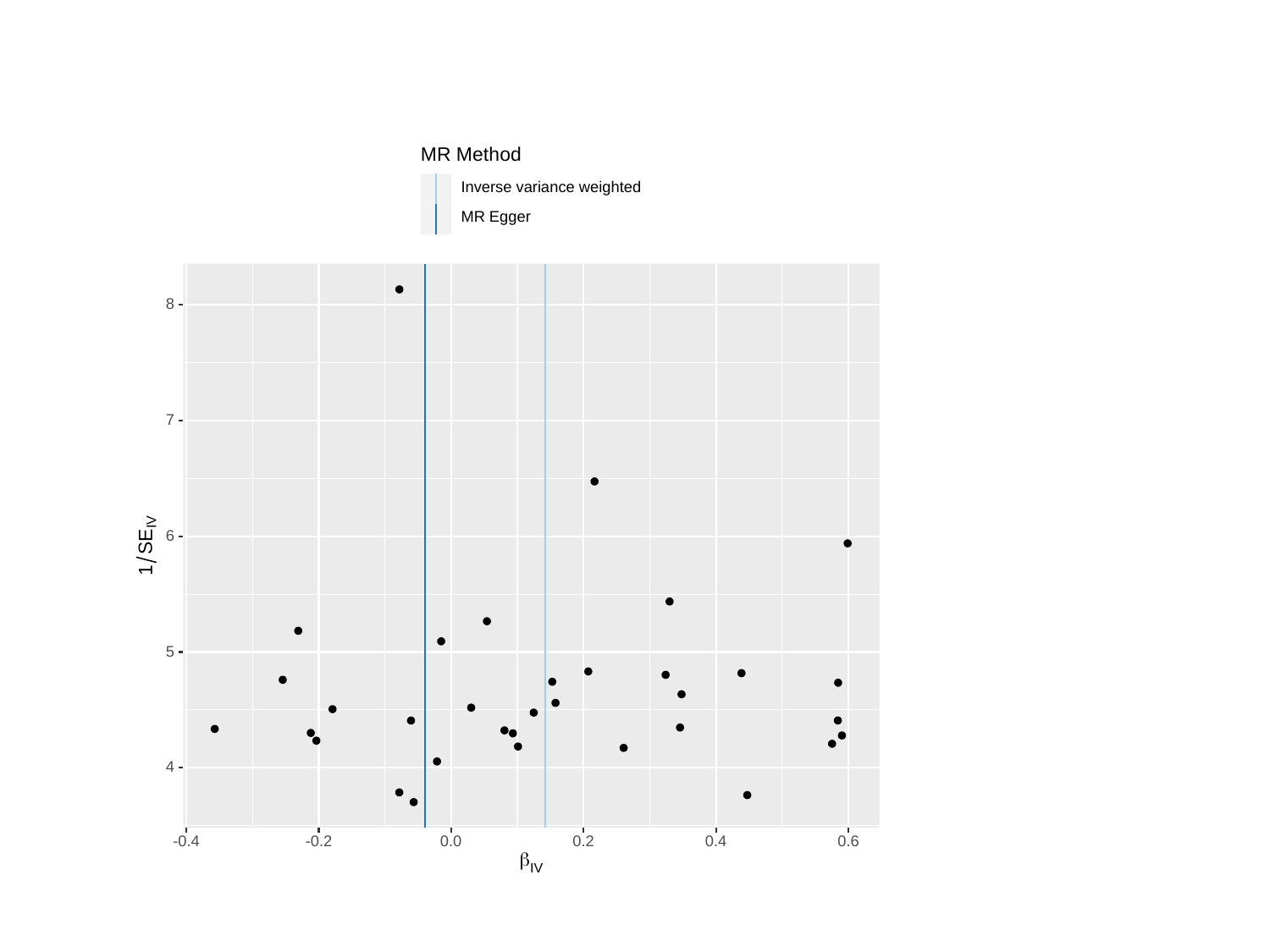

#
MR Method
Inverse variance weighted
MR Egger
8
7
V
I
E
6
S
1
5
4
-0.4
-0.2
0.0
0.2
0.4
0.6
β
I
V

Supplement: Supplementary file 2 [file Datasheet2.zip › Supplementary Data 2/MR_pipeline_after_confounding_SNPs_removal/finngen_R12_L12_ACNE_finngen_R12_L12_PILONIDALCYST_20251109185740/04. finngen_R12_L12_PILONIDALCYST_funnel_plot.pptx]

## Slide 1
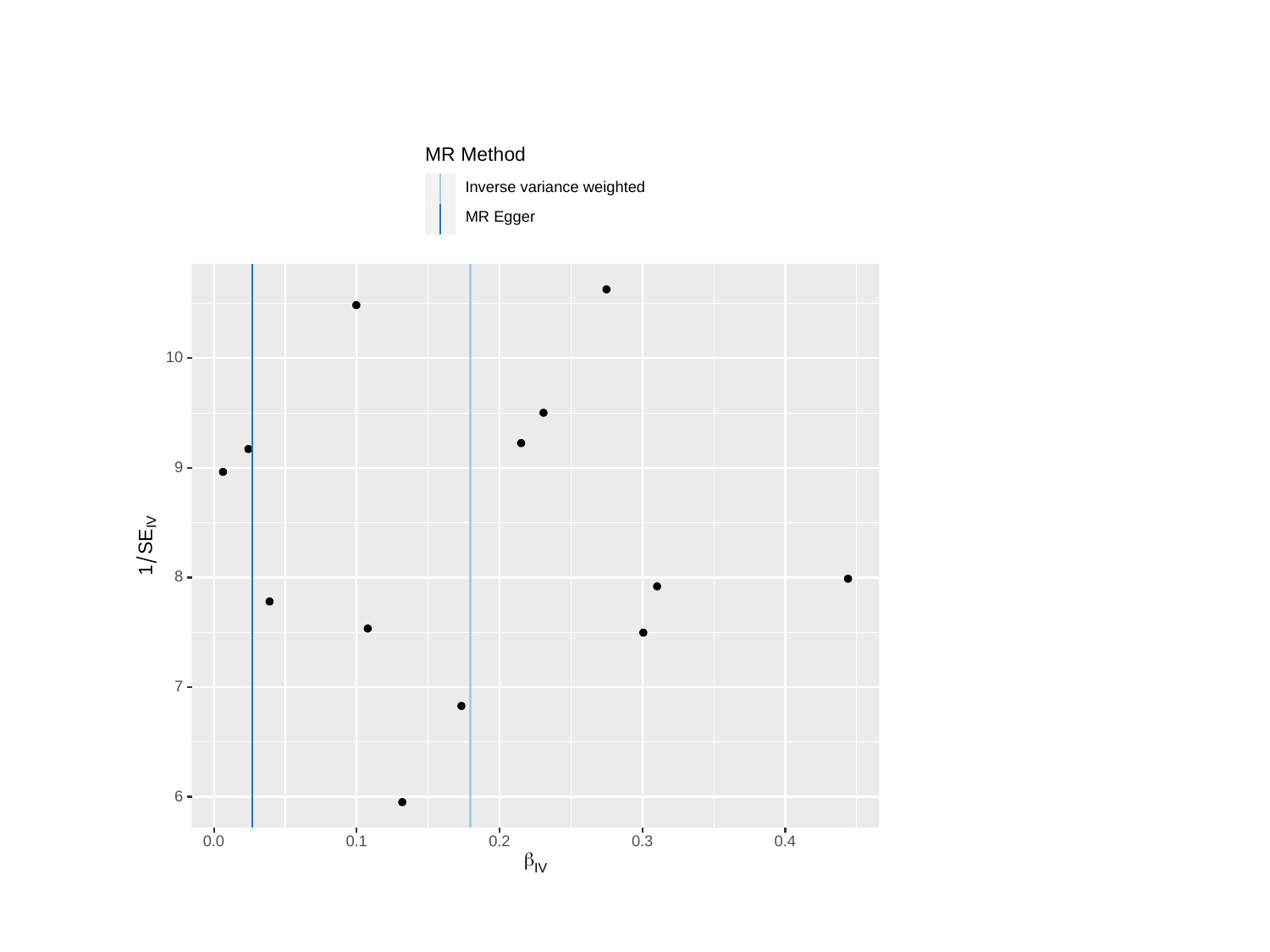

#
MR Method
Inverse variance weighted
MR Egger
10
9
V
I
E
S
1
8
7
6
0.3
0.0
0.1
0.2
0.4
β
I
V

Supplement: Supplementary file 2 [file Datasheet2.zip › Supplementary Data 2/MR_pipeline_after_confounding_SNPs_removal/finngen_R12_L12_HIDRADENITISSUP_finngen_R12_L12_PILONIDALCYST_20250626221908/04. finngen_R12_L12_PILONIDALCYST_funnel_plot.pptx]

## Slide 1
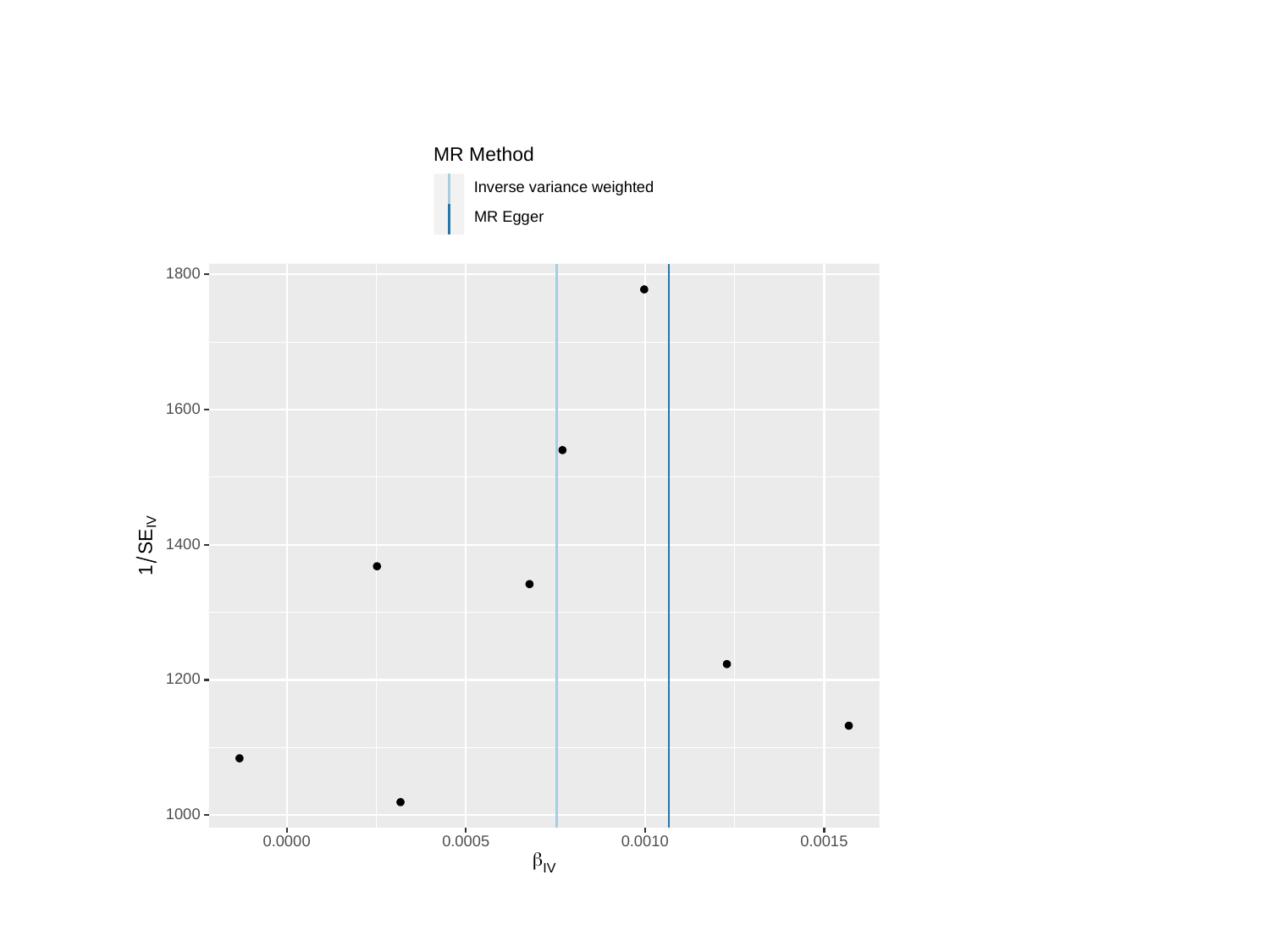

#
MR Method
Inverse variance weighted
MR Egger
1800
1600
V
I
E
1400
S
1
1200
1000
0.0000
0.0005
0.0010
0.0015
β
I
V

Supplement: Supplementary file 2 [file Datasheet2.zip › Supplementary Data 2/MR_pipeline_after_confounding_SNPs_removal/finngen_R12_L12_HIDRADENITISSUP_ukb-b-5617/04. ukb-b-5617_funnel_plot.pptx]

## Slide 1
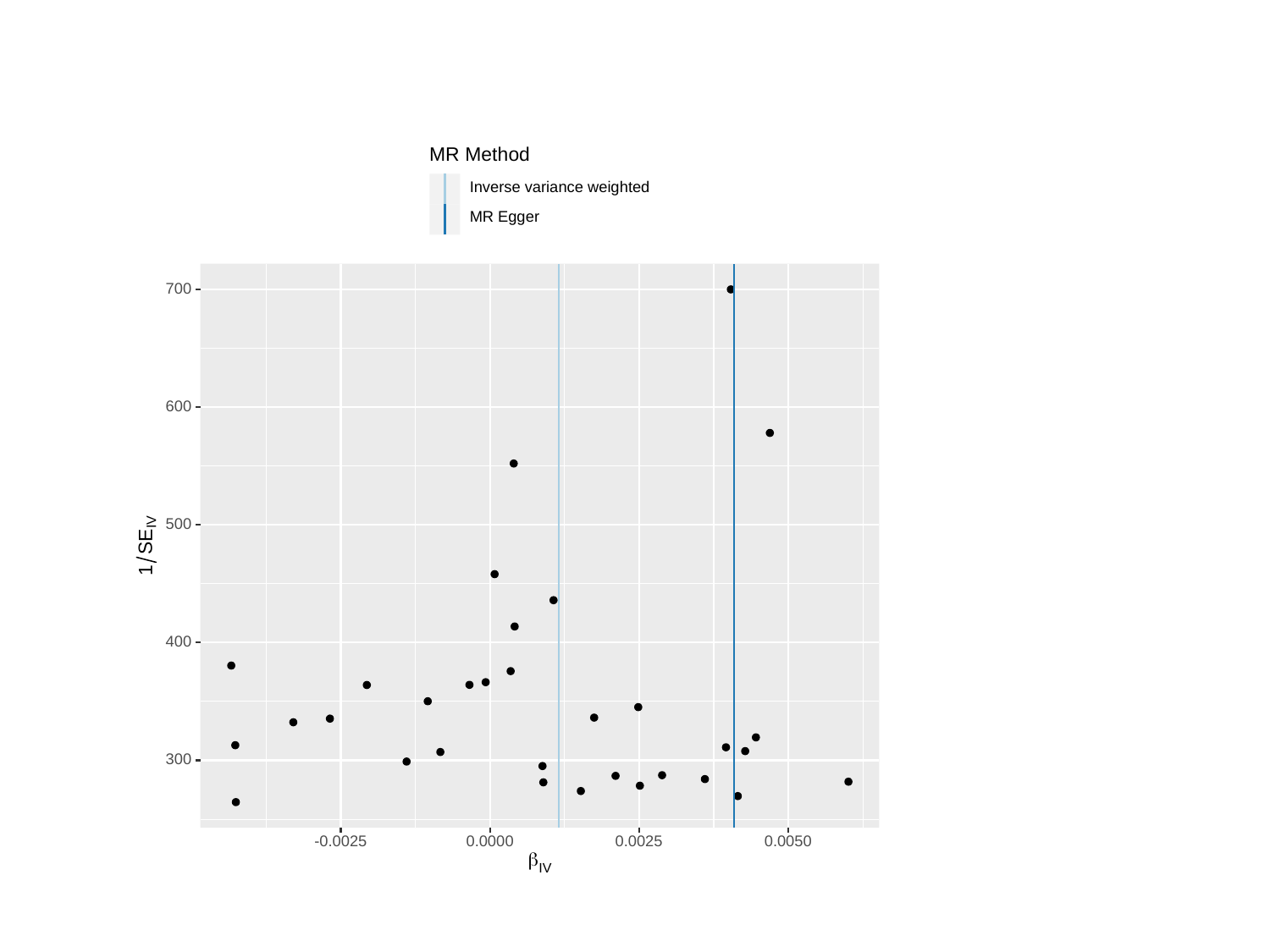

#
MR Method
Inverse variance weighted
MR Egger
700
600
V
500
I
E
S
1
400
300
-0.0025
0.0000
0.0025
0.0050
β
I
V

Supplement: Supplementary file 2 [file Datasheet2.zip › Supplementary Data 2/MR_pipeline_after_confounding_SNPs_removal/GCST90245818_ukb-b-5617_20251109205253/04. ukb-b-5617_funnel_plot.pptx]

## Slide 1
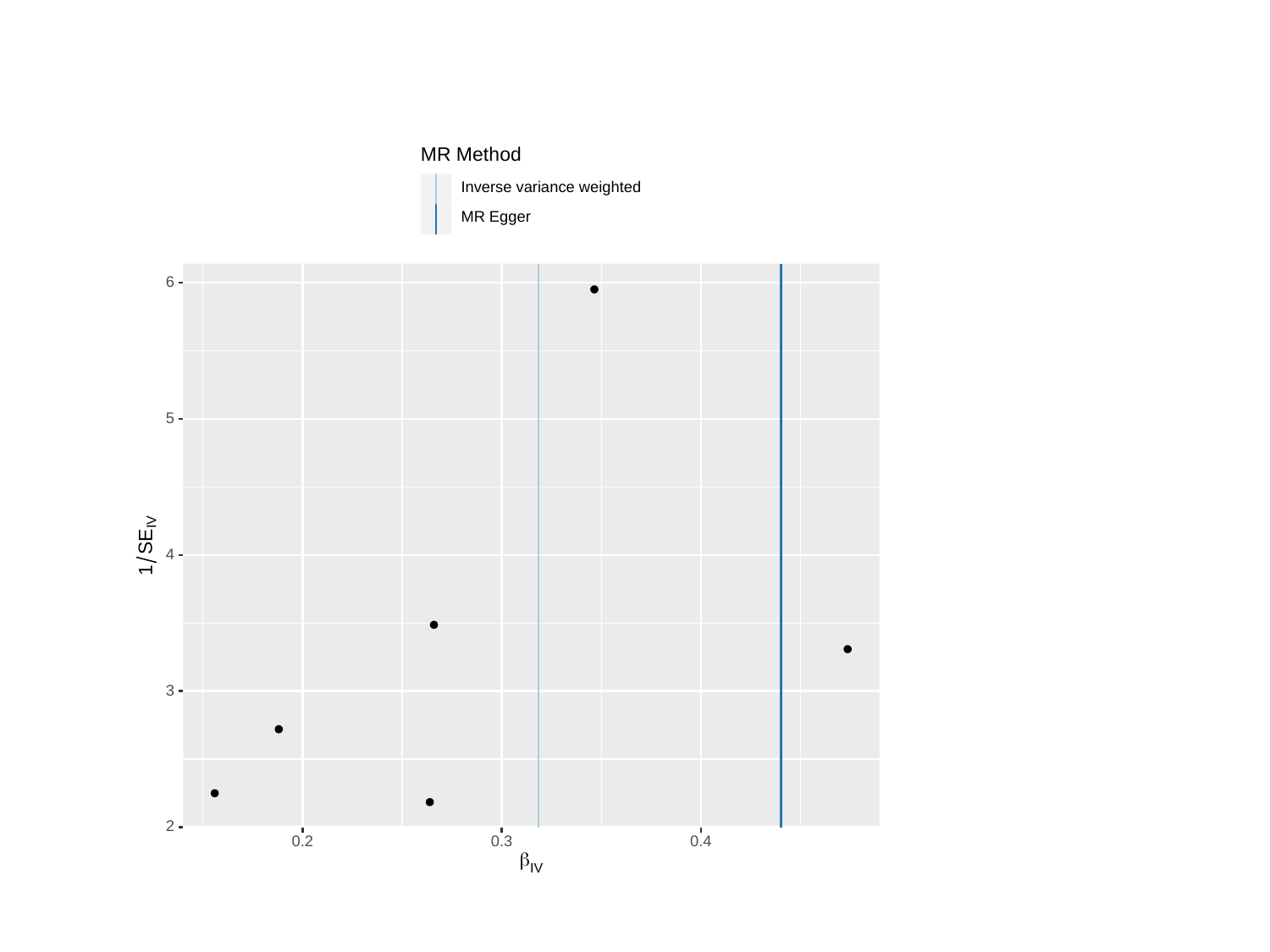

#
MR Method
Inverse variance weighted
MR Egger
6
5
V
I
E
S
4
1
3
2
0.3
0.2
0.4
β
I
V

Supplement: Supplementary file 2 [file Datasheet2.zip › Supplementary Data 2/MR_pipeline_after_confounding_SNPs_removal/ieu-a-93_finngen_R12_L12_PILONIDALCYST_20250626233850/04. finngen_R12_L12_PILONIDALCYST_funnel_plot.pptx]

## Slide 1
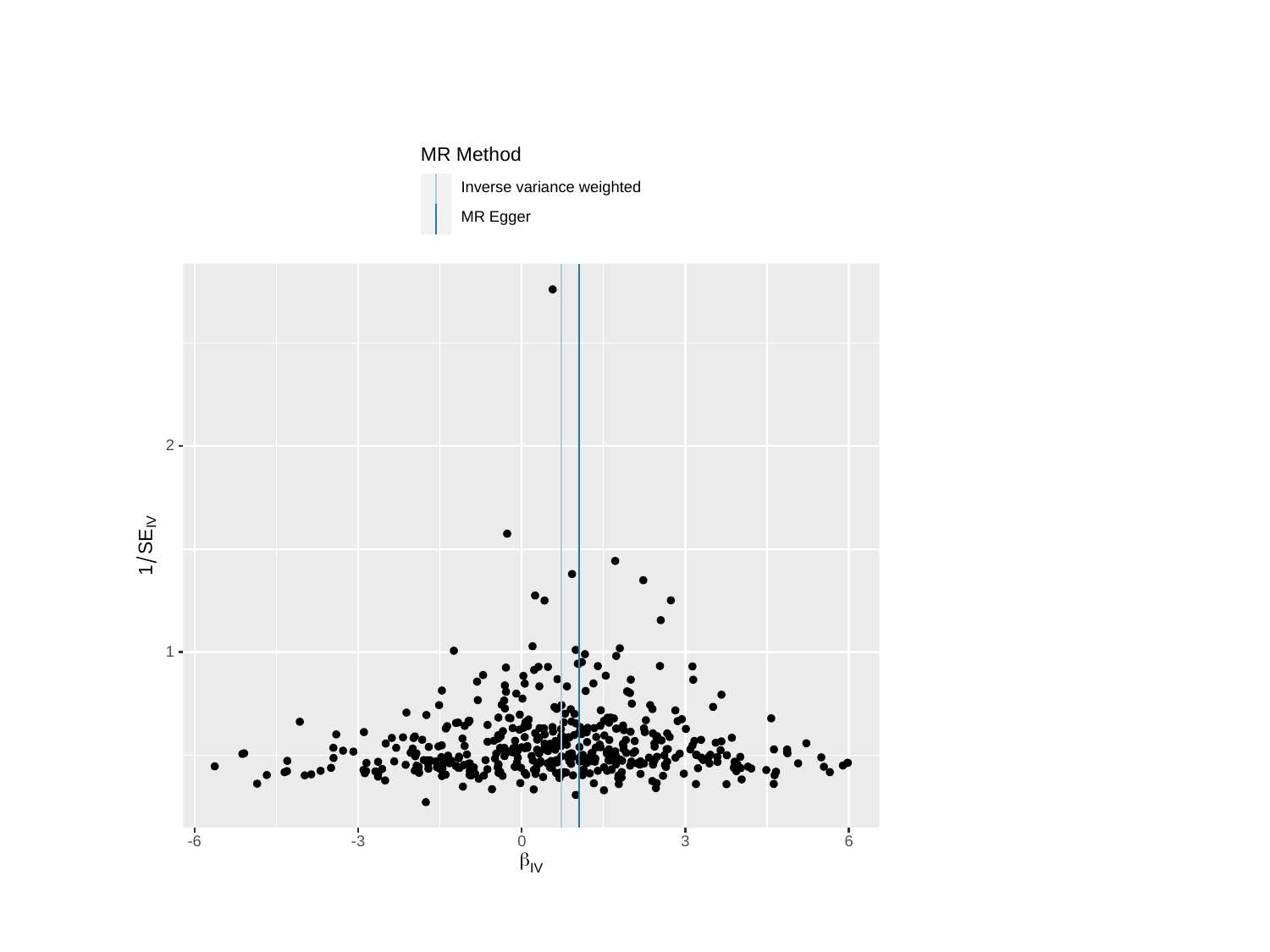

#
MR Method
Inverse variance weighted
MR Egger
2
V
I
E
S
1
1
-3
3
-6
0
6
β
I
V

Supplement: Supplementary file 2 [file Datasheet2.zip › Supplementary Data 2/MR_pipeline_after_confounding_SNPs_removal/ieu-b-40_finngen_R12_L12_PILONIDALCYST_20250626231420/04. finngen_R12_L12_PILONIDALCYST_funnel_plot.pptx]

## Slide 1
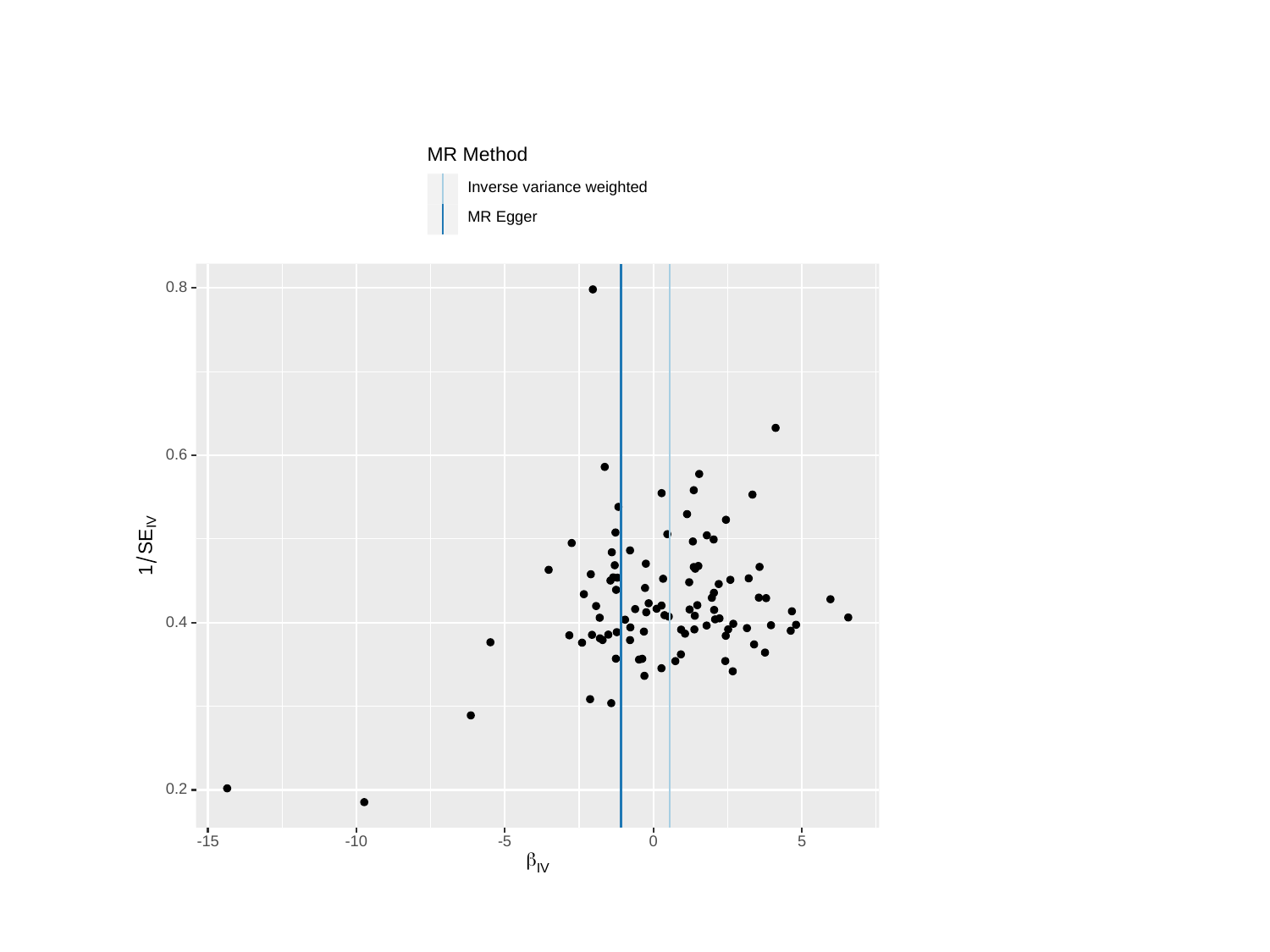

#
MR Method
Inverse variance weighted
MR Egger
0.8
0.6
V
I
E
S
1
0.4
0.2
-15
-10
0
-5
5
β
I
V

Supplement: Supplementary file 2 [file Datasheet2.zip › Supplementary Data 2/MR_pipeline_after_confounding_SNPs_removal/ukb-b-5192_finngen_R12_L12_PILONIDALCYST_20250627000040/04. finngen_R12_L12_PILONIDALCYST_funnel_plot.pptx]

## Slide 1
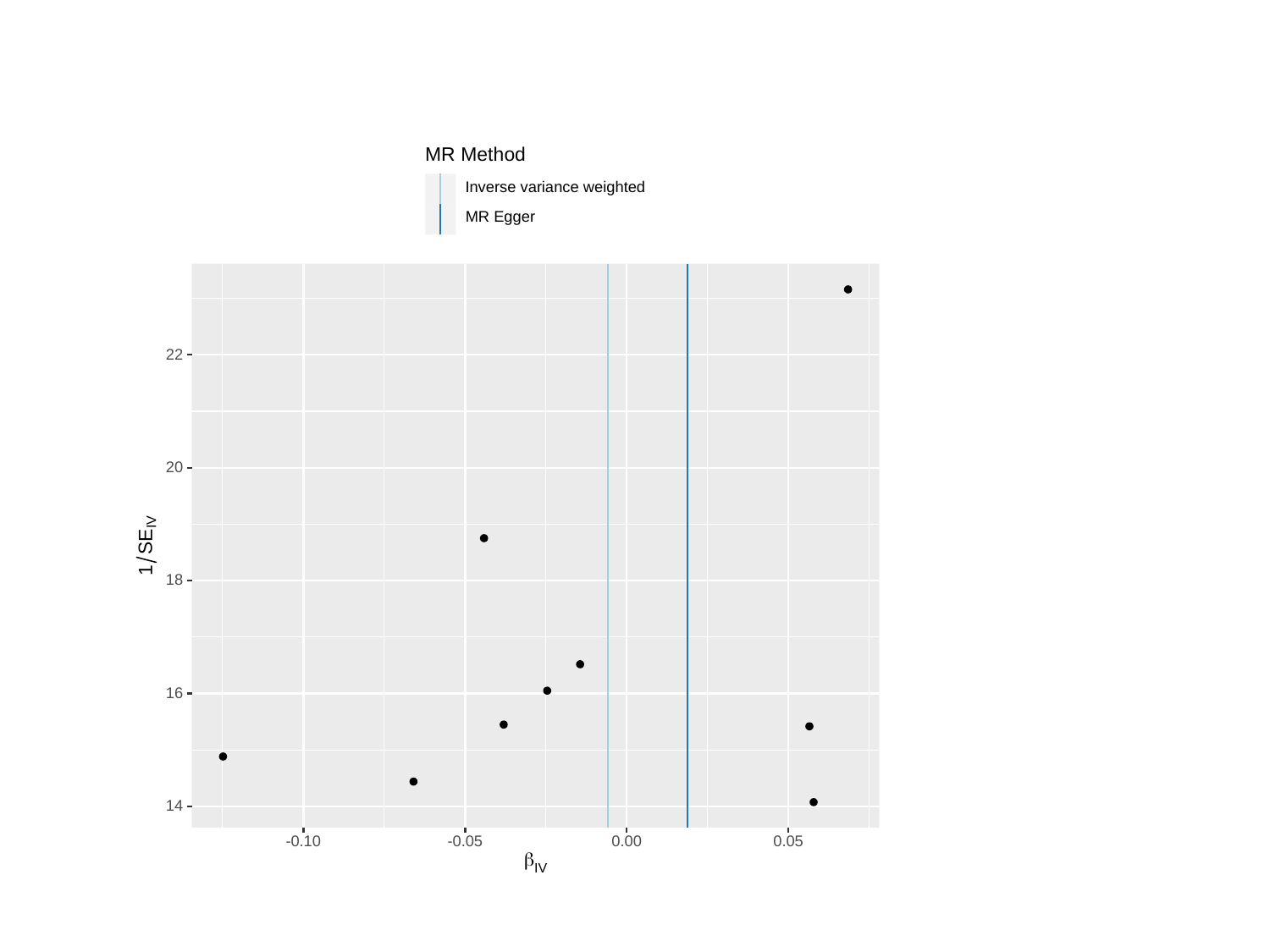

#
MR Method
Inverse variance weighted
MR Egger
22
20
V
I
E
S
1
18
16
14
-0.10
-0.05
0.00
0.05
β
I
V

Supplement: Supplementary file 2 [file Datasheet2.zip › Supplementary Data 2/MR_pipeline_p5e-6/finn-b-R18_HYPERHIDROSIS_finngen_R12_L12_PILONIDALCYST_20251109223616/04. finngen_R12_L12_PILONIDALCYST_funnel_plot.pptx]

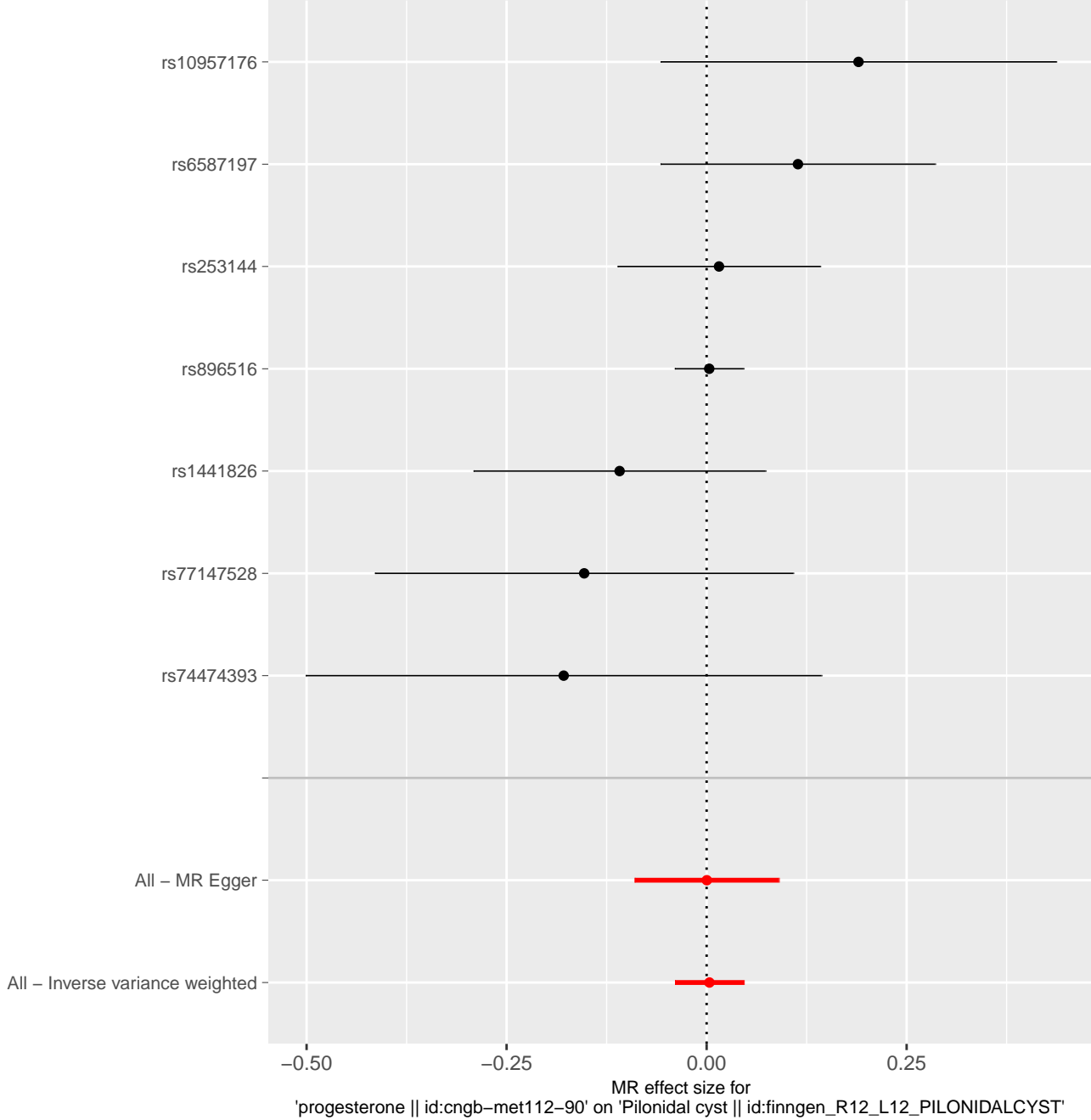

Supplement: Supplementary file 2 [file Datasheet2.zip › Supplementary Data 2/MR_pipeline_p5e-6/forest_plots/cngb-met112-90.finngen_R12_L12_PILONIDALCYST.pdf]

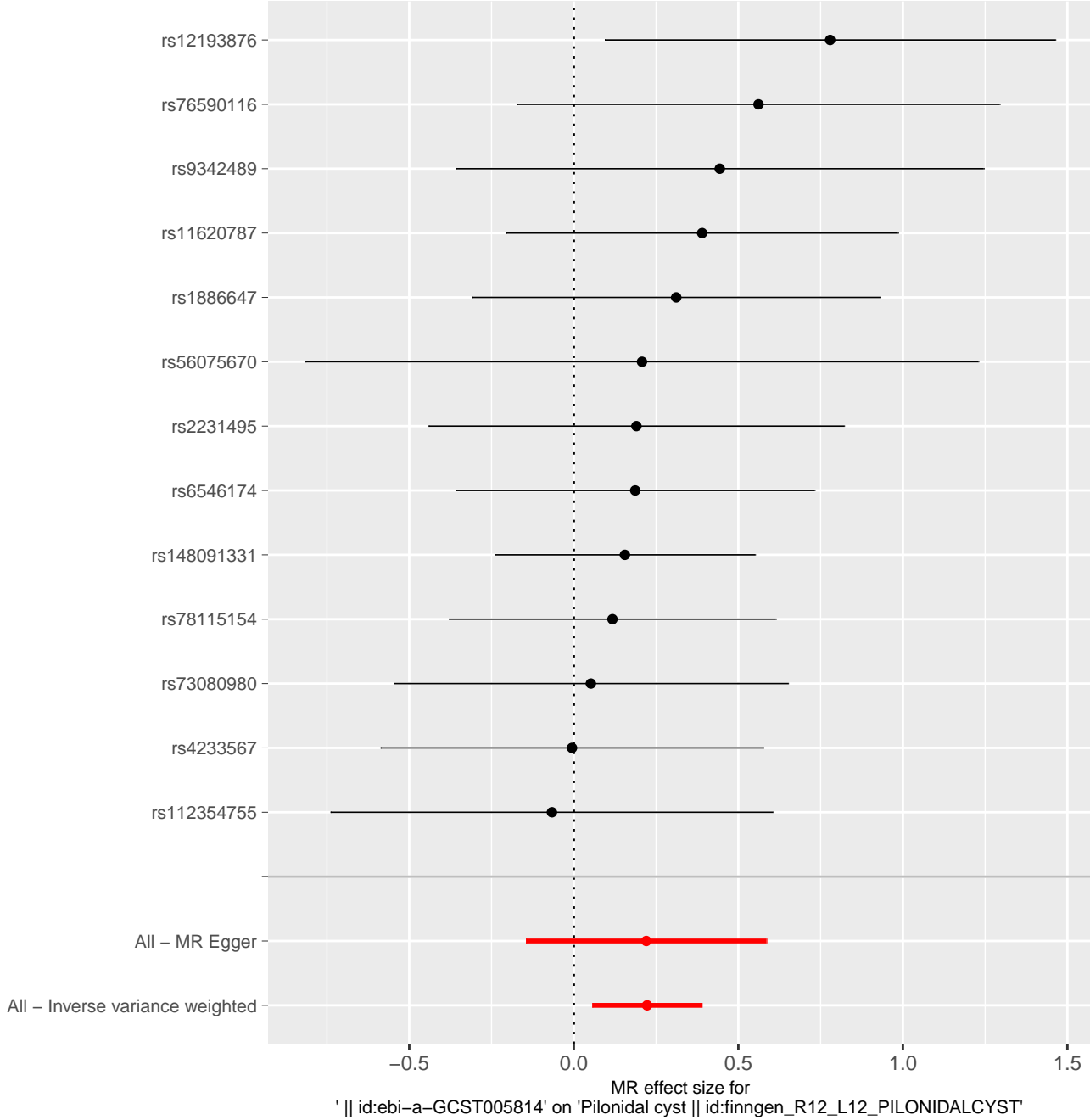

Supplement: Supplementary file 2 [file Datasheet2.zip › Supplementary Data 2/MR_pipeline_p5e-6/forest_plots/ebi-a-GCST005814.finngen_R12_L12_PILONIDALCYST.pdf]

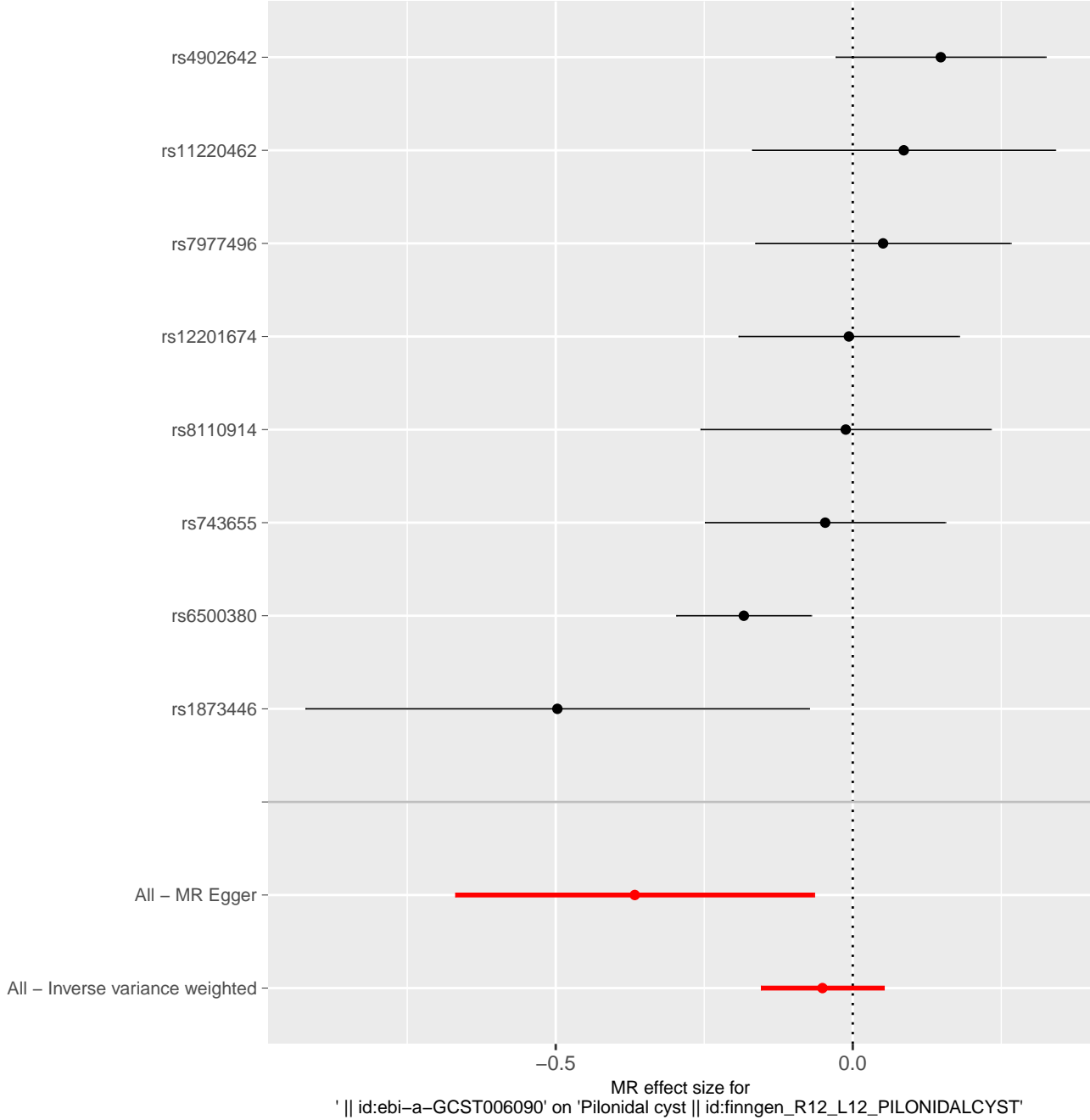

Supplement: Supplementary file 2 [file Datasheet2.zip › Supplementary Data 2/MR_pipeline_p5e-6/forest_plots/ebi-a-GCST006090.finngen_R12_L12_PILONIDALCYST.pdf]

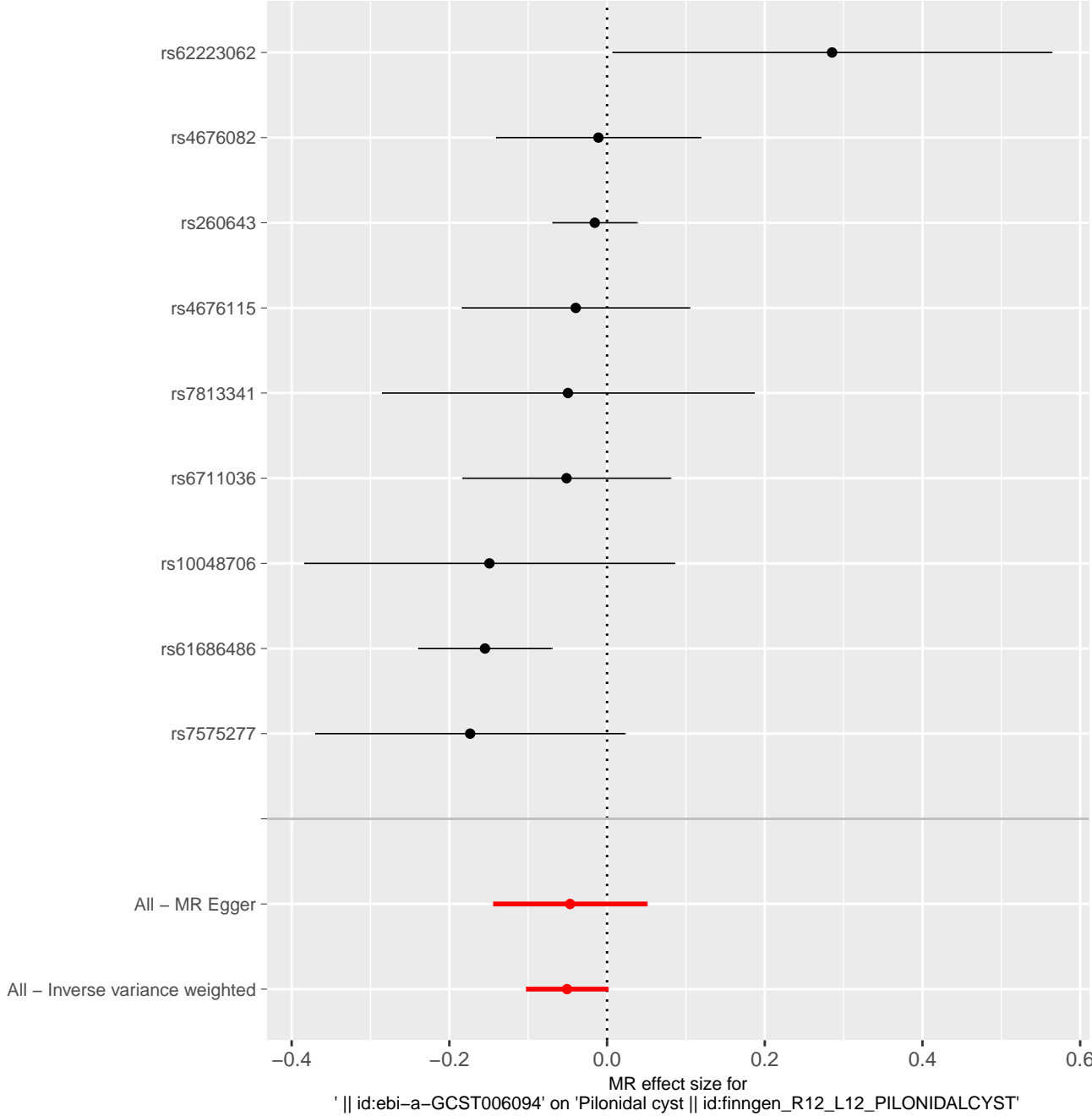

Supplement: Supplementary file 2 [file Datasheet2.zip › Supplementary Data 2/MR_pipeline_p5e-6/forest_plots/ebi-a-GCST006094.finngen_R12_L12_PILONIDALCYST.pdf]

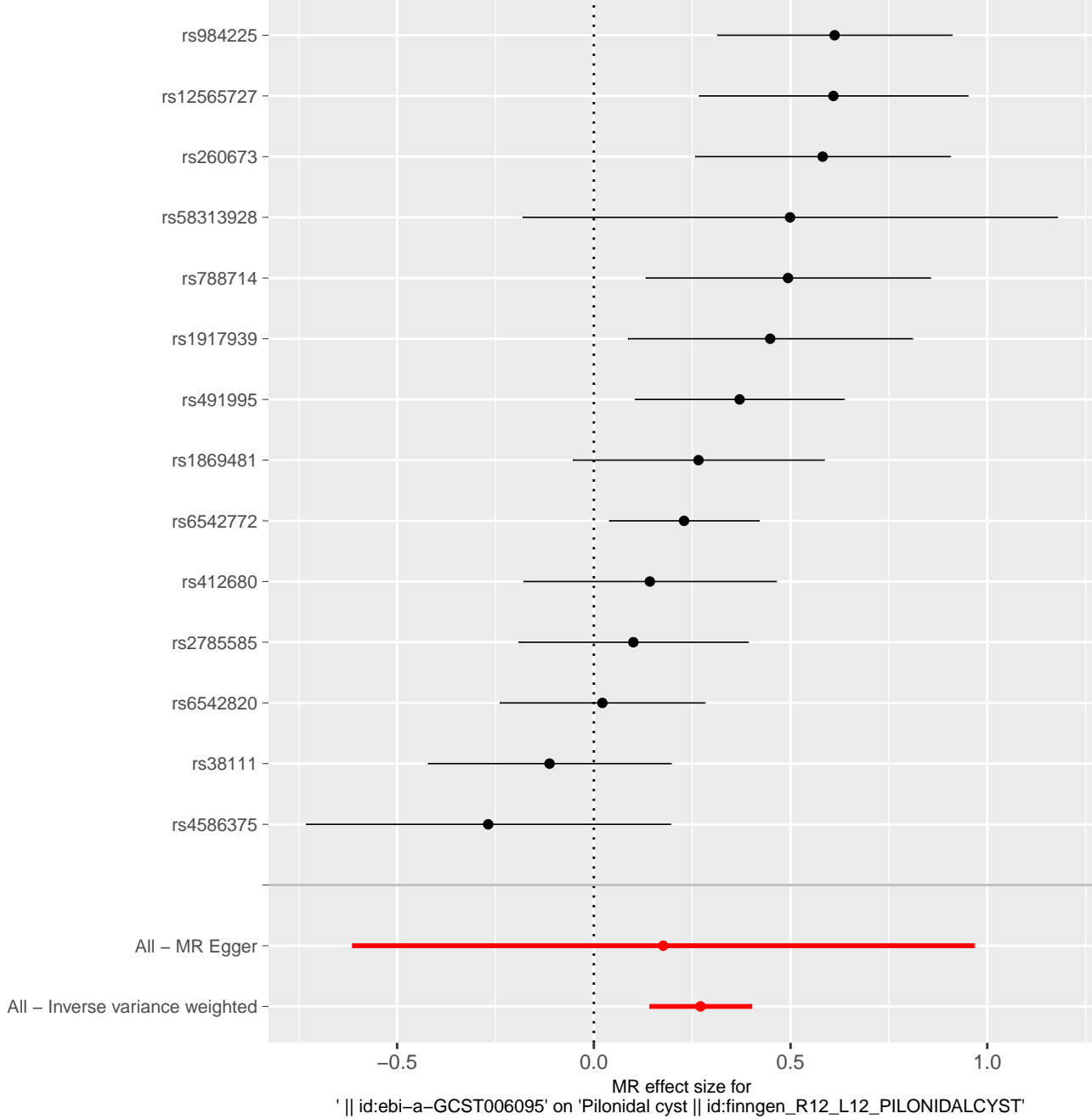

Supplement: Supplementary file 2 [file Datasheet2.zip › Supplementary Data 2/MR_pipeline_p5e-6/forest_plots/ebi-a-GCST006095.finngen_R12_L12_PILONIDALCYST.pdf]

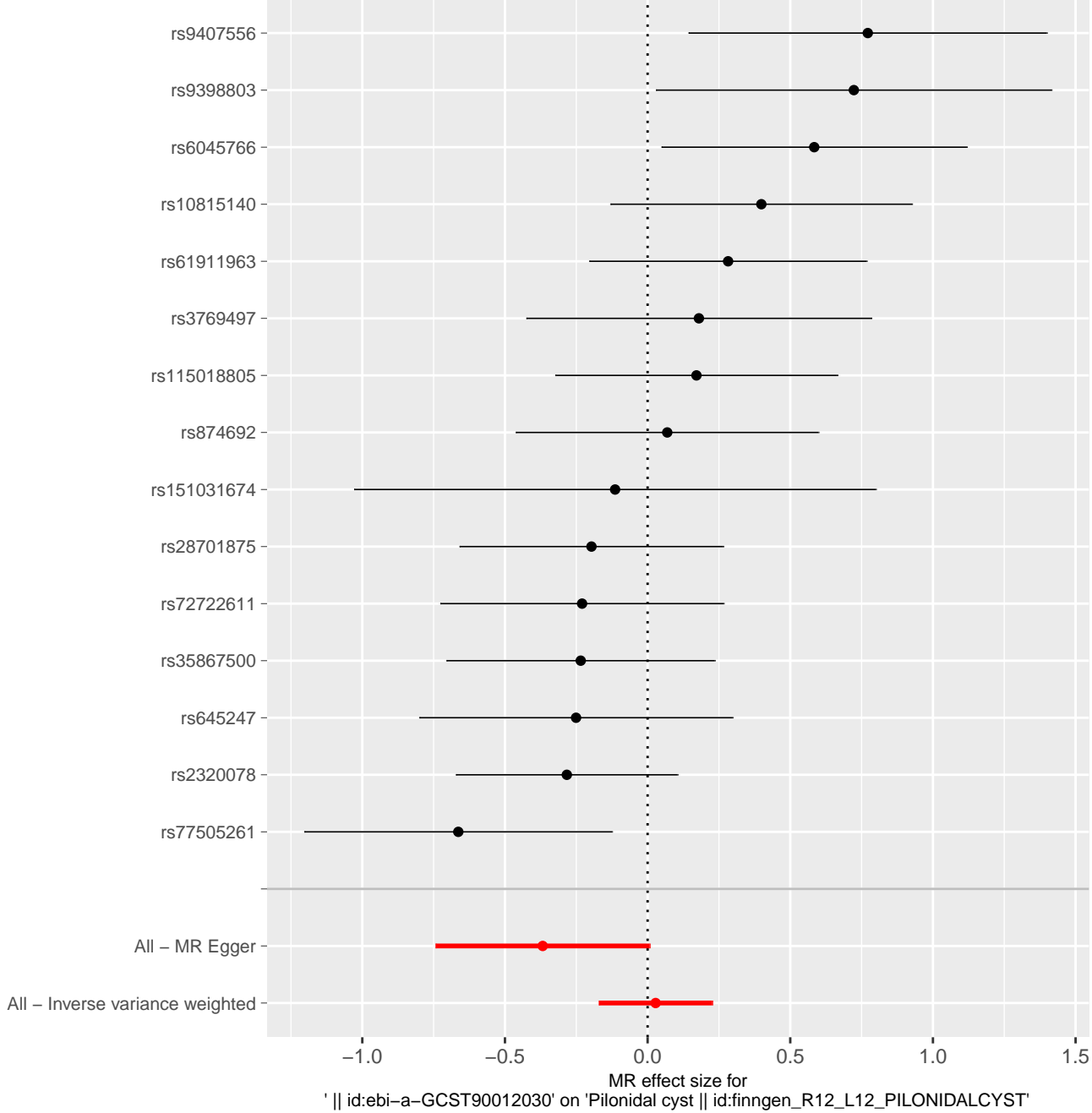

Supplement: Supplementary file 2 [file Datasheet2.zip › Supplementary Data 2/MR_pipeline_p5e-6/forest_plots/ebi-a-GCST90012030.finngen_R12_L12_PILONIDALCYST.pdf]

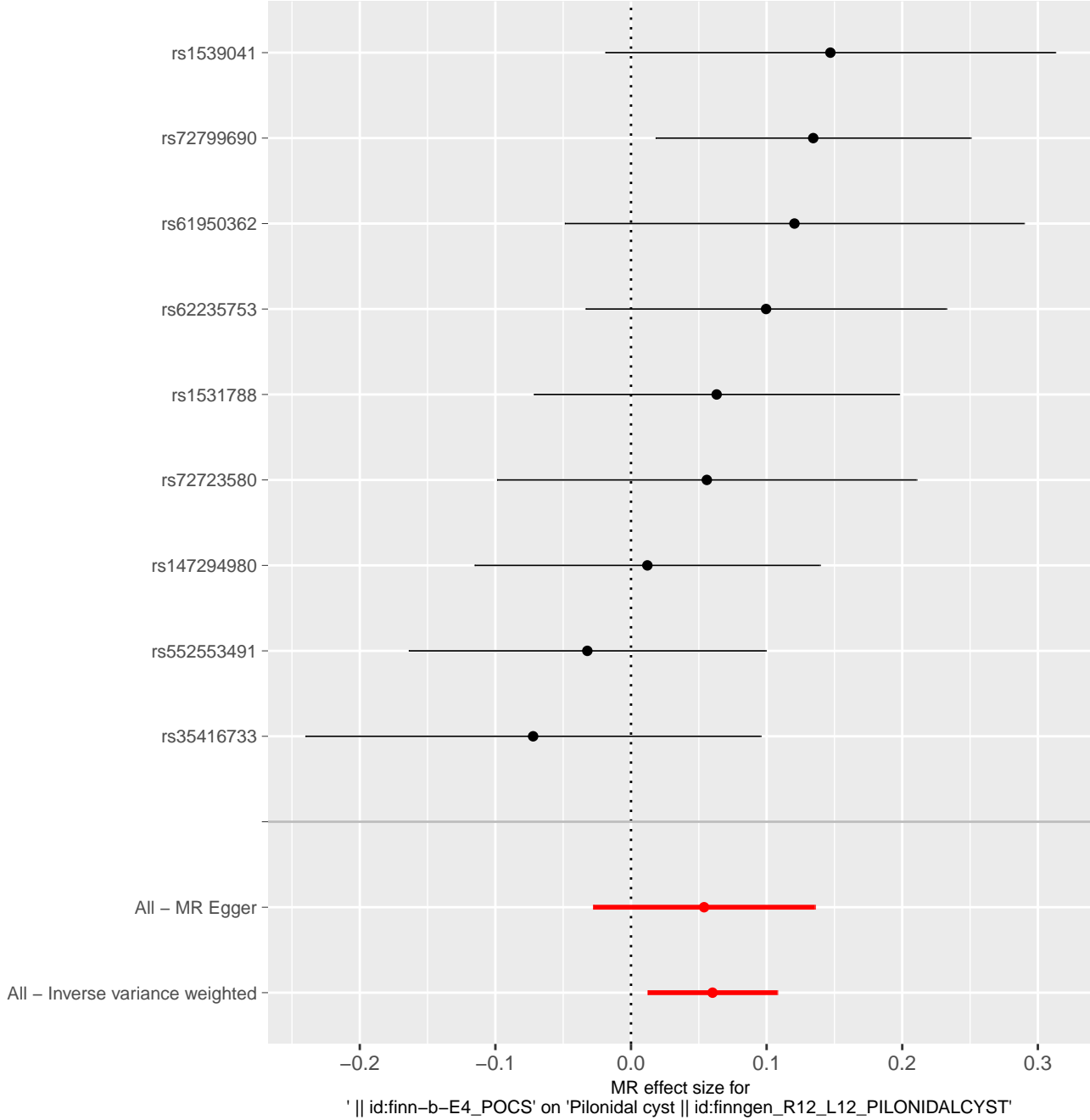

Supplement: Supplementary file 2 [file Datasheet2.zip › Supplementary Data 2/MR_pipeline_p5e-6/forest_plots/finn-b-E4_POCS.finngen_R12_L12_PILONIDALCYST.pdf]

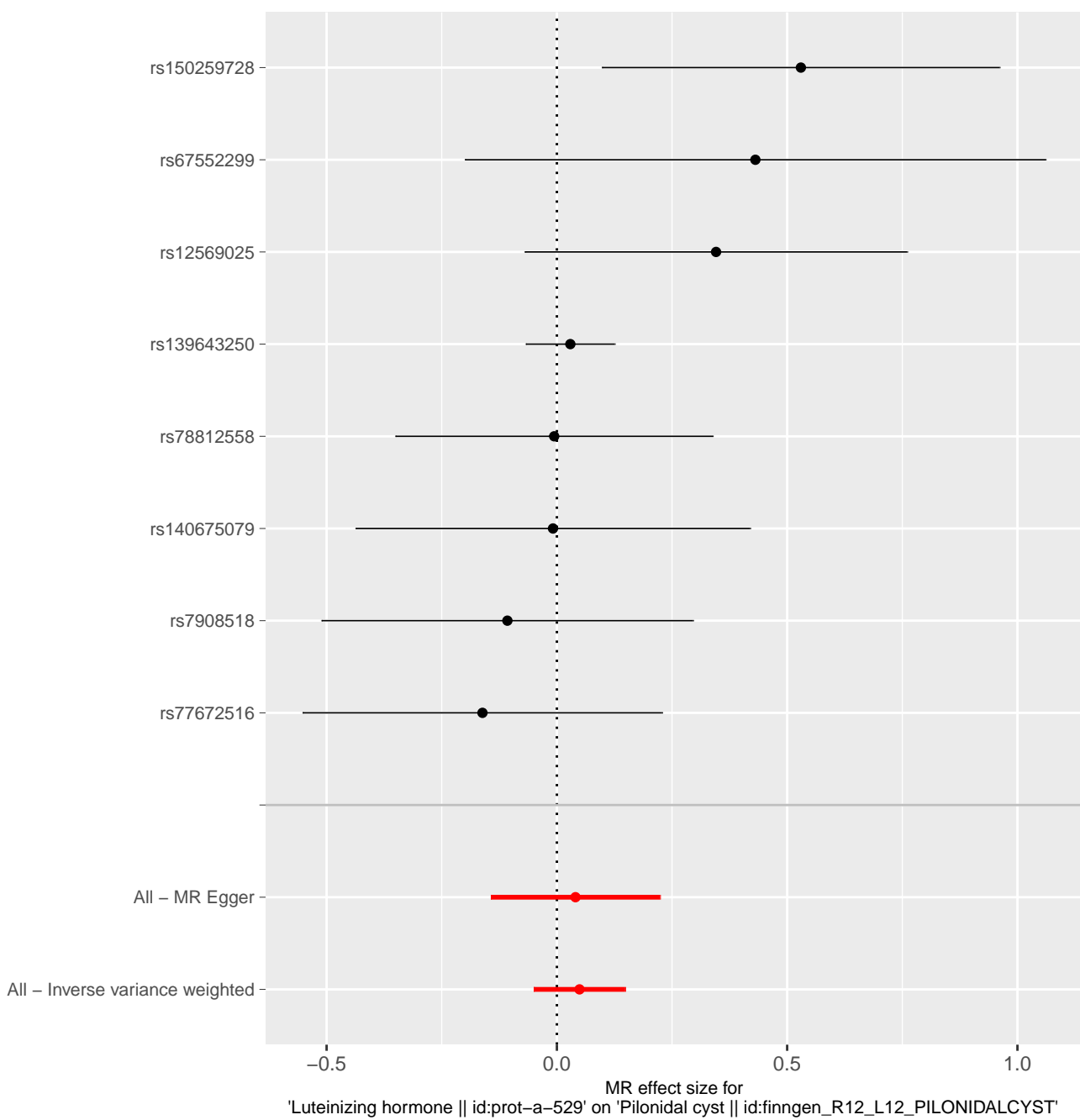

Supplement: Supplementary file 2 [file Datasheet2.zip › Supplementary Data 2/MR_pipeline_p5e-6/forest_plots/prot-a-529.finngen_R12_L12_PILONIDALCYST.pdf]

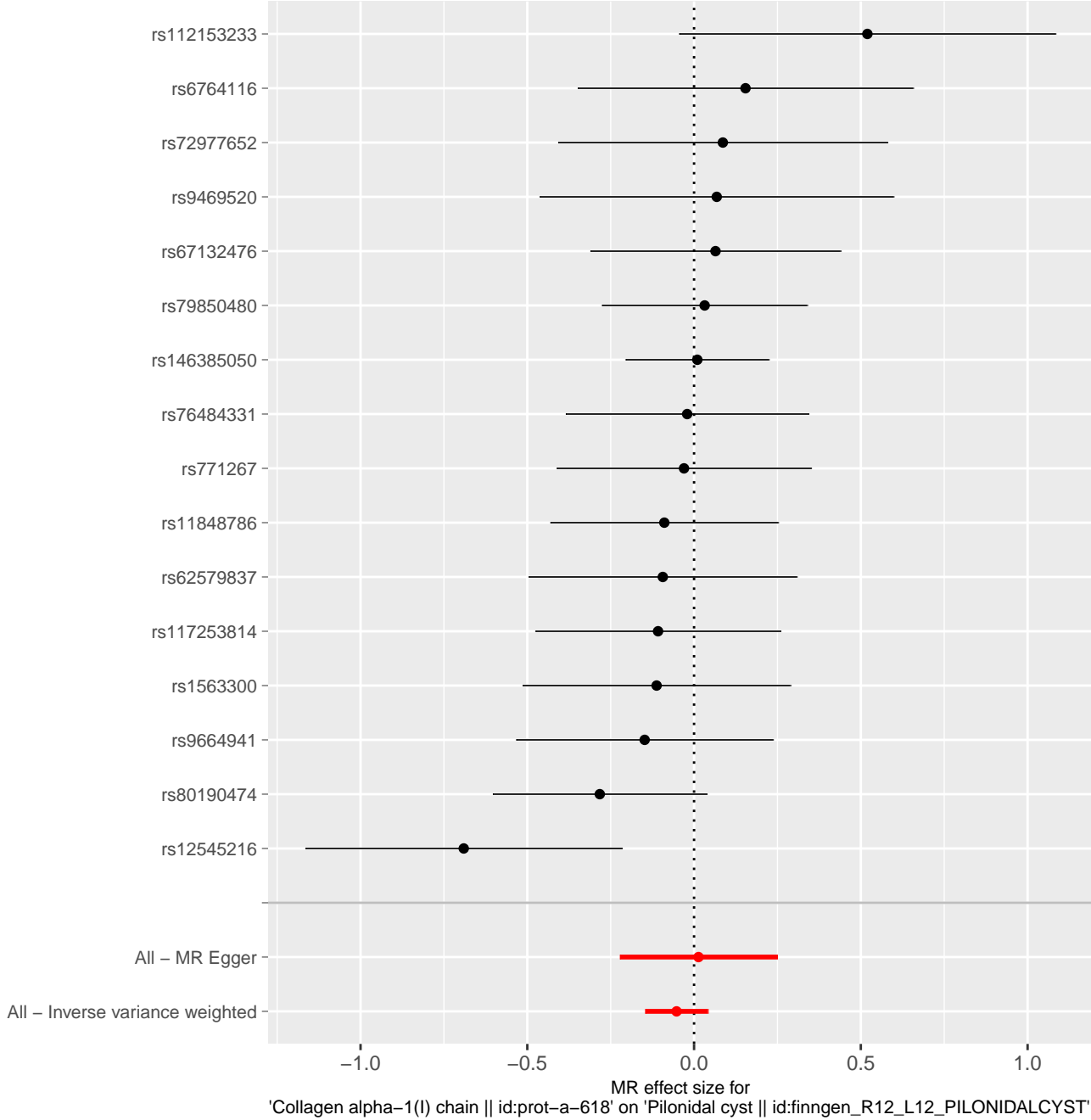

Supplement: Supplementary file 2 [file Datasheet2.zip › Supplementary Data 2/MR_pipeline_p5e-6/forest_plots/prot-a-618.finngen_R12_L12_PILONIDALCYST.pdf]

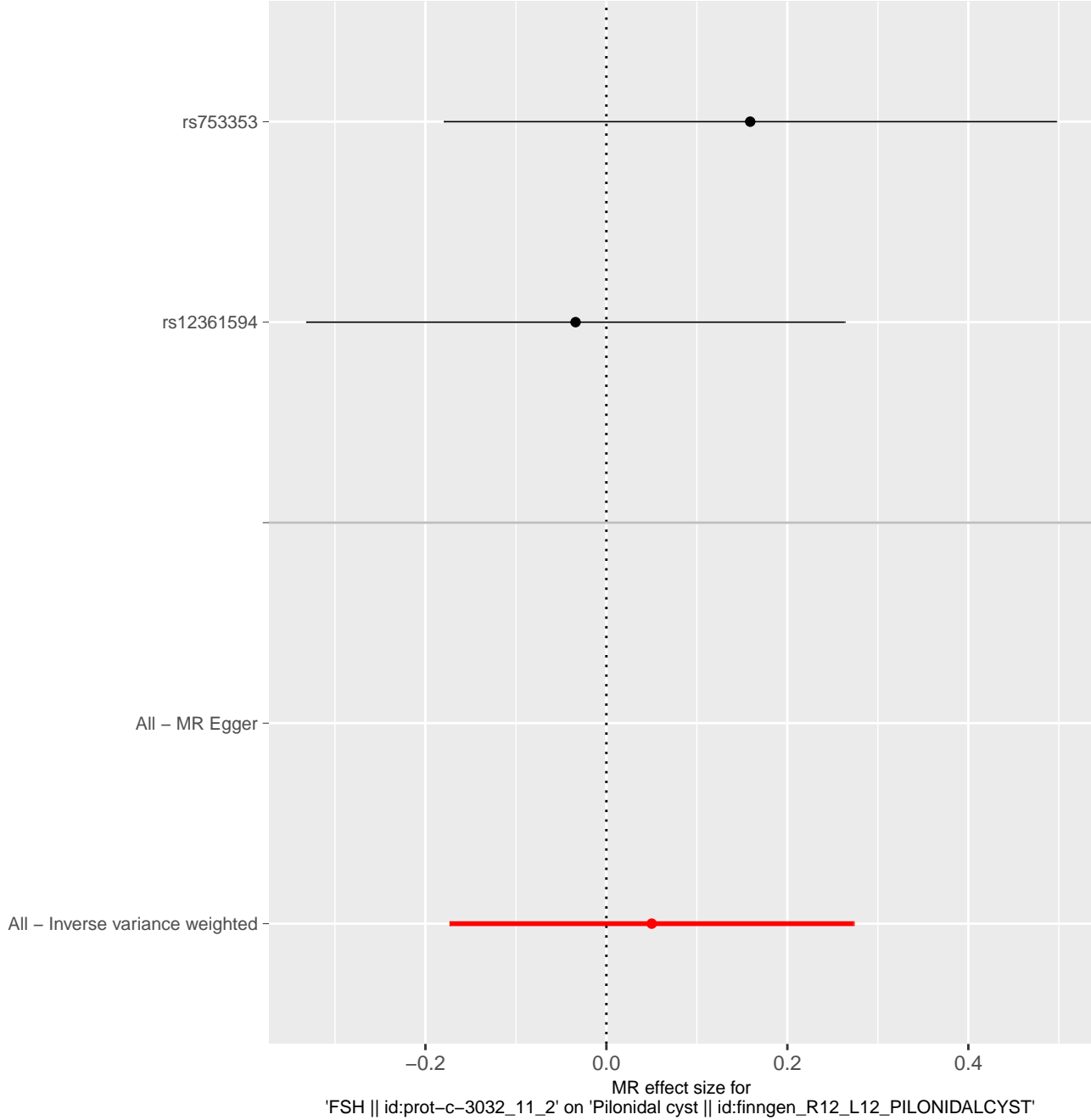

Supplement: Supplementary file 2 [file Datasheet2.zip › Supplementary Data 2/MR_pipeline_p5e-6/forest_plots/prot-c-3032_11_2.finngen_R12_L12_PILONIDALCYST.pdf]

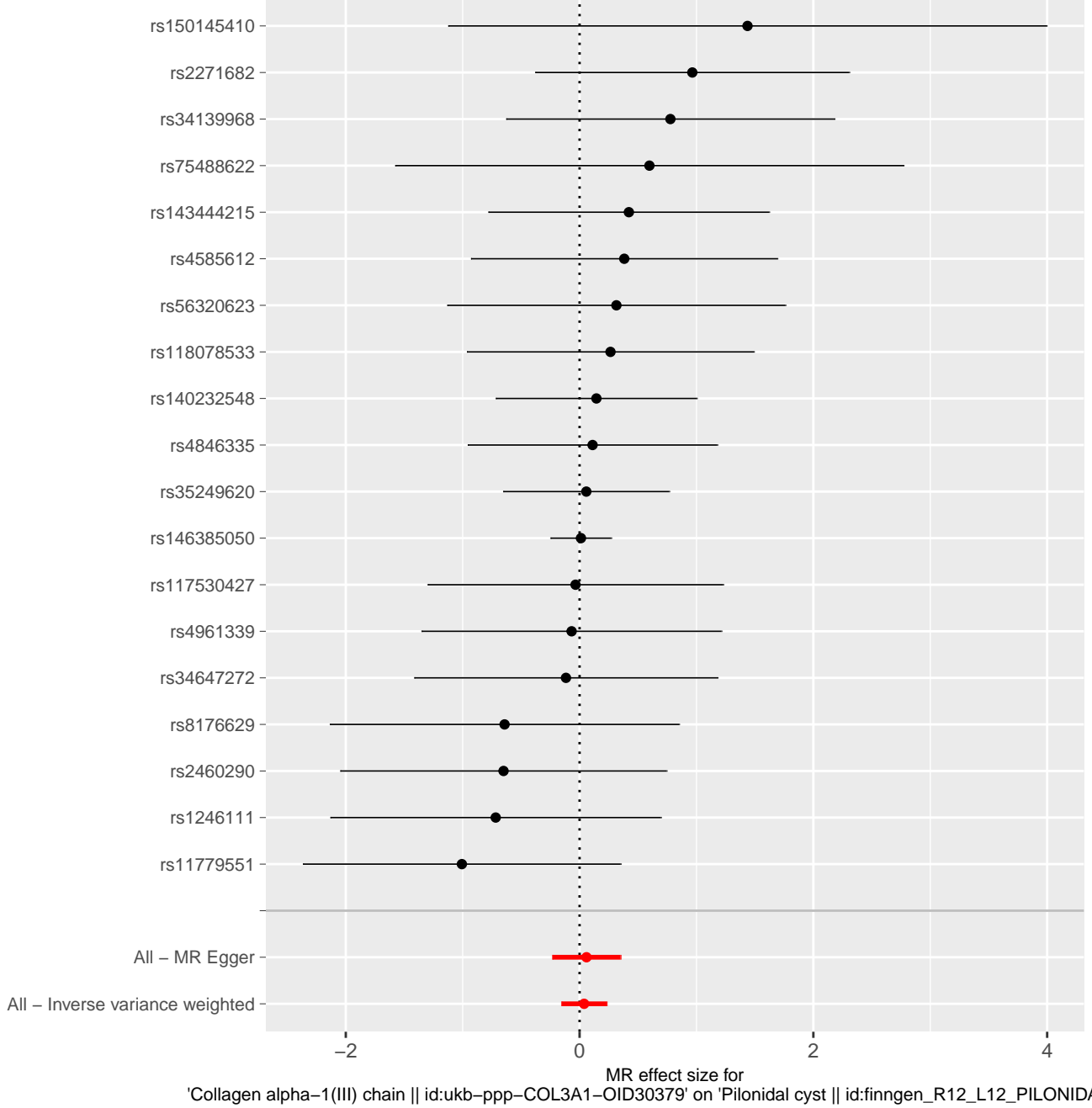

Supplement: Supplementary file 2 [file Datasheet2.zip › Supplementary Data 2/MR_pipeline_p5e-6/forest_plots/ukb-ppp-COL3A1-OID30379.finngen_R12_L12_PILONIDALCYST.pdf]
