# Supplementary material for: Risk factors for sacrococcygeal pilonidal sinus: a systematic review and meta-analysis supplemented by genetic causal assessment
Source: Front Surg. 2026 Jan 7;12:1718589. doi: 10.3389/fsurg.2025.1718589 (PMC12819706; doi:10.3389/fsurg.2025.1718589)
Supplement: Supplementary file 3 [file Datasheet3.docx]

#install packages

install.packages("devtools")

devtools::install_github("MRCIEU/TwoSampleMR")

#install.packages("ggplot2")

#library

library(TwoSampleMR)

library(ggplot2)

#set up your work directory

setwd("C:\\Users")

#load exposure data

expo_rt<- read_exposure_data(

filename = " ",

sep = "\t",

snp_col = "rsID",

beta_col = "beta",

se_col = "SE",

effect_allele_col = "eff.allele",

other_allele_col = "re.allele",

pval_col = "P",

samplesize_col = "N")

#data filter

expo_rt<- expo_rt[expo_rt$pval.exposure < 5e-8,]

expo_rt <- clump_data(expo_rt,clump_kb = 10000,

clump_r2 = 0.001)

write.table(expo_rt, " ",row.names = F,sep = "\t",quote = F)

#load outcome data

outc_rt <- read_outcome_data(

snps = expo_rt$SNP,

filename = " ",

sep = "\t",

snp_col = "variant_id",

beta_col = "beta",

se_col = "standard_error",

effect_allele_col = "effect_allele",

other_allele_col = "other_allele",

eaf_col = "effect_allele_frequency",

pval_col = "p_value")

#harmonise and merge data

harm_rt <- harmonise_data(

exposure_dat = ,

outcome_dat = ,action=1)

write.table(harm_rt, "harmonise.txt",row.names = F,sep = "\t",quote = F)

#mendelian randomization(MR) analysis

mr_result<- mr(harm_rt)

View(mr_result)

OR=generate_odds_ratios(mr_result)

write.table(OR[,5:ncol(OR)],"OR.txt",row.names = F,sep = "\t",quote = F)

#select relevant statistical methods

mr_method_list()

my_mr_result<- mr(harm_rt,method_list = c("mr_ivw","mr_egger_regression"))

View(my_mr_result)

OR2=generate_odds_ratios(my_mr_result)

write.table(OR2[,5:ncol(OR2)],"OR2.txt",row.names = F,sep = "\t",quote = F)

#heterogeneity test

mr_heterogeneity(harm_rt)

#outlier test

run_mr_presso(harm_rt,NbDistribution = 1000)

#pleiotropy test

mr_pleiotropy_test(harm_rt)

#obtain the beta for each SNP

singlesnp_res<- mr_singlesnp(harm_rt)

View(singlesnp_res)

singlesnpOR=generate_odds_ratios(singlesnp_res)

write.table(singlesnpOR,"singlesnpOR.txt",row.names = F,sep = "\t",quote = F)

#sensitivity analysis

sen_res<- mr_leaveoneout(harm_rt)

View(sen_res)

#Scatter plots of several statistical methods

p1 <- mr_scatter_plot(my_mr_result, harm_rt)

p1[[1]]

ggsave(p1[[1]], file="scatter.pdf", width=8, height=8)

#forest plot

p2 <- mr_forest_plot(singlesnp_res)

p2[[1]]

ggsave(p2[[1]], file="forest.pdf", width=8, height=8)

#sensitivity analysis plot

p3 <- mr_leaveoneout_plot(sen_res)

p3[[1]]

ggsave(p3[[1]], file="sensitivity analysis.pdf", width=8, height=8)

#funnel plot

res_single <- mr_singlesnp(harm_rt)

p4 <- mr_funnel_plot(singlesnp_res)

p4[[1]]

ggsave(p4[[1]], file="funnel plot.pdf", width=8, height=8)
