# Supplementary material for: Risk factors for sacrococcygeal pilonidal sinus: a systematic review and meta-analysis supplemented by genetic causal assessment
Source: Front Surg. 2026 Jan 7;12:1718589. doi: 10.3389/fsurg.2025.1718589 (PMC12819706; doi:10.3389/fsurg.2025.1718589)
Supplement: Supplementary file 5 [file Image1.pdf]

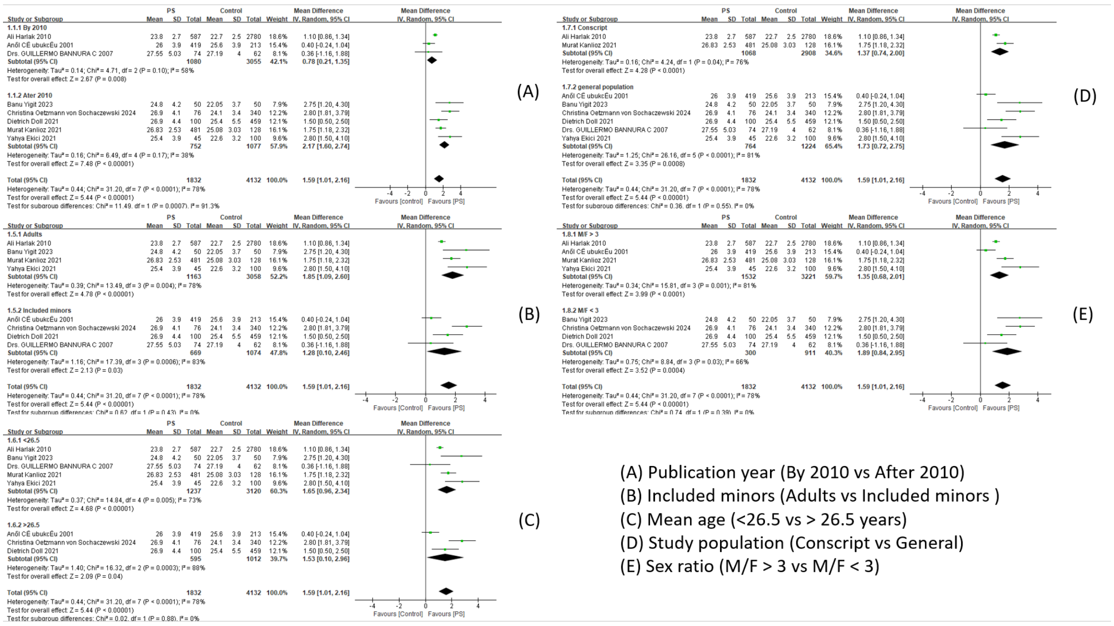

Supplementary Figure 1. Subgroup analyses of BMI differences according to study characteristics.

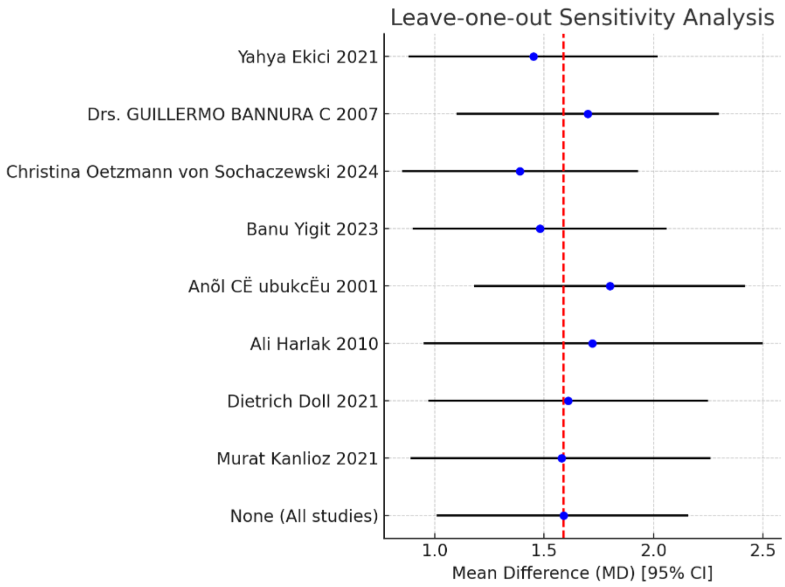

Supplementary Figure 2. Leave-one-out sensitivity analysis for the association between BMI and PS.

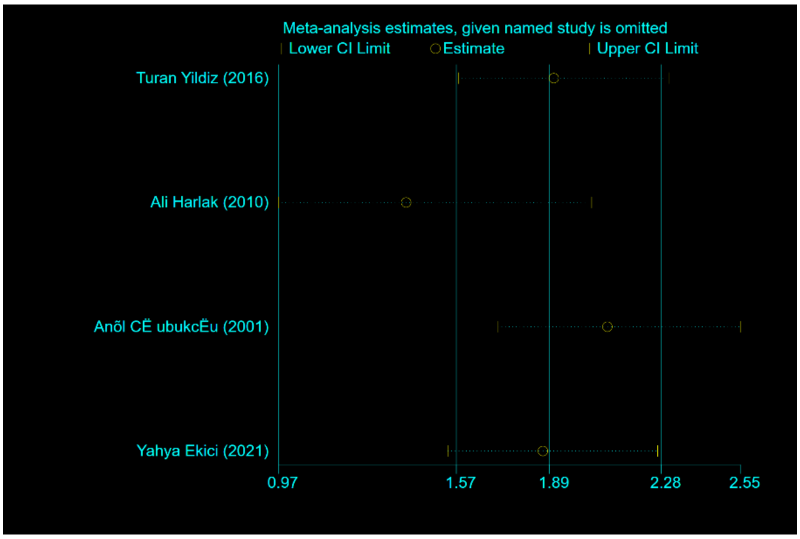

Supplementary Figure 3. Leave-one-out sensitivity analysis for the association between BMI and overweight.

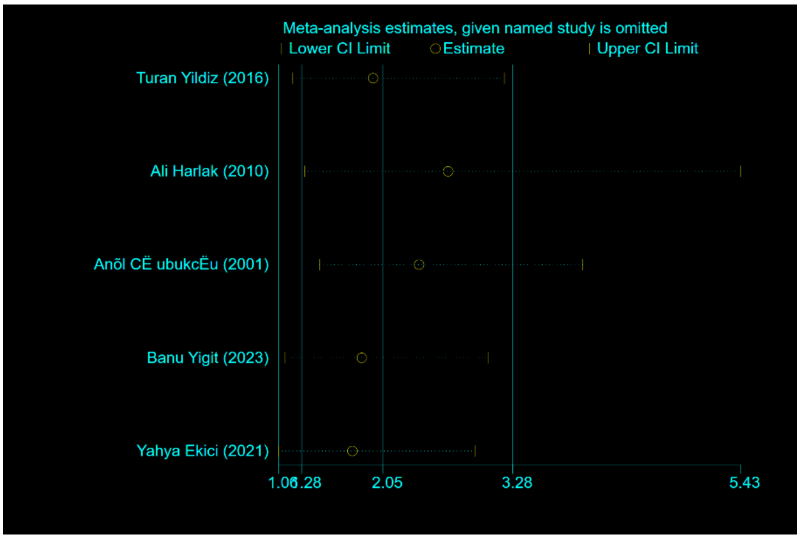

Supplementary Figure 4. Leave-one-out sensitivity analysis for the association between obesity and PS.

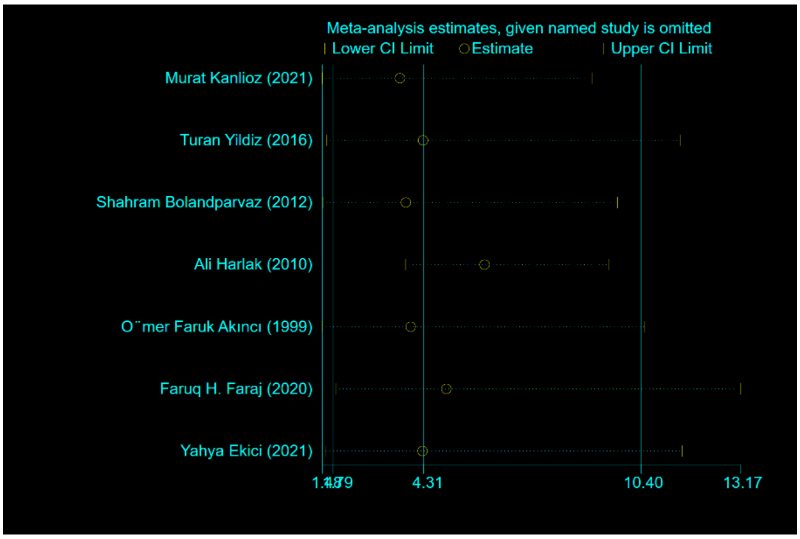

Supplementary Figure 5. Leave-one-out sensitivity analysis for the association between family history and PS.

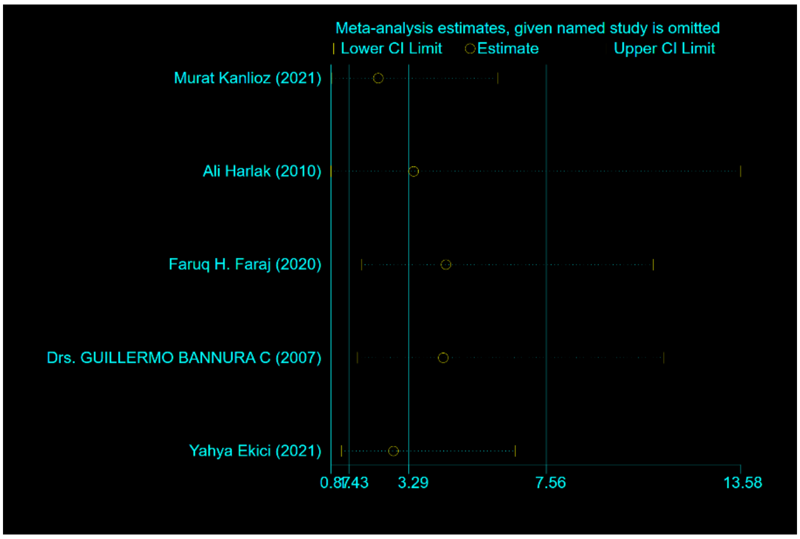

Supplementary Figure 6. Leave-one-out sensitivity analysis for the association between dense hair and PS.

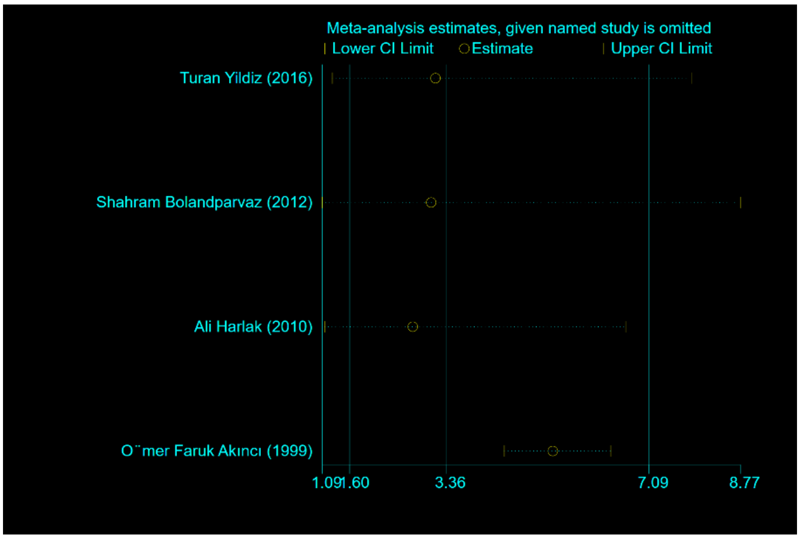

Supplementary Figure 7. Leave-one-out sensitivity analysis for low bathing frequency (≤2 times/week) and pilonidal sinus disease.

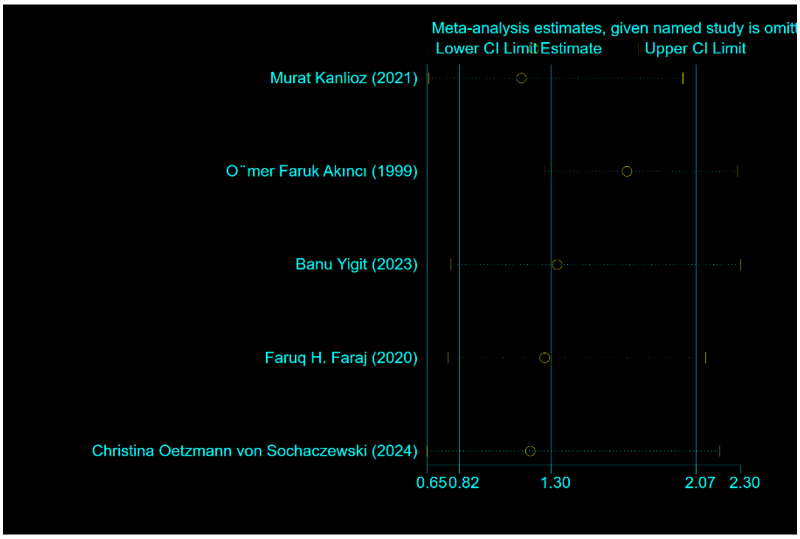

Supplementary Figure 8. Leave-one-out sensitivity analysis for the association between smoking and PS.

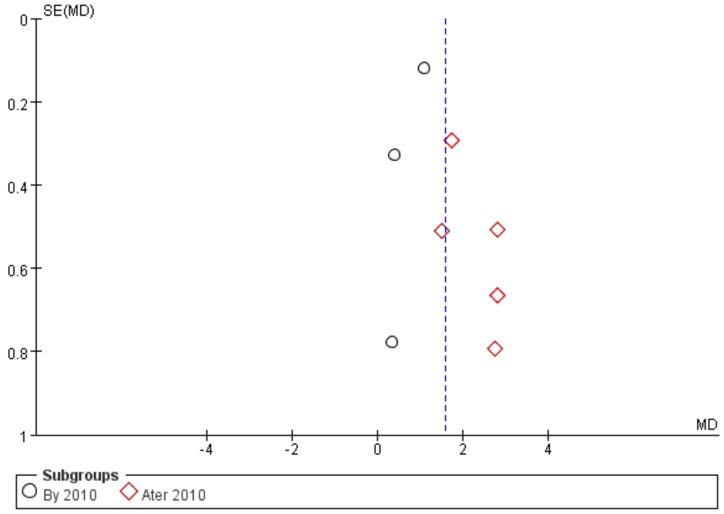

Supplementary Figure 9. Funnel plot assessing publication bias in the meta-analysis of BMI.

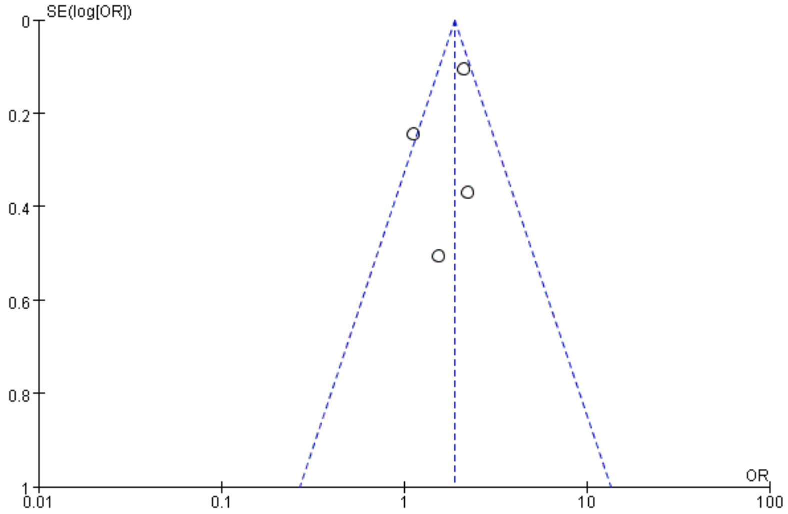

Supplementary Figure 10. Funnel plot assessing publication bias in the meta-analysis of overweight.

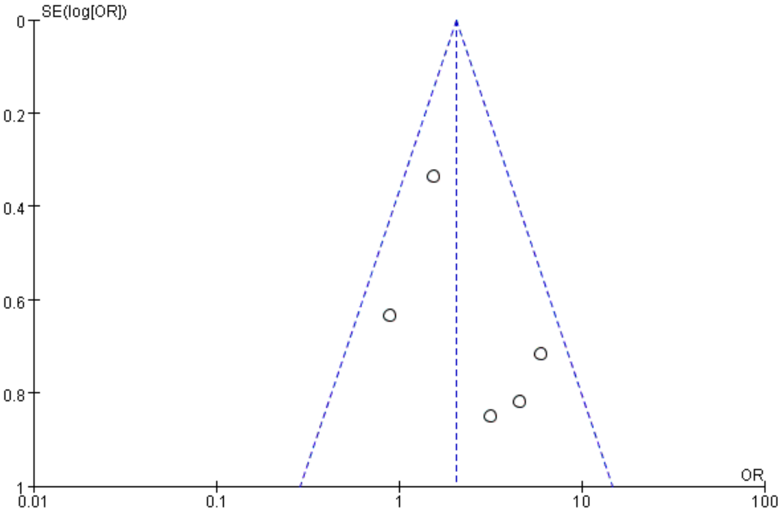

Supplementary Figure 11. Funnel plot assessing publication bias in the meta-analysis of obesity.

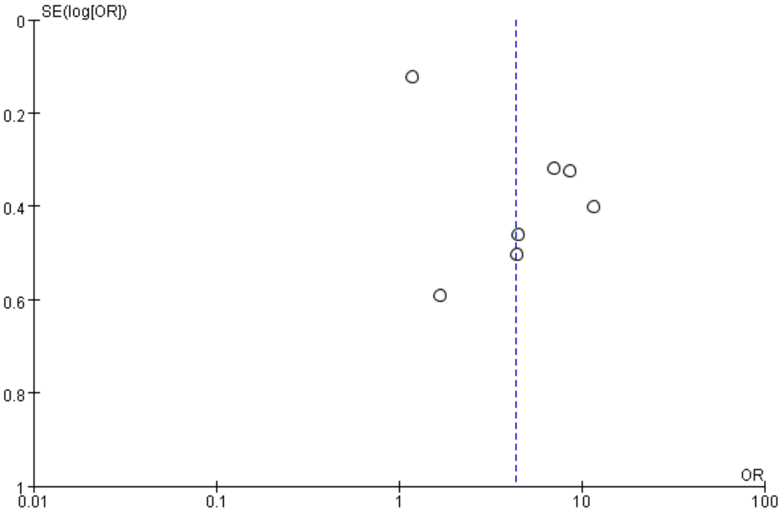

Supplementary Figure 12 (A). Funnel plot assessing publication bias in the meta-analysis of family history.

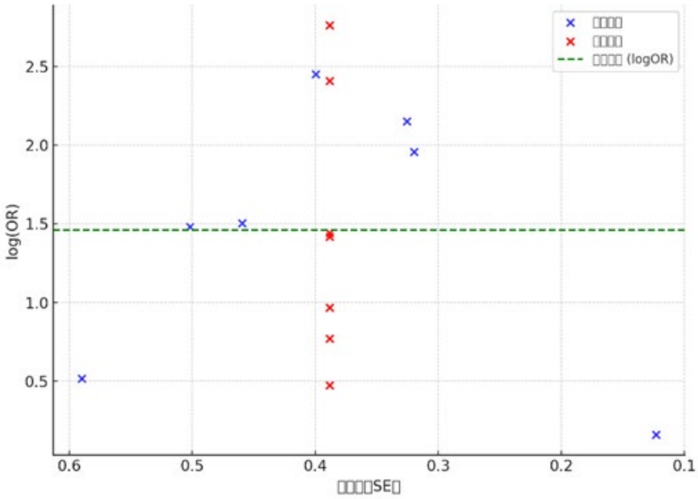

Supplementary Figure 12 (B). Trim-and-fill adjusted funnel plot for family history. Blue dots: original studies; red crosses: imputed studies; dashed line: adjusted pooled logOR ≈ 1.46.

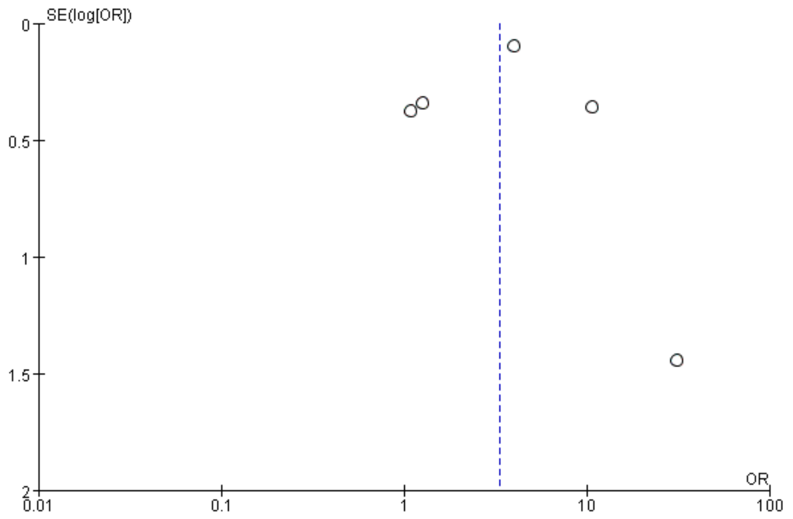

Supplementary Figure 13. Funnel plot assessing publication bias in the meta-analysis of dense hair.

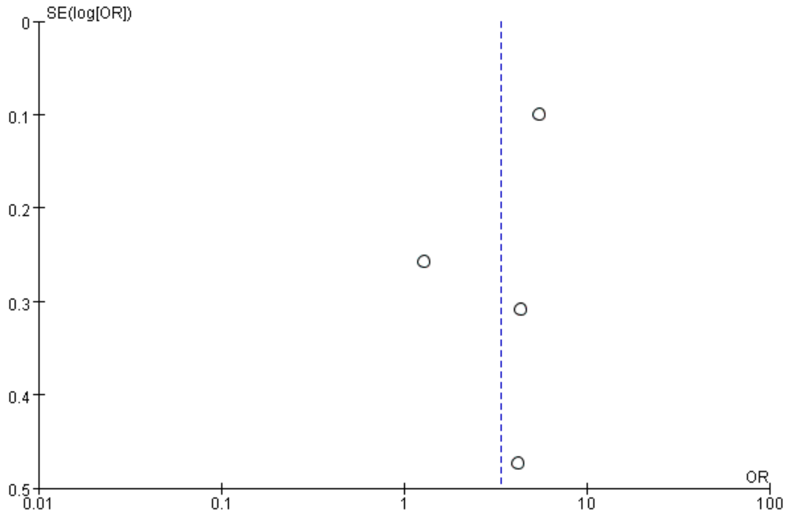

Supplementary Figure 14. Funnel plot assessing publication bias in the meta-analysis of low bathing frequency (≤2 times/week).

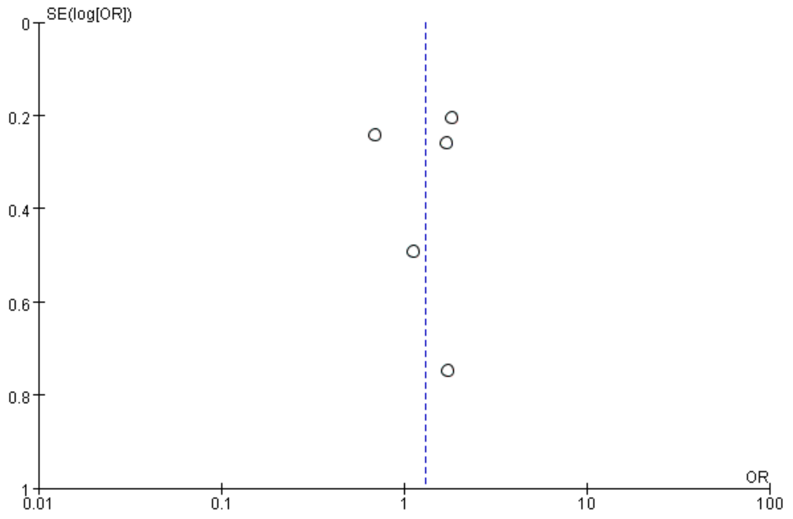

Supplementary Figure 15. Funnel plot assessing publication bias in the meta-analysis of

smoking.

Quality assessment							No of patients		Effect		Quality	Importance
No of studies	Design	Risk of bias	Inconsistency	Indirectness	Imprecision	Other considerations	BMI	Control	Relative (95% CI)	Absolute		
BMI (Better indicated by lower values)												
8	observational studies	no serious risk of bias	no serious inconsistency[1]	no serious indirectness	no serious imprecision	dose response gradient[2]	1832	4132	-	MD 1.59 higher (1.01 to 2.16 higher)	⊕⊕⊕O MODERATE	
Overweight												
4	observational studies	no serious risk of bias	no serious inconsistency[3]	no serious indirectness	no serious imprecision	dose response gradient[2]	257/1093 (23.5%)	489/3133 (15.6%)	OR 1.89 (1.57 to 2.28)	103 more per 1000 (from 69 more to 141 more)	⊕⊕⊕O MODERATE	
								18.8%		116 more per 1000 (from 79 more to 157 more)		
Obesity												
5	observational studies	no serious risk of bias	no serious inconsistency	no serious indirectness	no serious imprecision	strong association[4] dose response gradient[2]	40/1143 (3.5%)	48/3183 (1.5%)	OR 2.05 (1.28 to 3.28)	15 more per 1000 (from 4 more to 33 more)	⊕⊕⊕⊕ HIGH	
								3%		30 more per 1000 (from 8 more to 62 more)		

[1] Although the overall heterogeneity was substantial (I^2 = 78%), subgroup analyses reduced heterogeneity effectively, with I^2 values of 38% and 58% in the two subgroups, respectively. The test for subgroup differences was statistically significant ($P < 0.05$), indicating that the subgrouping explained part of the observed heterogeneity. Therefore, the inconsistency is considered acceptable, and no downgrade was applied for inconsistency in the GRADE assessment.

[2] In the present meta-analysis, a dose-response relationship was observed between BMI and the risk of pilonidal sinus. The pooled effect size was 1.89 for overweight individuals and increased further to 2.05 for those with obesity, indicating a progressive increase in disease risk with higher BMI.

[3] Although the pooled analysis showed a certain degree of statistical heterogeneity, leave-one-out sensitivity analysis indicated that the overall effect size and significance remained stable when any single study was removed. This suggests robust results. Therefore, no downgrade was applied for inconsistency.

[4] OR=2.05

Supplementary Figure 16. GRADE assessment of evidence quality for the associations between BMI, overweight, obesity, and PS.

Quality assessment							No of patients		Effect		Quality	Importance
No of studies	Design	Risk of bias	Inconsistency	Indirectness	Imprecision	Other considerations	家族史	Control	Relative (95% CI)	Absolute		
Family history （+）												
7	observational studies	no serious risk of bias	no serious inconsistency[1]	no serious indirectness	no serious imprecision	strong association[2]	425/1436 (29.6%)	490/4156 (11.8%)	OR 4.31 (1.78 to 10.43)	248 more per 1000 (from 74 more to 464 more)	⊕⊕⊕O MODERATE	
								10%		224 more per 1000 (from 65 more to 437 more)		

[1] Although the pooled analysis showed a certain degree of statistical heterogeneity, leave-one-out sensitivity analysis indicated that the overall effect size and significance remained stable when any single study was removed. This suggests robust results. Therefore, no downgrade was applied for inconsistency.

[2] OR=4.31

[3] Although publication bias was detected (Egger's test, $P = 0.043$), the effect size remained statistically significant after adjustment using the trim-and-fill method, suggesting robust results. Therefore, no downgrade was applied for publication bias.

Supplementary Figure 17. GRADE assessment of evidence quality for the associations between family history and PS.

Quality assessment							No of patients		Effect		Quality	Importance
No of studies	Design	Risk of bias	Inconsistency	Indirectness	Imprecision	Other considerations	毛发浓密	Control	Relative (95% CI)	Absolute		
毛多												
5	observational studies	no serious risk of bias	no serious inconsistency[1]	no serious indirectness	no serious imprecision	strong association[2] dose response gradient[3]	550/1281 (42.9%)	521/3165 (16.5%)	OR 3.29 (1.43 to 7.6)	229 more per 1000 (from 55 more to 435 more)	⊕⊕⊕⊕ HIGH	
								17.9%		239 more per 1000 (from 59 more to 445 more)		

[1] Although the pooled analysis showed a certain degree of statistical heterogeneity, leave-one-out sensitivity analysis indicated that the overall effect size and significance remained stable when any single study was removed. This suggests robust results. Therefore, no downgrade was applied for inconsistency.

[2] OR=3.29

[3] The study demonstrated a positive dose-response relationship between hair density and the risk of pilonidal sinus disease, with higher hair density associated with an increased risk. This supports an upgrade under the "dose-response gradient" domain in the GRADE assessment.

Supplementary Figure 18. GRADE assessment of evidence quality for the associations between dense hair and PS.

Quality assessment							No of patients		Effect		Quality	Importance
No of studies	Design	Risk of bias	Inconsistency	Indirectness	Imprecision	Other considerations	局部卫生	Control	Relative (95% CI)	Absolute		
Bathing frequency ≤2 times per week												
4	observational studies	no serious risk of bias	no serious inconsistency[1]	no serious indirectness	no serious imprecision	strong association[2] dose response gradient[3]	586/816 (71.8%)	1558/3833 (40.6%)	OR 3.36 (1.6 to 7.09)	291 more per 1000 (from 116 more to 423 more)	⊕⊕⊕⊕ HIGH	
								35.9%		294 more per 1000 (from 114 more to 440 more)		

[1] Although the pooled analysis showed a certain degree of statistical heterogeneity, leave-one-out sensitivity analysis indicated that the overall effect size and significance remained stable when any single study was removed. This suggests robust results. Therefore, no downgrade was applied for inconsistency.

[2] OR=3.36

[3] Higher bathing frequency indicates better sacrococcygeal hygiene and a lower risk of pilonidal sinus disease.

Supplementary Figure 19. GRADE assessment of evidence quality for the associations between low bathing frequency (≤2 times/week) and PS.

Quality assessment							No of patients		Effect		Quality	Importance
No of studies	Design	Risk of bias	Inconsistency	Indirectness	Imprecision	Other considerations	烟酒	Control	Relative (95% CI)	Absolute		
Smoking												
5	observational studies	no serious risk of bias	no serious inconsistency[1]	no serious indirectness	no serious imprecision	none	348/789 (44.1%)	853/1525 (55.9%)	OR 1.3 (0.82 to 2.07)	63 more per 1000 (from 49 fewer to 165 more)	⊕⊕OO LOW	
								30.9%		59 more per 1000 (from 41 fewer to 172 more)		

[1] Although the pooled analysis showed a certain degree of statistical heterogeneity, leave-one-out sensitivity analysis indicated that the overall effect size and significance remained stable when any single study was removed. This suggests robust results. Therefore, no downgrade was applied for inconsistency.

Supplementary Figure 20. GRADE assessment of evidence quality for the associations between smoking and PS.
